# Supplementary material for: Evaluating equity dimensions of infant and child vitamin A supplementation programmes using Demographic and Health Surveys from 49 countries
Source: BMJ Open. 2023 Mar 14;13(3):e062387. doi: 10.1136/bmjopen-2022-062387 (PMC10016247; doi:10.1136/bmjopen-2022-062387)
Supplement: Supplementary data [file bmjopen-2022-062387supp001.pdf]

Evaluating equity dimensions of infant and child vitamin A supplementation programs using Demographic and Health Surveys from 49 countries

- SUPPLEMENTARY MATERIAL –

Table of Contents

Supplementary Table 1. Countries included in the study and summary information on their respective Demographic and Health Surveys 2

Supplementary Table 2. Descriptive summary of the survey population of children aged 6-59 months for each country included in this study 4

Supplementary Figure 1. Vitamin A supplementation (VAS) coverage between administrative regions for included countries in East and Southern Africa. 6

Supplementary Figure 2. Vitamin A supplementation (VAS) coverage between administrative regions for included countries in West and Central Africa. 9

Supplementary Figure 3. Vitamin A supplementation (VAS) coverage between administrative regions for included countries in South Asia, East Asia, and the Western Pacific. 13

Supplementary Figure 4. Vitamin A supplementation (VAS) coverage between administrative regions for included countries in the Eastern Mediterranean Region. 16

Supplementary Figure 5. Vitamin A supplementation (VAS) coverage between administrative regions for included countries in the Americas. 17

Supplementary Figure 6. Vitamin A supplementation (VAS) coverage among children from households of the poorest versus the richest socioeconomic quintiles 18

Supplementary Figure 7. Differences in vitamin A supplementation (VAS) coverage between children whose caregivers are the least versus the most socially independent in their country. 19

Supplementary Figure 8. Differences in vitamin A supplementation (VAS) coverage between children whose caregivers have the least versus the most decision-making autonomy in their country. 20

Supplementary Figure 9. Differences in vitamin A supplementation (VAS) coverage between children based on the educational attainment of the child's caregiver 21

Supplementary Figure 10. Proportion of children who had received vitamin A supplementation (VAS) whose caregiver confirmed VAS reception with a vaccination card. 22

Tang K, *et al. BMJ Open* 2023; 13:e062387. doi: 10.1136/bmjopen-2022-062387

Supplementary Table 1. Countries included in the study and summary information on their respective Demographic and Health Surveys

Country	Survey year	Women surveyed (n)	Children recalled (n)
Afghanistan	2015	29,461	29,312
Bangladesh	2017	20,127	4137
Benin	2017	15,928	6378
Burkina Faso	2010	17,087	13,644
Burundi	2016	17,269	6161
Cambodia	2014	17,578	6909
Cameroon	2018	13,527	4337
Chad	2014	17,719	16,441
Comoros	2012	5329	2879
Congo	2011	10,819	8725
Cote D'Ivoire	2012	10,060	6882
Dem. Rep. of the Congo	2013	18,827	16,952
Ethiopia	2016	15,683	9504
Gabon	2012	8422	5747
Gambia	2019	11,865	3769
Ghana	2014	9396	5531
Guatemala	2015	25,914	12,071
Guinea	2018	10,874	3288
Haiti	2016	14,371	2922
Honduras	2011	22,757	10,592
India	2015	699,686	244,869
Indonesia	2017	45,607	17,367
Kenya	2014	31,079	19,887
Kyrgyzstan	2012	8208	4247
Lesotho	2014	6621	2855
Liberia	2019	8065	2493
Malawi	2015	24,562	7870
Mali	2018	10,519	4442
Mozambique	2011	13,745	10,214
Myanmar	2016	12,885	4550
Namibia	2013	9176	4818
Nepal	2016	12,862	2354
Niger	2012	11,160	11,338
Nigeria	2018	41,821	14,434
Pakistan	2017	15,068	5589
Papua New Guinea	2017	15,198	4436
Philippines	2017	25,074	4882
Rwanda	2019	14,634	7314
Senegal	2019	8649	3147
Sierra Leone	2019	15,574	4344
South Africa	2016	8514	3413
Tajikistan	2017	10,718	3016
Tanzania	2015	13,266	4864
Timor-Leste	2016	12,607	3332
Togo	2013	9480	6467
Uganda	2016	18,506	7138
Yemen	2013	25,434	15,136

Zambia	2018	13,683	4616
Zimbabwe	2015	9955	2775

Tang K, *et al. BMJ Open* 2023; 13:e062387. doi: 10.1136/bmjopen-2022-062387

Supplementary Table 2. Descriptive summary of the survey population of children aged 6-59 months for each country included in this study

Country	Received VAS in past 6 months, % (95%CI)	Did not recently consume vitamin A rich foods	Did not received DTP1	Did not received MCV1	Rural
Afghanistan	45.1 (40.9, 49.4)	62.5 (59.7, 65.2)	35.8 (31.9, 39.9)	49.3 (46.1, 52.6)	76.8 (71.4, 81.4)
Bangladesh	79.2 (77.6, 80.8)	41.8 (39.9, 43.7)	1.5 (1.0, 2.3)	19.7 (18.3, 21.3)	71.4 (68.4, 76.1)
Benin	51.4 (48.9, 53.9)	53.8 (51.9, 55.8)	16.2 (14.4, 18.3)	42.7 (40.5, 65.9)	61.3 (56.6, 65.9)
Burkina Faso	60.4 (57.8, 65.9)	73.6 (71.8, 75.4)	12.6 (11.3, 13.9)	28.7 (27.3, 30.1)	82.7 (79.3, 85.6)
Burundi	68.1 (66.2, 69.9)	33.1 (31.6, 34.7)	1.3 (1.0, 1.7)	17.3 (16.2, 18.4)	91.1 (88.2, 93.3)
Cambodia	63.3 (61.1, 65.4)	35.2 (32.7, 37.9)	9.7 (8.6, 10.9)	28.5 (26.9, 30.1)	85.3 (82.0, 88.1)
Cameroon	52.7 (50.3, 55.2)	41.4 (39.3, 43.4)	17.0 (14.6, 19.6)	39.5 (36.9, 42.2)	55.3 (49.1, 61.4)
Chad	40.6 (38.3, 43.0)	65.2 (62.9, 67.4)	47.5 (44.8, 50.3)	47.4 (45.3, 49.5)	80.4 (76.5, 83.9)
Comoros	43.2 (39.8, 46.6)	43.7 (39.8, 47.6)	24.4 (21.5, 27.6)	36.9 (33.9, 40.0)	72.7 (65.6, 78.8)
Congo	60.3 (57.4, 63.1)	40.5 (37.7, 43.3)	24.9 (22.8, 27.2)	35.5 (33.3, 37.8)	39.5 (33.4, 46.0)
Cote D'Ivoire	57.0 (53.8, 60.3)	54.1 (51.6, 56.7)	26.5 (23.6, 29.6)	43.7 (40.9, 46.5)	62.3 (55.6, 68.5)
Dem. Rep. of the Congo	64.8 (62.3, 67.1)	37.1 (35.0, 39.2)	22.5 (20.3, 24.9)	36.0 (34.2, 37.9)	69.1 (63.4, 74.3)
Ethiopia	40.1 (37.9, 42.8)	69.9 (67.0, 72.7)	27.8 (25.0, 30.9)	60.2 (57.2, 63.1)	88.8 (85.4, 91.5)
Gabon	50.1 (46.7, 53.6)	48.2 (45.1, 51.2)	47.4 (44.5, 50.3)	36.2 (33.6, 38.8)	15.6 (11.9, 20.1)
Gambia	54.1 (51.6, 56.5)	56.3 (53.5, 59.0)	2.4 (1.7, 3.2)	19.8 (17.9, 21.9)	34.1 (27.6, 41.2)
Ghana	58.7 (55.6, 61.7)	49.0 (45.7, 52.2)	7.2 (6.2, 8.5)	24.9 (23.3, 26.6)	54.9 (48.5, 61.1)
Guatemala	43.0 (41.3, 44.7)	19.2 (18.2, 20.3)	8.6 (7.9, 9.3)	30.0 (29.0, 31.0)	64.0 (59.9, 67.9)
Guinea	40.0 (37.3, 42.6)	66.7 (64.1, 69.1)	36.6 (33.2, 40.1)	60.1 (56.5, 63.7)	71.0 (65.9, 75.7)
Haiti	28.3 (26.3, 30.4)	55.9 (53.1, 58.6)	19.3 (16.8, 22.0)	42.7 (39.8, 45.6)	64.4 (58.6, 69.9)
Honduras	63.5 (62.0, 64.9)	32.2 (30.4, 34.0)	4.8 (4.4, 5.3)	24.5 (23.6, 25.5)	53.8 (50.2, 57.3)
India	55.1 (54.6, 55.6)	38.2 (37.6, 38.7)	15.0 (14.7, 15.3)	27.7 (25.3, 30.1)	68.1 (62.1, 73.6)
Indonesia	65.6 (64.2, 67.0)	34.3 (32.6, 36.0)	16.5 (15.3, 17.7)	30.2 (28.9, 31.5)	50.1 (46.7, 53.6)
Kenya	67.2 (65.9, 68.5)	41.3 (38.9, 43.8)	5.7 (5.2, 6.2)	24.8 (23.8, 25.7)	64.3 (60.9, 67.6)
Kyrgyzstan	39.0 (36.1, 41.9)	47.2 (43.9, 50.6)	4.0 (3.2, 5.1)	19.0 (17.6, 20.4)	70.1 (63.4, 76.1)
Lesotho	54.4 (51.6, 57.1)	52.6 (48.8, 56.4)	6.6 (5.5, 7.9)	24.1 (22.3, 26.0)	71.0 (64.2, 76.9)
Liberia	45.1 (41.1, 49.1)	54.7 (51.6, 57.8)	9.4 (7.5, 11.6)	35.9 (32.7, 39.2)	47.2 (39.4, 55.0)
Malawi	63.9 (62.5, 65.3)	38.9 (37.5, 40.4)	2.6 (2.1, 3.2)	18.4 (17.3, 19.5)	86.7 (83.3, 89.5)
Mali	66.9 (64.4, 69.3)	55.2 (52.9, 57.6)	17.4 (15.2, 19.9)	36.7 (34.0, 39.4)	80.0 (74.7, 84.3)

Tang K, *et al. BMJ Open* 2023; 13:e062387. doi: 10.1136/bmjopen-2022-062387

Mozambique	66.3 (64.2, 68.4)	35.7 (33.7, 37.6)	15.0 (13.2, 16.9)	30.1 (28.2, 32.1)	72.2 (67.8, 76.2)
Myanmar	49.6 (46.6, 52.5)	46.2 (42.9, 49.6)	18.0 (15.6, 20.7)	31.3 (28.8, 33.9)	77.4 (72.3, 81.9)
Namibia	79.2 (77.4, 81.0)	46.1 (43.2, 49.0)	8.7 (7.6, 9.9)	22.1 (20.6, 23.6)	51.0 (45.6, 56.4)
Nepal	82.5 (80.4, 84.4)	43.7 (40.3, 47.1)	3.3 (2.3, 4.8)	18.8 (16.8, 20.9)	46.1 (39.5, 52.8)
Niger	55.5 (52.8, 58.1)	72.7 (70.6, 74.8)	22.5 (20.5, 24.6)	41.1 (39.3, 42.9)	86.3 (83.2, 89.0)
Nigeria	44.7 (42.6, 46.8)	53.7 (52.1, 55.3)	36.1 (34.0, 38.3)	50.7 (48.8, 52.6)	61.4 (58.0, 64.8)
Pakistan	75.1 (72.5, 77.6)	59.0 (56.0, 62.0)	15.9 (12.9, 19.4)	33.6 (30.4, 36.9)	68.0 (62.1, 73.4)
Papua New Guinea	28.2 (25.9, 30.6)	29.7 (27.4, 32.2)	35.5 (32.3, 38.9)	44.1 (40.7, 47.5)	89.6 (86.5, 92.0)
Philippines	75.1 (73.1, 77.0)	-	14.3 (12.5, 16.4)	28.4 (26.0, 30.8)	55.7 (51.2, 60.1)
Rwanda	86.1 (84.7, 87.4)	14.9 (13.2, 16.7)	0.5 (0.3, 0.7)	23.6 (21.2, 26.2)	82.3 (78.1, 85.9)
Senegal	47.7 (44.6, 50.9)	59.5 (55.6, 63.3)	4.4 (3.2, 6.1)	23.6 (21.2, 26.2)	63.4 (54.9, 71.7)
Sierra Leone	66.7 (64.5, 68.8)	52.8 (50.7, 54.8)	5.5 (4.6, 6.5)	32.3 (30.3, 34.4)	65.0 (60.3, 69.5)
South Africa	71.3 (68.8, 73.7)	44.3 (40.7, 48.0)	7.7 (6.1, 9.8)	13.4 (11.3, 15.9)	36.3 (31.2, 41.7)
Tajikistan	67.2 (64.3, 69.9)	58.7 (56.0, 61.4)	7.4 (6.1, 9.0)	23.8 (21.5, 26.2)	79.0 (74.6, 82.9)
Tanzania	40.6 (38.4, 42.9)	40.3 (38.1, 42.5)	3.7 (2.7, 5.0)	24.0 (22.0, 26.3)	72.9 (68.1, 77.2)
Timor-Leste	63.6 (60.7, 66.4)	45.0 (42.4, 47.7)	22.9 (20.4, 25.6)	36.3 (33.8, 38.9)	71.6 (65.4, 77.1)
Togo	78.1 (75.8, 80.3)	45.6 (43.5, 47.8)	11.1 (9.7, 12.7)	34.7 (32.4, 37.0)	63.7 (57.7, 69.3)
Uganda	60.1 (58.4, 61.9)	48.0 (46.2, 49.8)	6.1 (5.4, 7.1)	28.3 (26.9, 29.9)	78.4 (74.5, 81.8)
Yemen	49.1 (47.2, 50.9)	54.8 (52.8, 56.7)	28.9 (26.9, 31.0)	43.3 (41.4, 45.2)	72.7 (68.3, 76.7)
Zambia	71.4 (69.5, 73.1)	39.6 (37.5, 41.7)	1.9 (1.4, 2.5)	18.5 (17.1, 20.0)	64.4 (58.8, 69.6)
Zimbabwe	65.2 (62.6, 67.8)	41.7 (39.3, 44.1)	11.8 (9.8, 14.2)	27.7 (25.3, 30.1)	68.1 (62.1, 73.6)

Tang K, *et al. BMJ Open* 2023; 13:e062387. doi: 10.1136/bmjopen-2022-062387

Supplementary Figure 1. Vitamin A supplementation (VAS) coverage between administrative regions for included countries in East and Southern Africa.

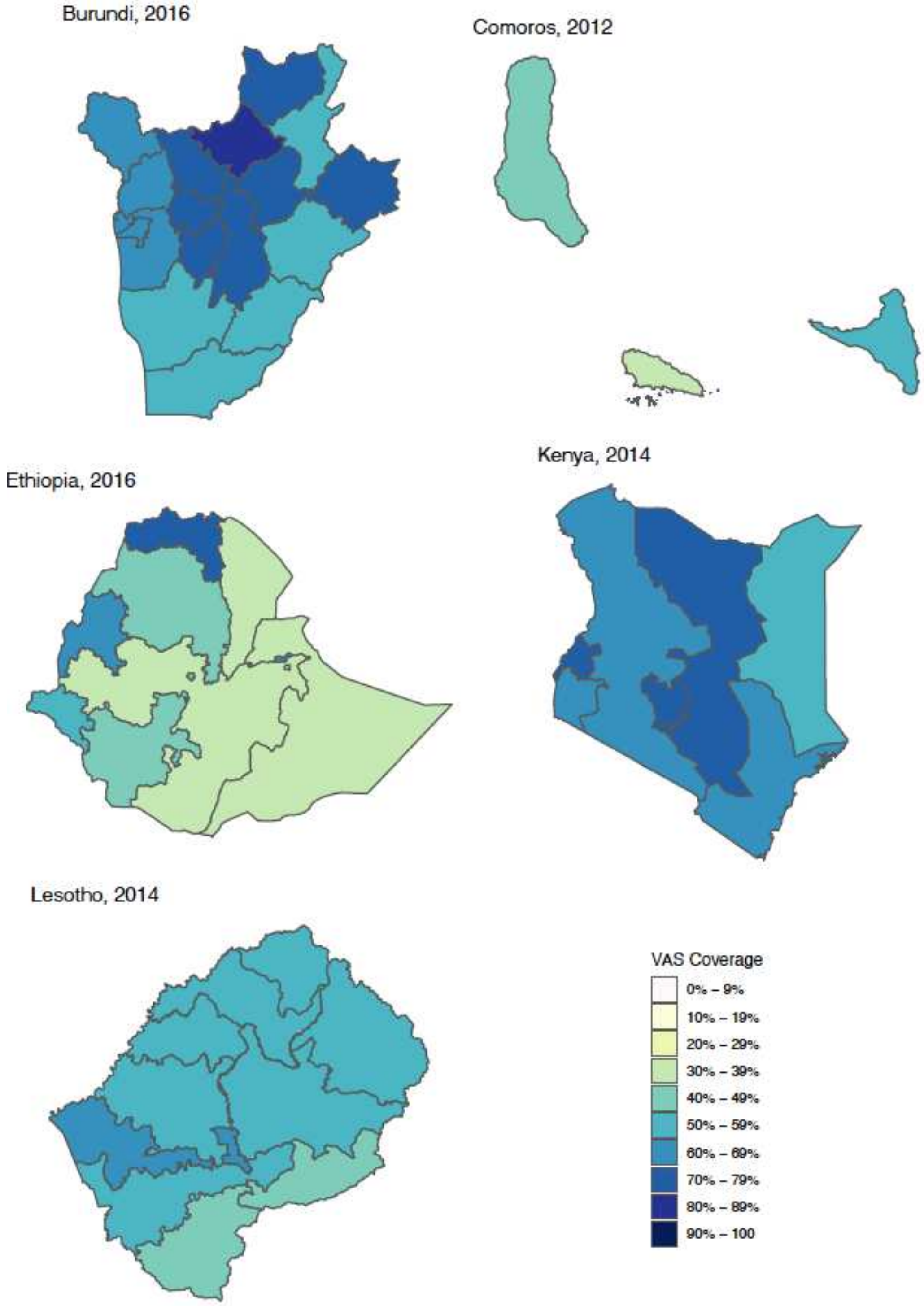

Tang K, *et al. BMJ Open* 2023; 13:e062387. doi: 10.1136/bmjopen-2022-062387

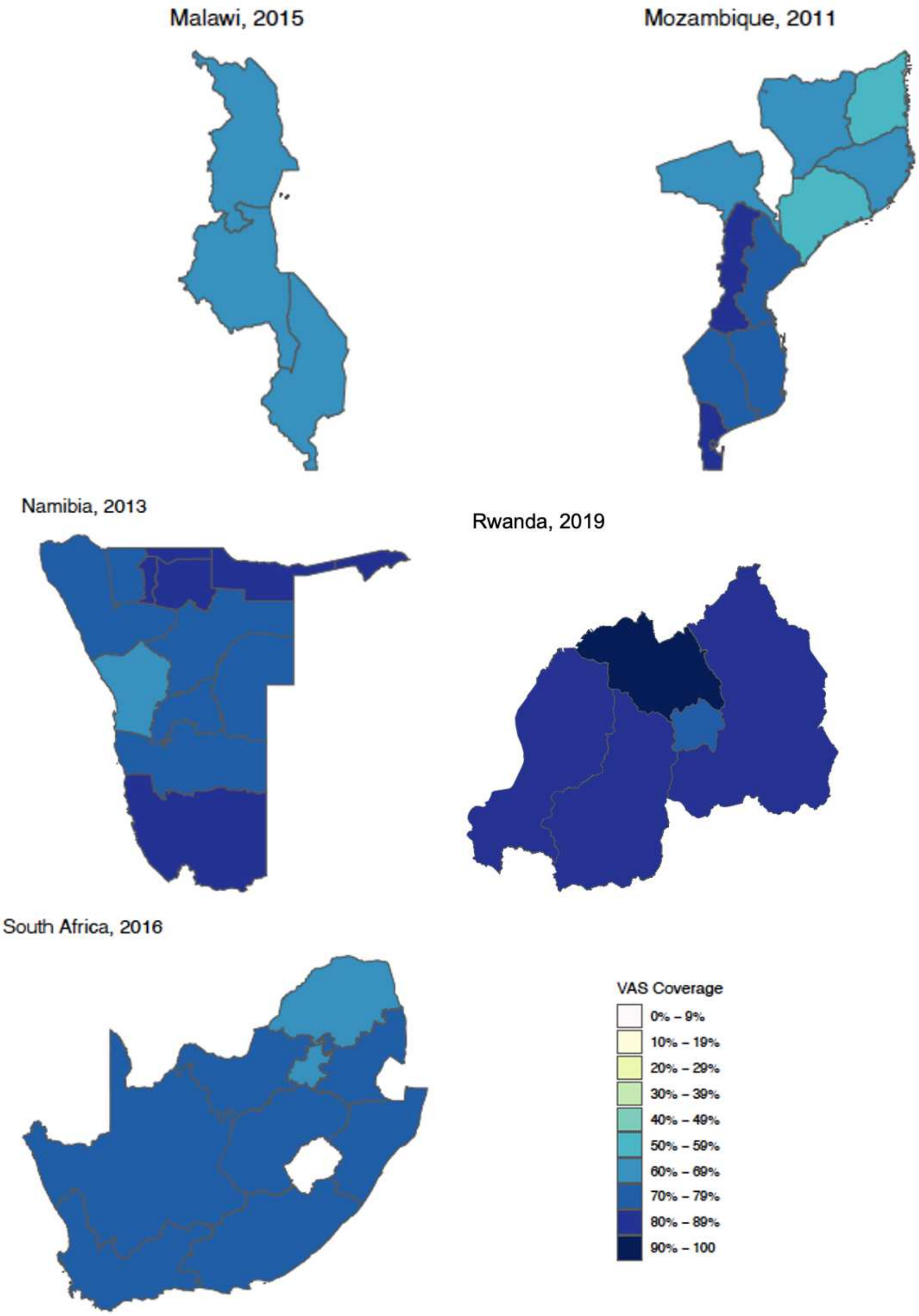
Malawi, 2015
Mozambique, 2011
Namibia, 2013
Rwanda, 2019
South Africa, 2016
VAS Coverage
0% – 9%
10% – 19%
20% – 29%
30% – 39%
40% – 49%
50% – 59%
60% – 69%
70% – 79%
80% – 89%
90% – 100

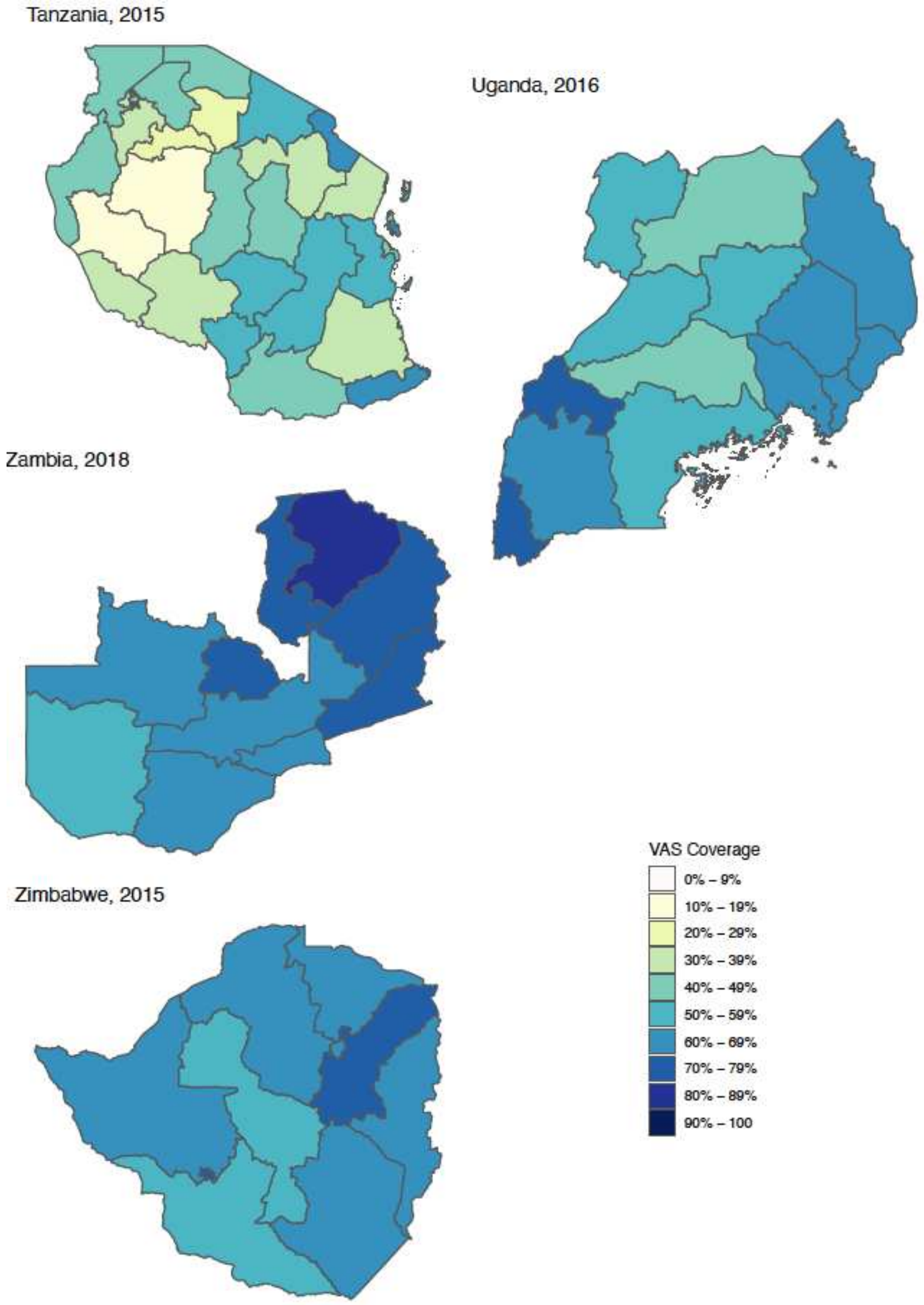
Tanzania, 2015
Uganda, 2016
Zambia, 2018
Zimbabwe, 2015
VAS Coverage
0% – 9%
10% – 19%
20% – 29%
30% – 39%
40% – 49%
50% – 59%
60% – 69%
70% – 79%
80% – 89%
90% – 100

Supplementary Figure 2. Vitamin A supplementation (VAS) coverage between administrative regions for included countries in West and Central Africa.

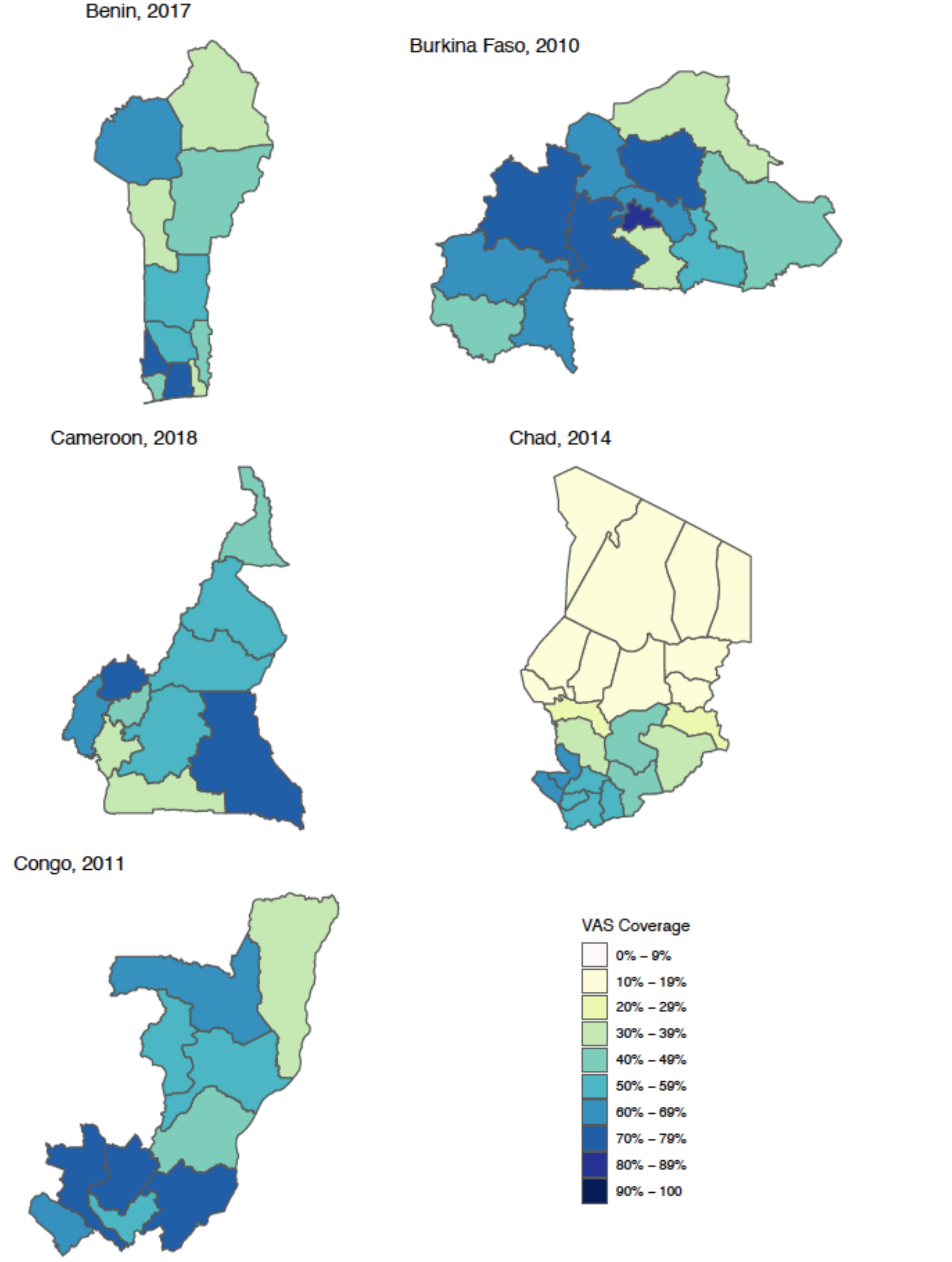

Tang K, *et al. BMJ Open* 2023; 13:e062387. doi: 10.1136/bmjopen-2022-062387

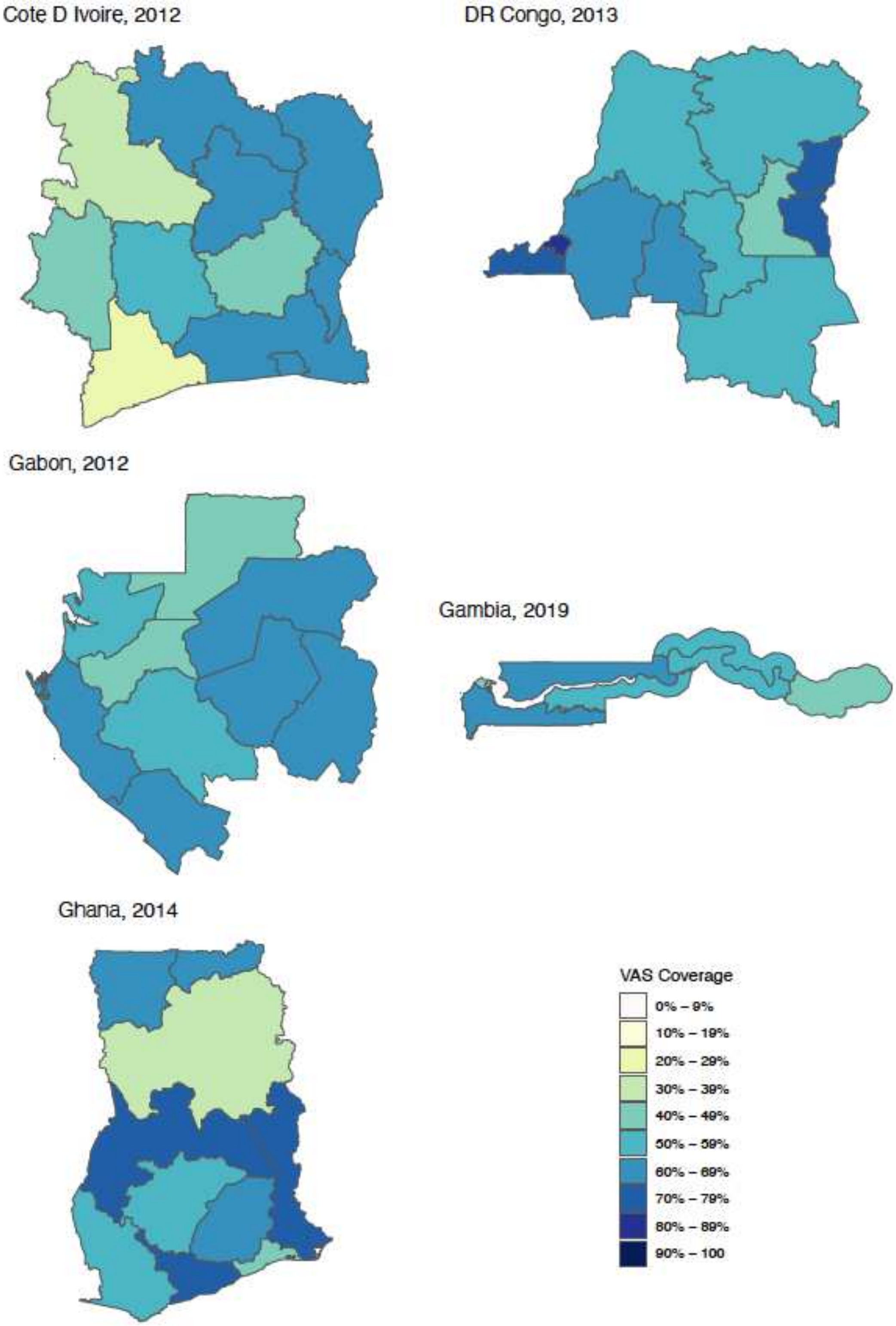
Cote D Ivoire, 2012
DR Congo, 2013
Gabon, 2012
Gambia, 2019
Ghana, 2014
VAS Coverage
0% – 9%
10% – 19%
20% – 29%
30% – 39%
40% – 49%
50% – 59%
60% – 69%
70% – 79%
80% – 89%
90% – 100

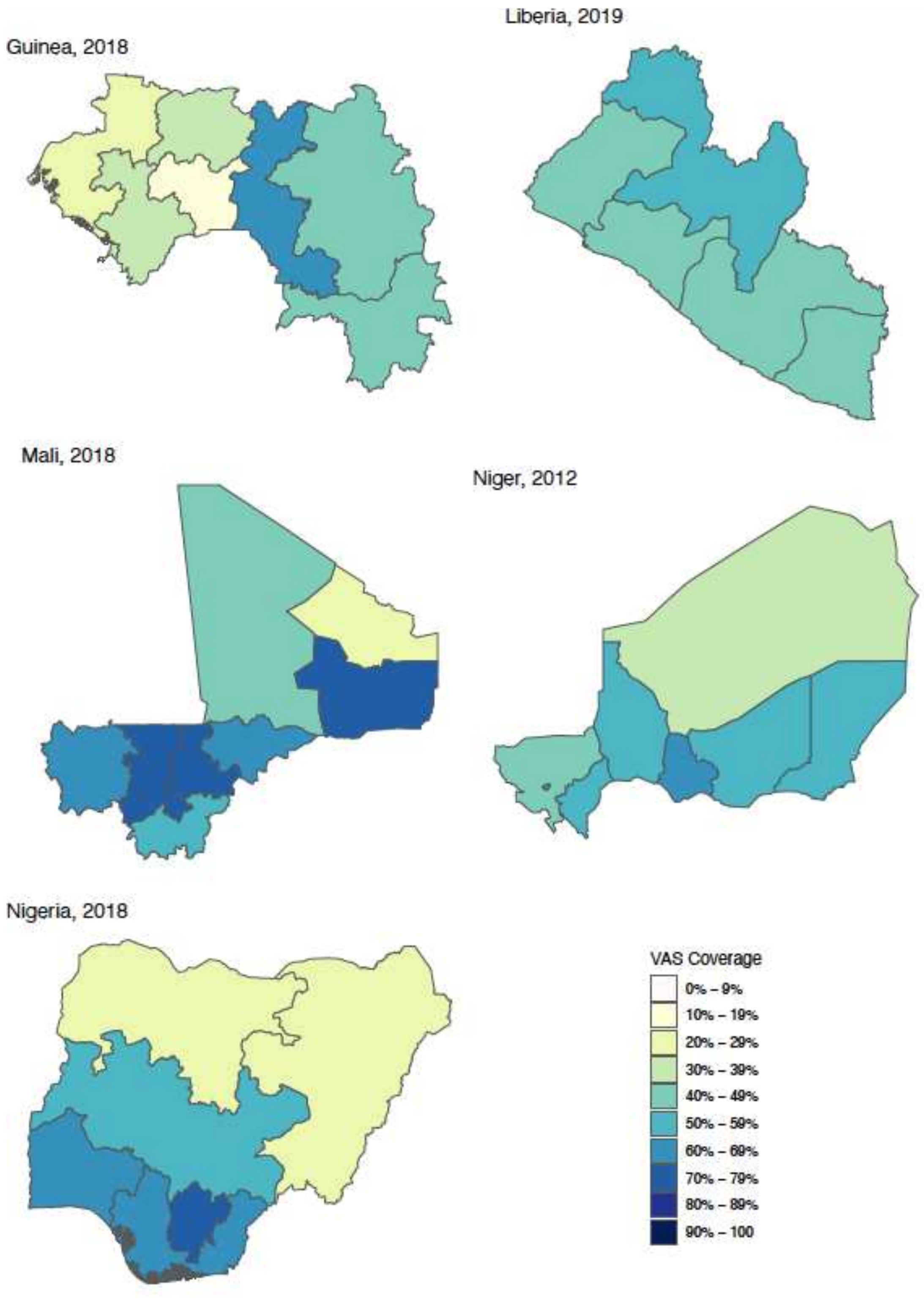
Guinea, 2018
Liberia, 2019
Mali, 2018
Niger, 2012
Nigeria, 2018
VAS Coverage
0% – 9%
10% – 19%
20% – 29%
30% – 39%
40% – 49%
50% – 59%
60% – 69%
70% – 79%
80% – 89%
90% – 100

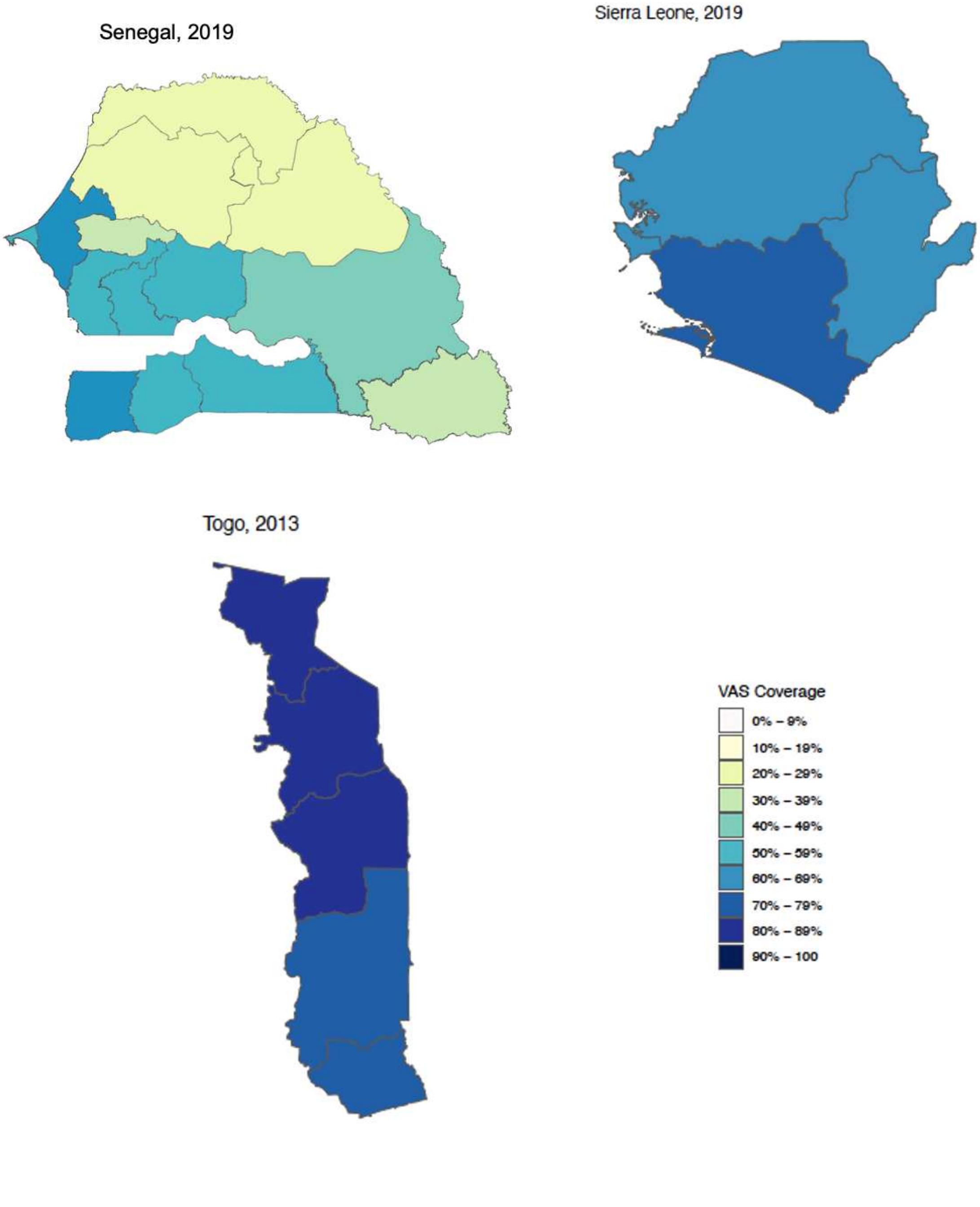
Senegal, 2019
Sierra Leone, 2019
Togo, 2013
VAS Coverage
0% – 9%
10% – 19%
20% – 29%
30% – 39%
40% – 49%
50% – 59%
60% – 69%
70% – 79%
80% – 89%
90% – 100

Supplementary Figure 3. Vitamin A supplementation (VAS) coverage between administrative regions for included countries in South Asia, East Asia, and the Western Pacific.

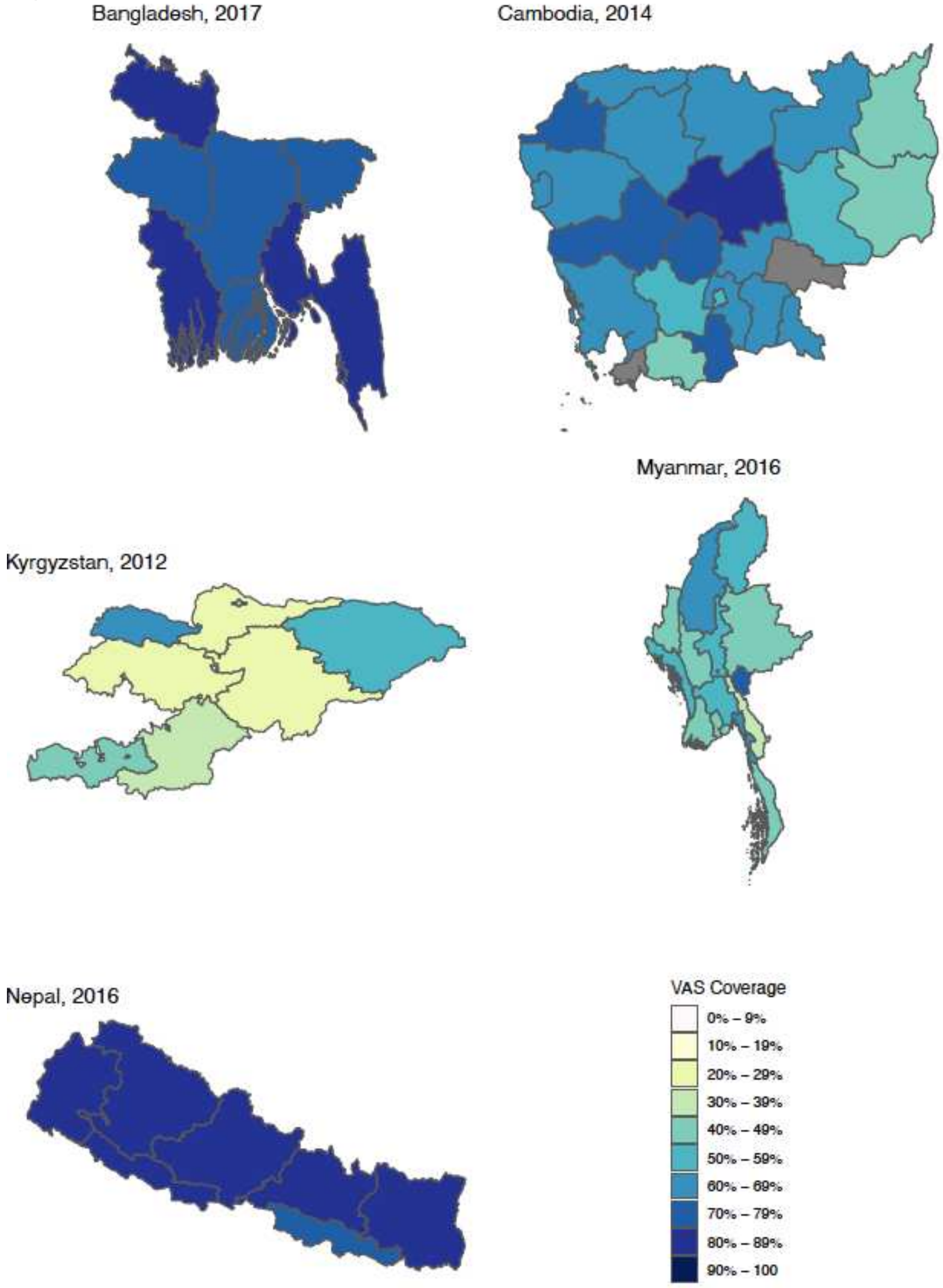

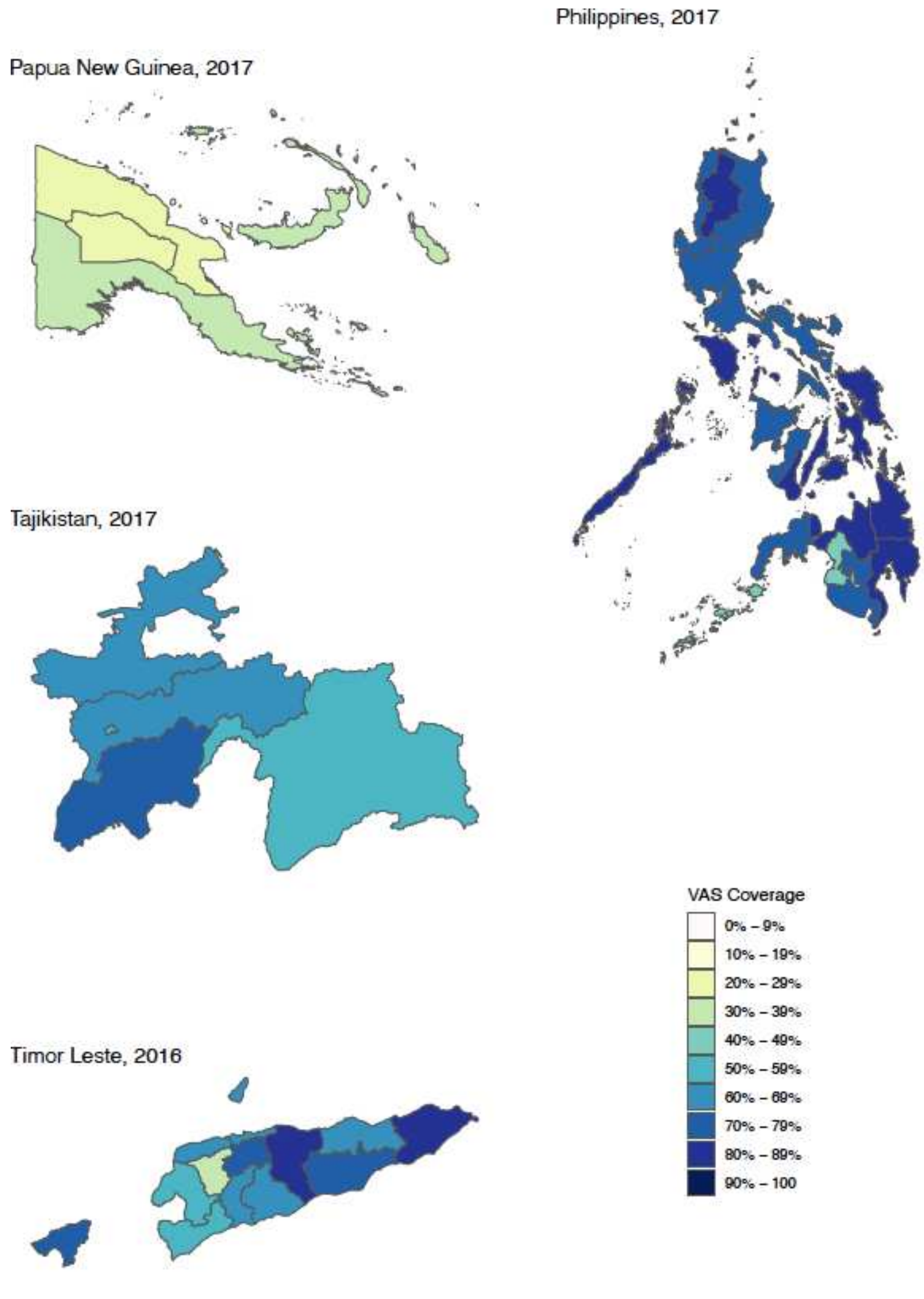
Philippines, 2017
Papua New Guinea, 2017
Tajikistan, 2017
Timor Leste, 2016
VAS Coverage
0% – 9%
10% – 19%
20% – 29%
30% – 39%
40% – 49%
50% – 59%
60% – 69%
70% – 79%
80% – 89%
90% – 100

India, 2015

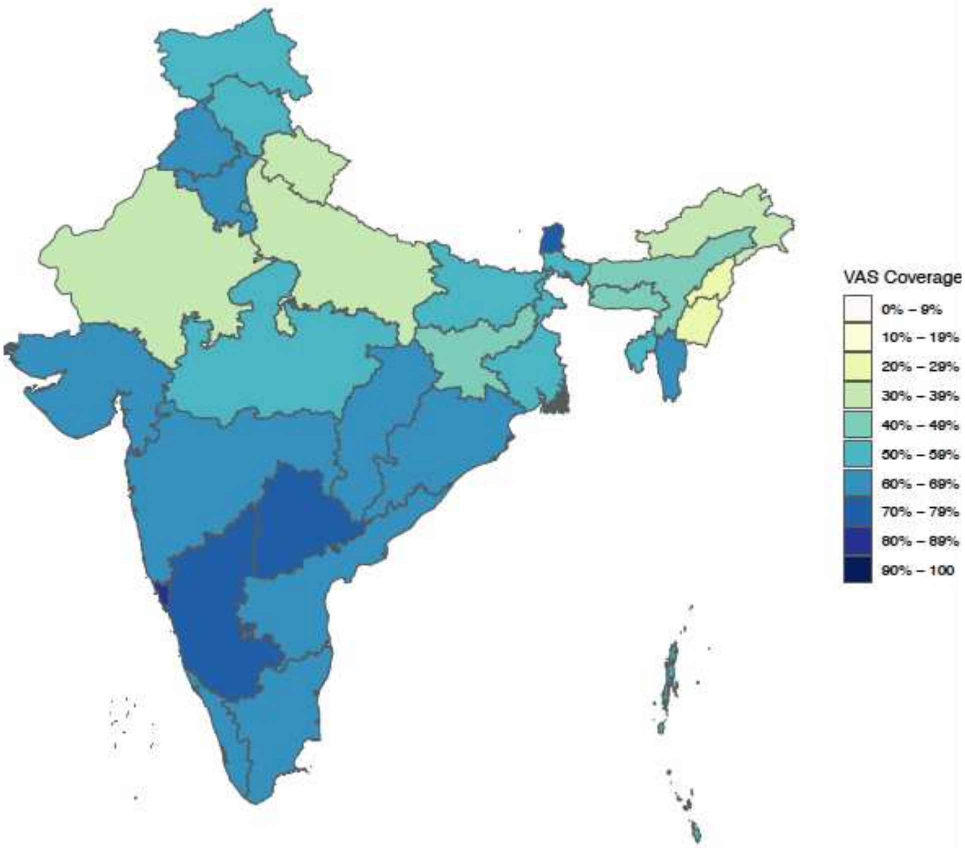

Indonesia, 2017

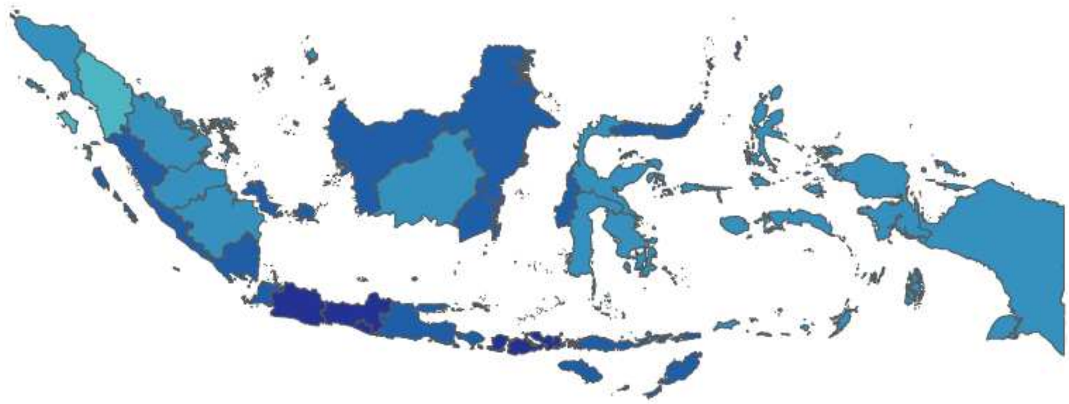

15

Supplementary Figure 4. Vitamin A supplementation (VAS) coverage between administrative regions for included countries in the Eastern Mediterranean Region.

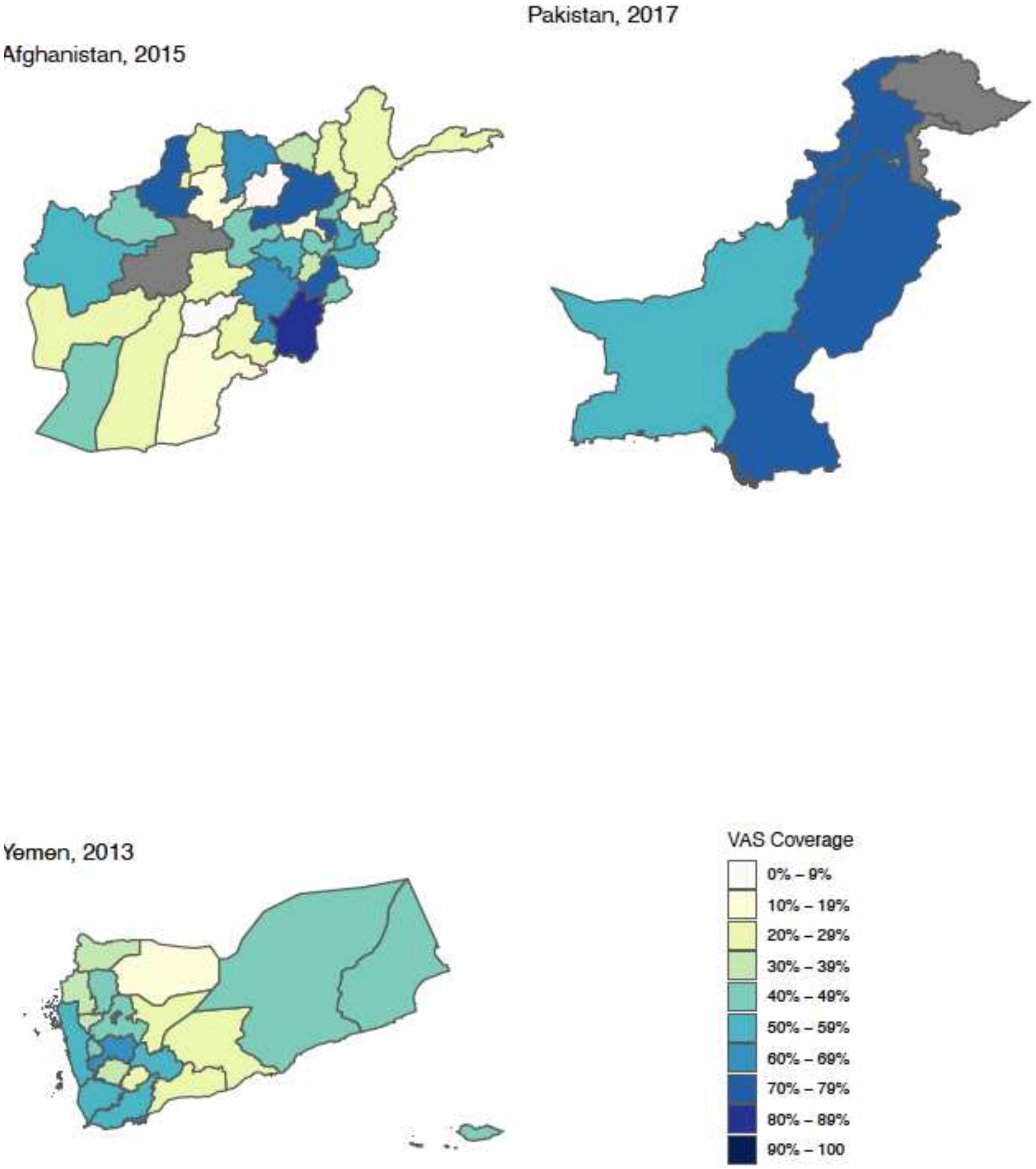

16

Supplementary Figure 5. Vitamin A supplementation (VAS) coverage between administrative regions for included countries in the Americas.

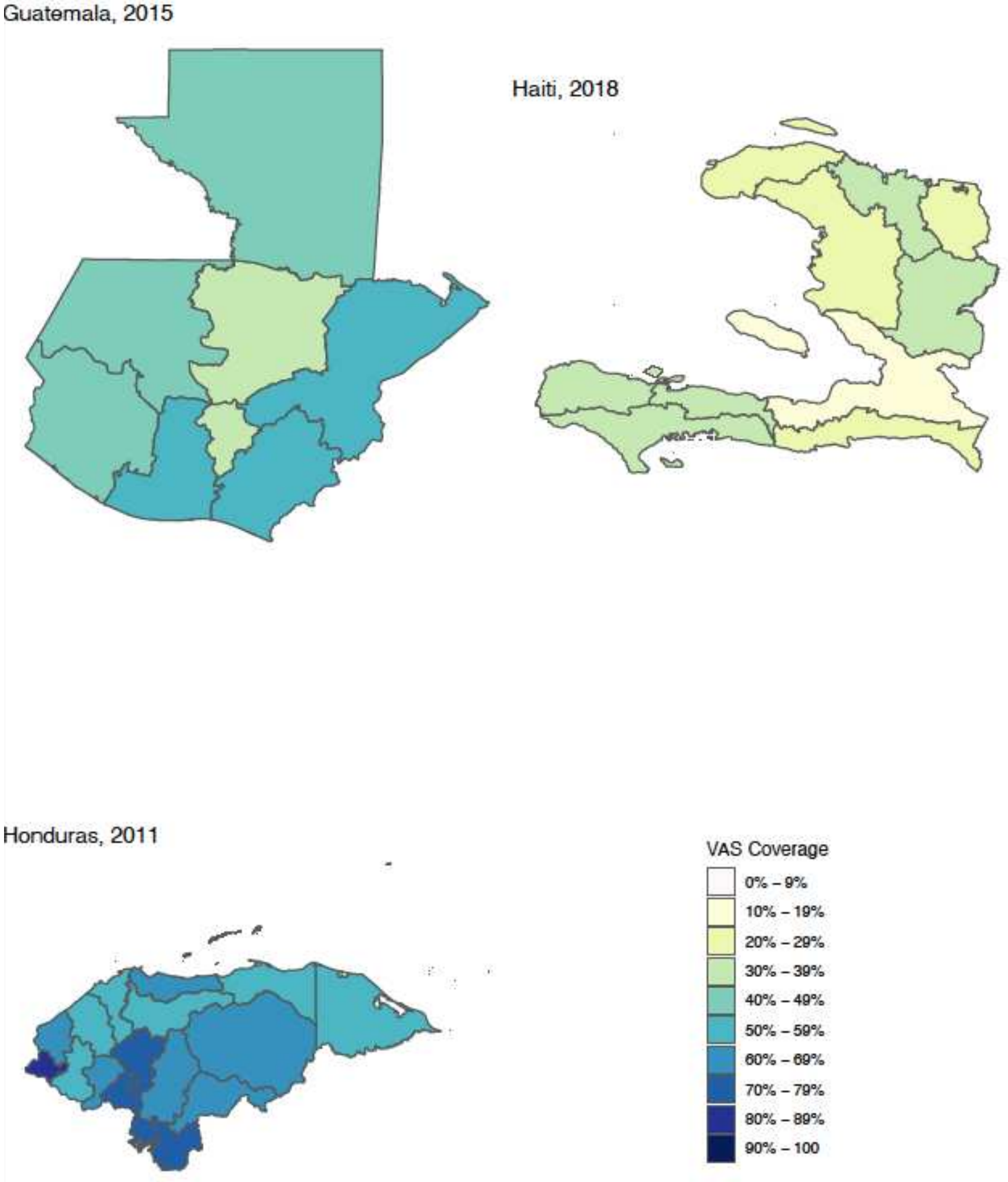

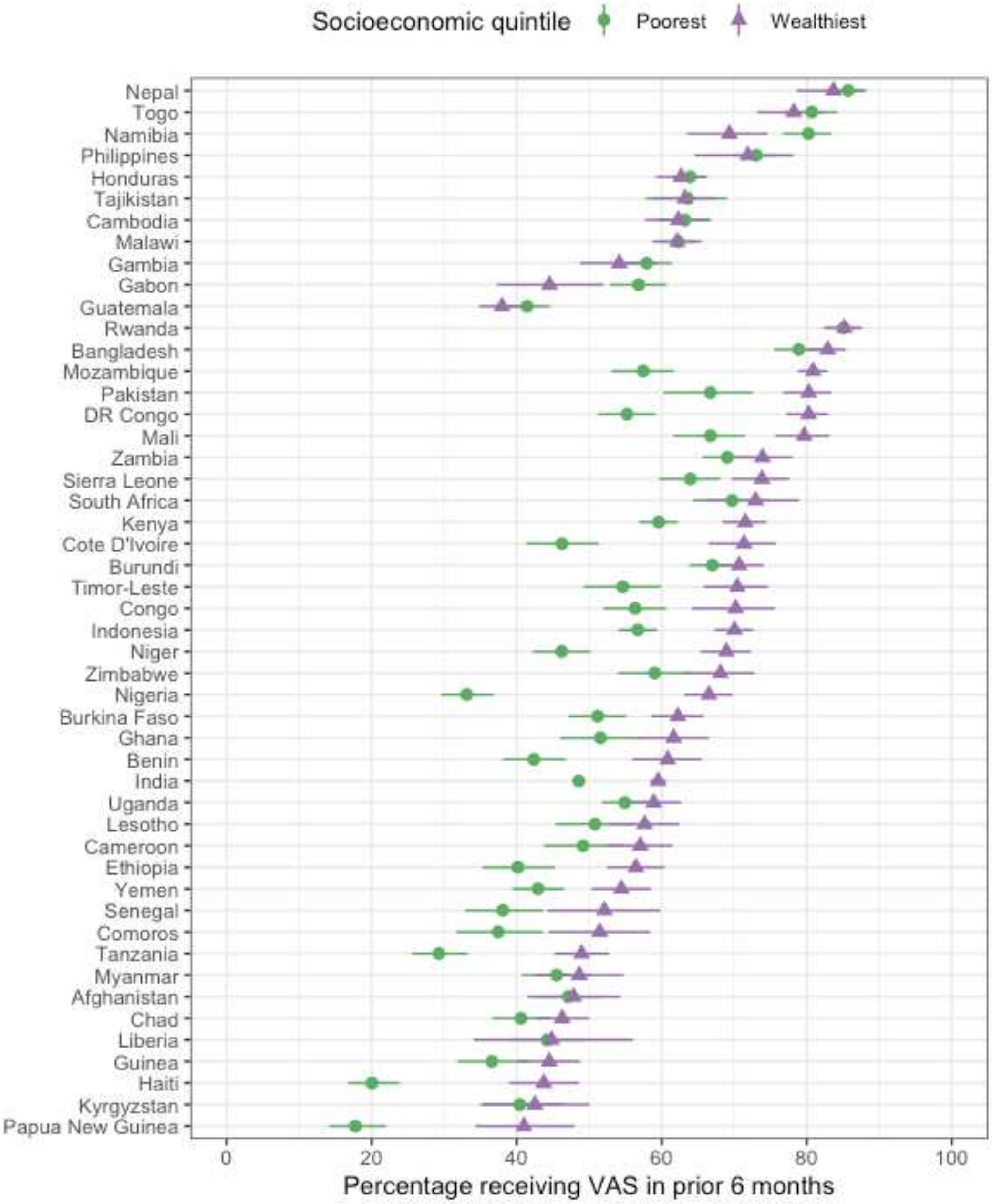

Supplementary Figure 6. Vitamin A supplementation (VAS) coverage among children from households of the poorest versus the richest socioeconomic quintiles.

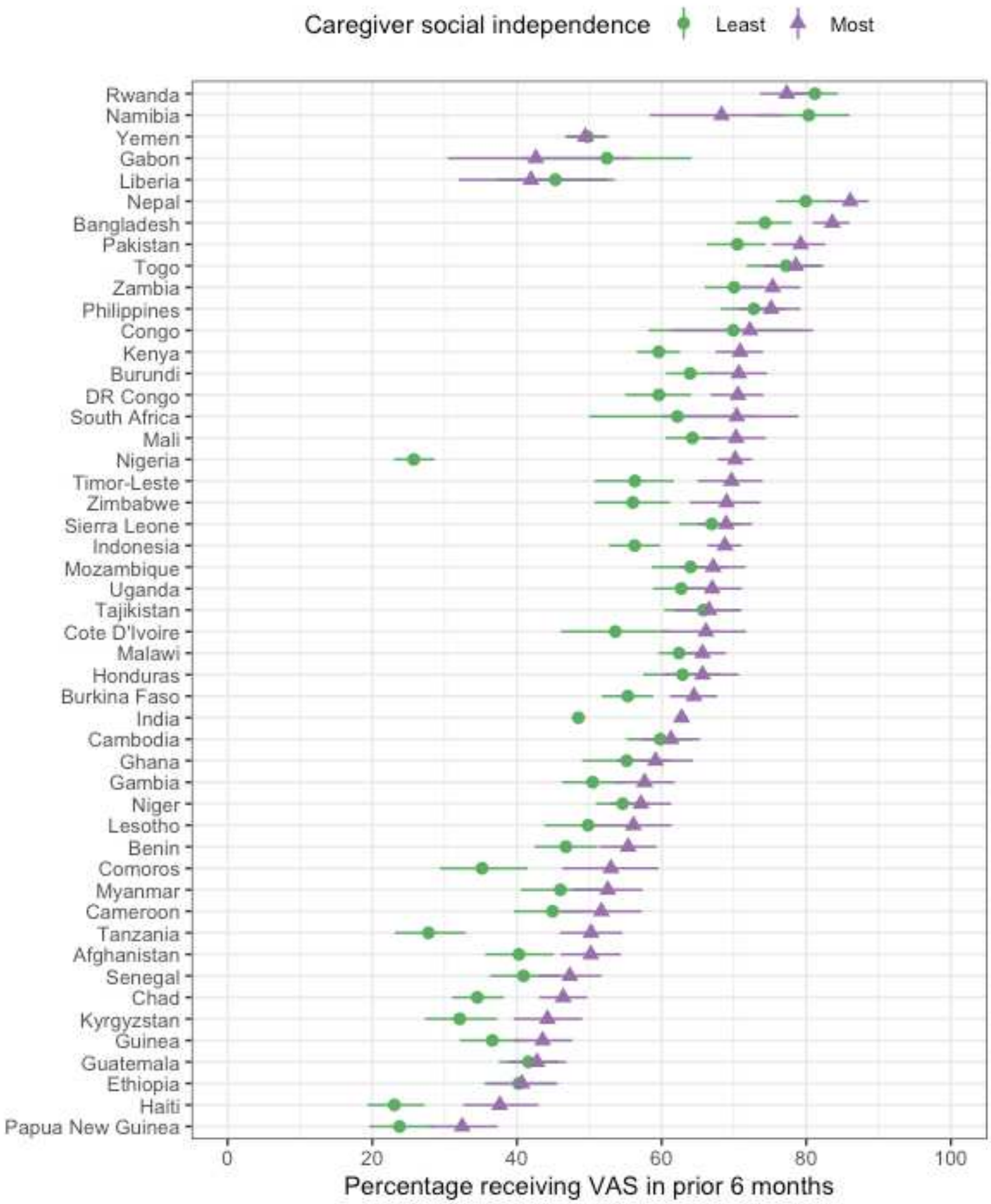

Supplementary Figure 7. Differences in vitamin A supplementation (VAS) coverage between children whose caregivers are the least versus the most socially independent in their country.

Tang K, *et al. BMJ Open* 2023; 13:e062387. doi: 10.1136/bmjopen-2022-062387

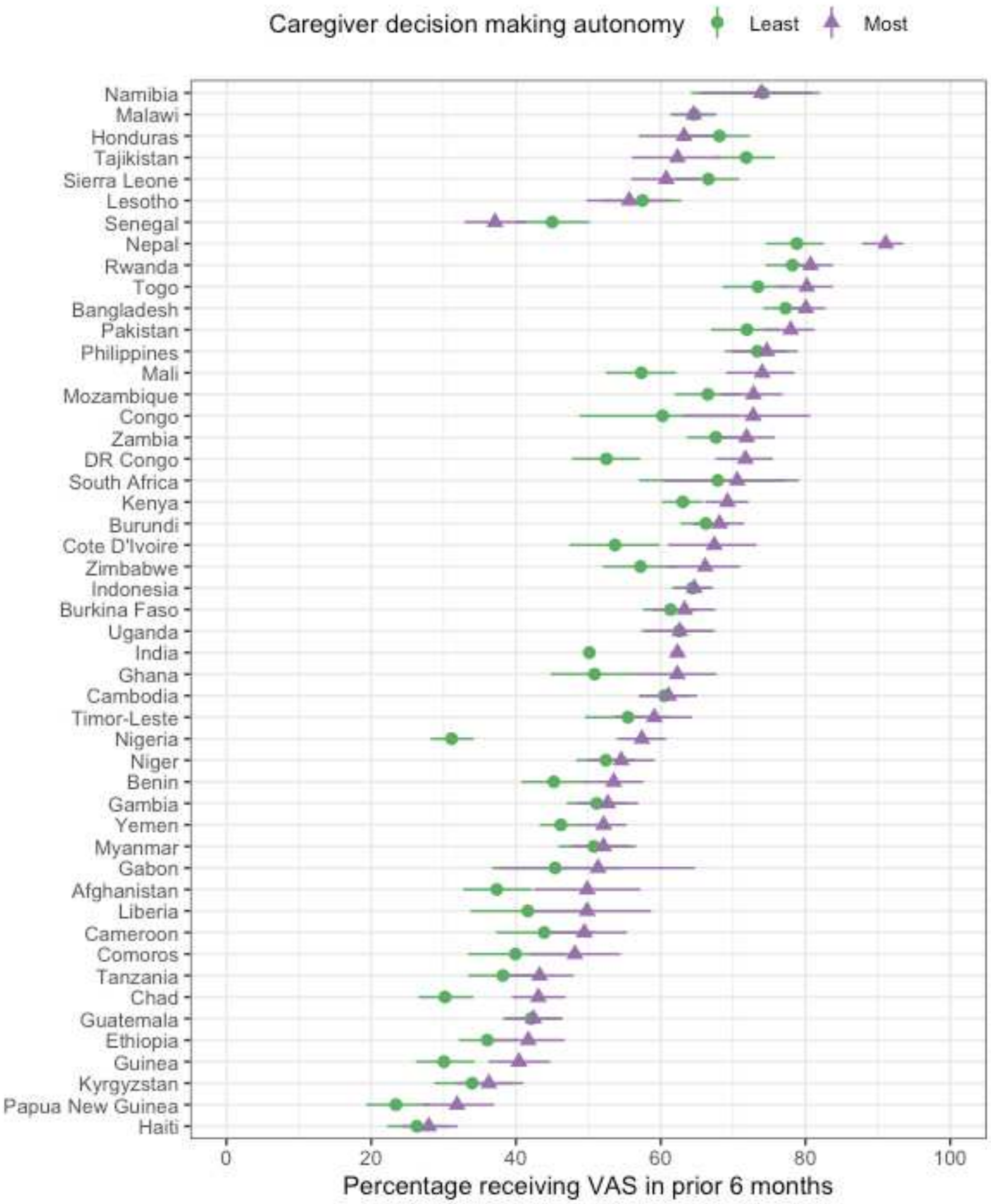

Supplementary Figure 8. Differences in vitamin A supplementation (VAS) coverage between children whose caregivers have the least versus the most decision-making autonomy in their country.

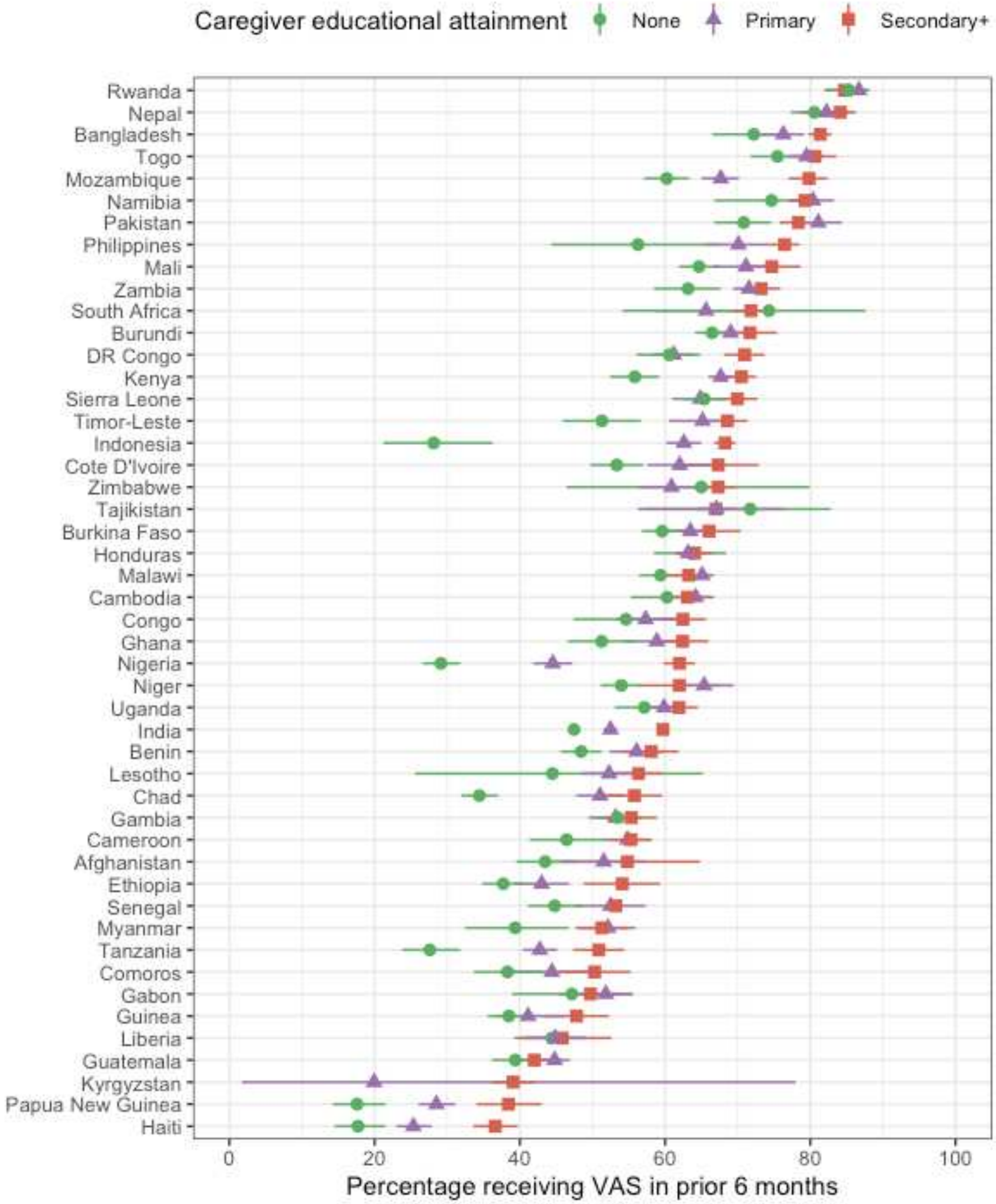

Supplementary Figure 9. Differences in vitamin A supplementation (VAS) coverage between children based on the educational attainment of the child's caregiver.

Tang K, *et al. BMJ Open* 2023; 13:e062387. doi: 10.1136/bmjopen-2022-062387

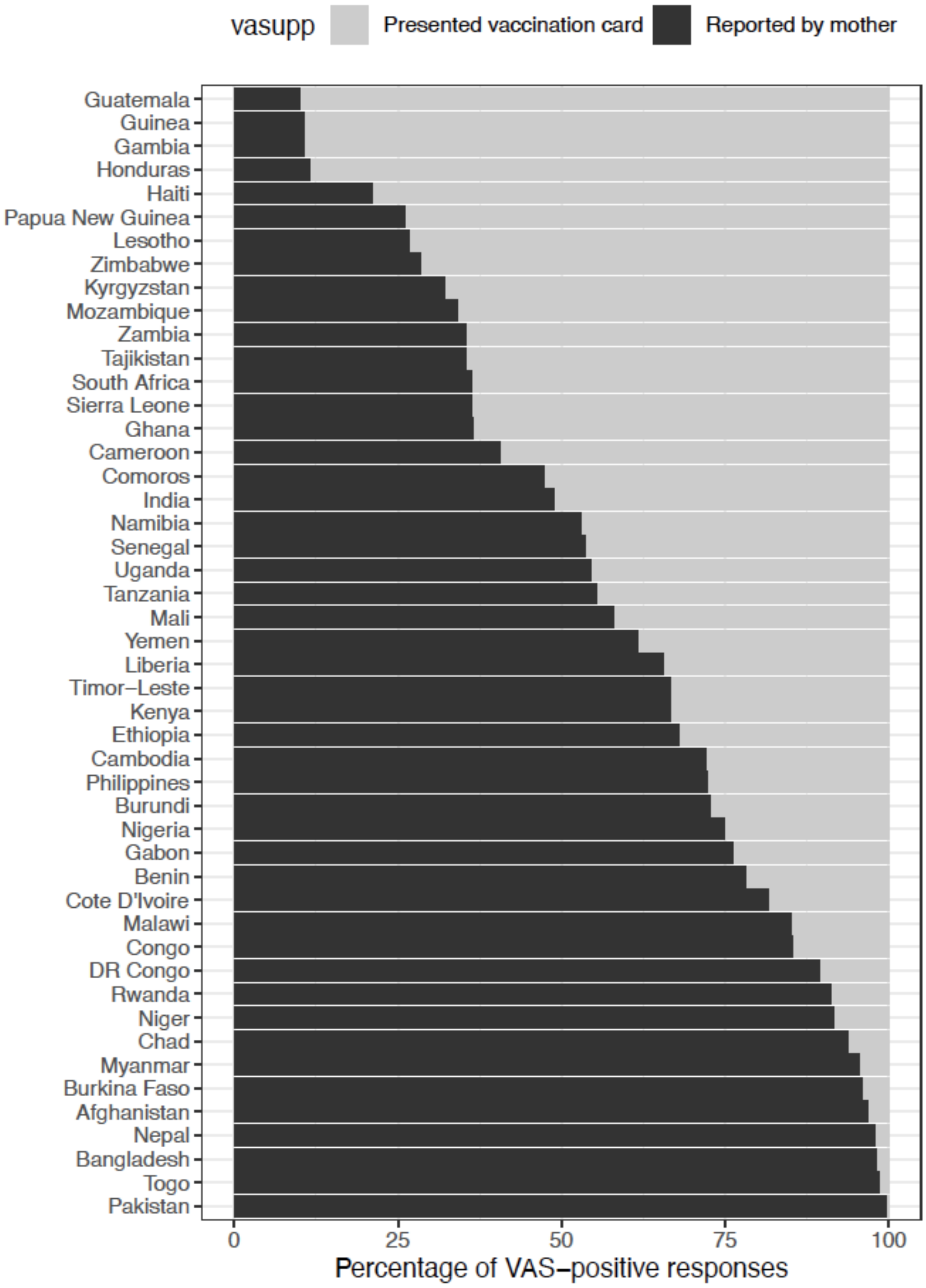

Supplementary Figure 10. Proportion of children who had received vitamin A supplementation (VAS) whose caregiver confirmed VAS reception with a vaccination card.
