# Supplementary material for: Evaluating equity dimensions of infant and child vitamin A supplementation programmes using Demographic and Health Surveys from 49 countries
Source: BMJ Open. 2023 Mar 14;13(3):e062387. doi: 10.1136/bmjopen-2022-062387 (PMC10016247; doi:10.1136/bmjopen-2022-062387)
Supplement: Author's manuscript [file bmjopen-2022-062387.draft_revisions.pdf]

BMJ Open is committed to open peer review. As part of this commitment we make the peer review history of every article we publish publicly available.

When an article is published we post the peer reviewers' comments and the authors' responses online. We also post the versions of the paper that were used during peer review. These are the versions that the peer review comments apply to.

The versions of the paper that follow are the versions that were submitted during the peer review process. They are not the versions of record or the final published versions. They should not be cited or distributed as the published version of this manuscript.

BMJ Open is an open access journal and the full, final, typeset and author-corrected version of record of the manuscript is available on our site with no access controls, subscription charges or pay-per-view fees (http://bmjopen.bmj.com).

If you have any questions on BMJ Open's open peer review process please email info.bmjopen@bmj.com

BMJ Open

Evaluating equity dimensions of infant and child vitamin A supplementation programs using Demographic and Health Surveys from 49 countries

Journal:	*BMJ Open*
Manuscript ID	bmjopen-2022-062387
Article Type:	Original research
Date Submitted by the Author:	01-Mar-2022

Complete List of Authors:	Tang, Kevin; London School of Hygiene & Tropical Medicine, Department of Population Health; UNICEF, Programme Division<br>Eilerts, Hallie; London School of Hygiene & Tropical Medicine, Department of Population Health<br>Imohe, Annette; UNICEF, Nutrition<br>Adams, Katherine; University of California Davis, Institute for Global Nutrition<br>Sandalinas, Fanny ; London School of Hygiene & Tropical Medicine, Department of Population Health<br>Joy, Edward; London School of Hygiene & Tropical Medicine, Department of Population Health<br>Moloney, Grainne; UNICEF, Programme Division<br>Hasman, Andreas; UNICEF, Nutrition

Keywords:	Health policy < HEALTH SERVICES ADMINISTRATION & MANAGEMENT, Nutrition < TROPICAL MEDICINE, PAEDIATRICS, Public health < INFECTIOUS DISEASES, Geographical mapping < TROPICAL MEDICINE, International health services < HEALTH SERVICES ADMINISTRATION & MANAGEMENT

SCHOLARONE™
Manuscripts

I, the Submitting Author has the right to grant and does grant on behalf of all authors of the Work (as defined in the below author licence), an exclusive licence and/or a non-exclusive licence for contributions from authors who are: i) UK Crown employees; ii) where BMJ has agreed a CC-BY licence shall apply, and/or iii) in accordance with the terms applicable for US Federal Government officers or employees acting as part of their official duties; on a worldwide, perpetual, irrevocable, royalty-free basis to BMJ Publishing Group Ltd ("BMJ") its licensees and where the relevant Journal is co-owned by BMJ to the co-owners of the Journal, to publish the Work in this journal and any other BMJ products and to exploit all rights, as set out in our licence.

The Submitting Author accepts and understands that any supply made under these terms is made by BMJ to the Submitting Author unless you are acting as an employee on behalf of your employer or a postgraduate student of an affiliated institution which is paying any applicable article publishing charge ("APC") for Open Access articles. Where the Submitting Author wishes to make the Work available on an Open Access basis (and intends to pay the relevant APC), the terms of reuse of such Open Access shall be governed by a Creative Commons licence – details of these licences and which Creative Commons licence will apply to this Work are set out in our licence referred to above.

Other than as permitted in any relevant BMJ Author's Self Archiving Policies, I confirm this Work has not been accepted for publication elsewhere, is not being considered for publication elsewhere and does not duplicate material already published. I confirm all authors consent to publication of this Work and authorise the granting of this licence.

For peer review only

Evaluating equity dimensions of infant and child vitamin A supplementation programs using Demographic and Health Surveys from 49 countries

Author names: Kevin Tang[1 2], Hallie Eilerts[1], Annette Imohe[2], Katherine P Adams[3], Fanny Sandalinas[1], Edward JM Joy[1], Grainne Moloney[2], Andreas Hasman[2]

Author affiliations:

[1] Department of Population Health, London School of Hygiene & Tropical Medicine, London, WC1E 7HT, United Kingdom

[2] UNICEF Programme Division, 3 United Nations Plaza, New York, NY 10017, United States

[3] Institute for Global Nutrition, University of California, Davis, One Shield Avenue, Davis, CA, 95616, United States

*Corresponding Author

Kevin Tang

Department of Population Health

London School of Hygiene & Tropical Medicine

Keppel Street

London, WC1E 7HT, United Kingdom

P: (+44) 20 7636 8636

F: (+44) 20 7436 5389

Email: kevin.tang1@lshtm.ac.uk

For peer review only

ABSTRACT (286/300 words max)

Objectives: Vitamin A deficiency affects an estimated 29% of all children under five years of age in low- and lower middle-income countries, contributing to child mortality and exacerbating severity of infections. Bi-annual vitamin A supplementation (VAS) for children aged 6 – 59 months can be a low-cost intervention to meet vitamin A needs. This study aimed to present a framework for evaluating the equity dimensions of national VAS programs according to determinants known to affect child nutrition and assist programming by highlighting geographic variation in coverage.

Methods: We used open-source data from the Demographic and Health Survey for 49 countries to identify differences in VAS coverage between sub-populations characterized by various immediate, underlying, and enabling determinants of vitamin A status and geographically. This included recent consumption of vitamin A-rich foods, access to health systems and services, administrative region of the country, place of residence (rural vs urban), socioeconomic position, caregiver educational attainment, and caregiver empowerment.

Results: Children who did not recently consume vitamin A-rich foods and who had poorer access to health systems and services were less likely to receive VAS in most countries despite potentially having a greater vitamin A need. Differences in coverage were also observed when disaggregated by administrative regions (88% of countries) and urban vs rural residence (35% of countries). Differences in vitamin A coverage between sub-populations characterized by other determinants of vitamin A status varied considerably between countries.

Conclusion: Sub-population differences in VAS coverage characterized by various determinants of vitamin A status suggest that VAS programs may not be operating equitably in many

For peer review only

countries. National VAS programs can use this analysis as a framework to use open-source data to improve targeting and prioritization of children who may likely be most in need.

For peer review only

KEY QUESTIONS

What is already known on this topic?

- Vitamin A supplementation (VAS) programs for children aged 6 – 59 months can reduce risks of vitamin A deficiency, alleviating related mortality and exacerbated disease burden.
- VAS programs can operate alongside existing community-based health service delivery programs, but there are challenges accessing hard-to-reach populations who may have greater risks for vitamin A deficiency.
- VAS reception is included in the Demographic and Health Survey (DHS), but this data is often underutilized when informing decision making.

What does this study add?

- Children who likely have limited access to vitamin A-rich foods are less likely to receive VAS in most countries, and those who have impaired access to healthcare systems and services are also less likely to receive VAS in several countries.
- VAS coverage frequently varied by sub-populations within countries, defined both geographically and by other enabling determinants affecting vitamin A status.

How might this study affect research, practice, or policy?

- DHS data can be useful to inform national governments on how to orient their VAS delivery strategy to target population who potentially are most in need of vitamin A supplementation.

For peer review only

INTRODUCTION

The UNICEF Nutrition Strategy 2020 – 2030 commits to a goal of protecting and promoting diets, services, and practices that support optimal nutrition, growth, and development for all children to end child malnutrition in all its forms [1]. Central to achieving this goal requires recognizing the interaction between five core systems (food, health, water & sanitation, education, social protection) to improve nutrition outcomes. However, a strategy focused on working within established systems may exacerbate unequal nutrition outcomes rooted in deeper inequities that arise from already inequitable systems [2]. Therefore, when defining policies to ensure the most vulnerable have access to services, regular review of coverage must be implemented to allow for reorientation as needed.

Vitamin A deficiency affects an estimated 29% of all children under five years of age in low- and middle-income countries [3], contributing to child mortality and disease burden through direct clinical manifestation (e.g., xeropthalmia) and susceptibility to and exacerbated adverse outcomes from infection (e.g., measles, diarrhea) [4]. For children with vitamin A deficiency or who have an increased risk of mortality, high-dose vitamin A supplementation (VAS) can be a low-cost intervention to meet vitamin A needs when delivered using infrastructure from existing community-based delivery programs [5]. Following an exhaustive review of the current evidence exploring the effect of VAS in preventing morbidity and mortality in infants and children [4,6], in 2011 the World Health Organization (WHO) confirmed a recommendation for infants and children 6 – 59 months of age to receive a dose of VAS every four to six months in contexts where vitamin A deficiency is a public health problem [7]. Although the past two

For peer review only

decades have seen increases in global VAS coverage [8], stagnating VAS coverage in recent years brings into question whether current universal delivery strategies are consistently missing hard-to-reach children and whether a targeted approach should be considered [9]. This is particularly the case as VAS programs move away from delivery in campaigns towards routine delivery [8].

It is recommended that the delivery of VAS is integrated into community-based health service delivery programs [10]. In many countries, community-based delivery programs, such as the national Essential Programme on Immunization (EPI), offer the most consistent contacts between the youngest children and the health system, thus creating a platform for the large-scale administration of VAS. However, in contrast to VAS administration protocols, childhood immunization programs typically benefit only children up to one year of age when the last vaccine dose is administered, and few countries have a contact point for immunization at six months. Moreover, multi-country analyses of diphtheria-tetanus-pertussis (DTP) and measles containing vaccines (MCV) identified gaps in program coverage between geographic and socioeconomic subpopulations [11,12]. All together, these differences suggest that the services these community-based delivery programs provide are not equitably accessible to children with the greatest needs and may affect the VAS programs that depend on these services for delivery.

Global guidance for monitoring VAS programs relies on “two-dose coverage” as the primary metric for estimating global progress towards achieving universal VAS coverage [8,13].

For peer review only

In this context, “coverage” is estimated as the aggregated number of VAS doses administered in a country over a six-month period (also referred to as semester) divided by the estimated number of children ages 6 – 59 months in that country for that specific semester. “Two-dose coverage” is thus established on an annual basis as the semester in a given year with lower coverage. While two-dose coverage increases the feasibility of collating national VAS program data bi-annually, the metric is limited in identifying differences in coverage among sub-populations within a country. This restricts national VAS programs from evaluating whether they are missing the children, particularly those with the greatest needs.

There is a significant information gap in evaluating the equity dimensions of nutrition interventions, or the extent to which a community-based nutrition program is reaching the children most in need [2]. Nationally representative household surveys, such as the Demographic and Health Surveys (DHS), can potentially provide insight into the equity dimensions of program delivery. Since 1985, the DHS program has conducted nationally-representative cross-sectional surveys in over 90 predominantly low-middle income countries, producing detailed, cross-sectional data on a variety of indicators describing demographics, health, economics, and social welfare [14]. DHS questionnaires and variable nomenclature are consistent between all countries in the DHS program, facilitating rapid analysis across multiple countries and survey years. DHS microdata enable the exploration of a variety of indicators to measure population health outcomes and the mechanisms influencing them in great depth.

For peer review only

DHS collect detailed information on the reception of VAS by infants and children, however using such data to inform national VAS programs is not straightforward. The aim of this study was to present a framework for evaluating the equity dimensions of national VAS programs according to determinants known to affect child nutrition and geographically using DHS data from multiple countries.

For peer review only

For peer review only - http://bmjopen.bmj.com/site/about/guidelines.xhtml

METHODS

Demographic and Health Surveys

We used the most recent DHS data from 49 countries in which a complete survey was conducted since 2010. The analysis depended on data from the Woman's Questionnaire, which contains information on the survival status of the children born to the respondent, and more detailed information on children born in the last five years, such as vaccination history, breastfeeding practice, recent illness, and anthropometry.

Sampling Strategy & Study Population

The DHS protocol employs a two-stage sampling procedure [15]. In the first stage, enumeration areas are defined geographically (stratified by urban/rural residence and administrative region) using the country's most recent population census to establish the survey's primary sampling unit. In the second stage, systematic sampling identifies 20 – 30 households for inclusion, where selected households are visited by trained interviewers who administer the Woman's Questionnaire to women of reproductive age (15-49 years).

Our analysis included all children aged 6 – 59 months with available VAS history data. VAS program participation is probed in the DHS questionnaire's *vaccination history section* by asking caregivers of children whether her child (or children) aged 6 – 59 months has (or have) received a vitamin A dose in the last six months [16]. If possible, caregivers are asked to present proof of VAS reception using a vaccination history card, and if not available, are asked to recall VAS administration from memory after probing the caregivers to describe post-natal care activities

For peer review only

in which her and each child have participated [17]. The primary outcome of this study was defined as the percentage of children receiving VAS in the prior six months, or more simply, “VAS coverage”.

Equity and Geographic Covariates

We stratified the primary outcome by several covariates describing various dimensions of equity, which were selected in accordance with the 2020 UNICEF Conceptual Framework on the Determinants of Maternal and Child Nutrition [1]. The framework used in this analysis was developed to guide nutrition programming by outlining three levels of determinants (i.e., immediate, underlying, enabling) that contribute to preventing malnutrition in all its forms. By using this framework to guide covariate selection, this study recognizes the levels and interconnectedness of various determinants affecting vitamin A status to define the underlying systems and processes that affect children with the greatest vitamin A needs. Geographic covariates were also selected as targeting geographically defined populations is more operationally feasible.

Covariates used to represent immediate and underlying child nutrition determinants included recent consumption of vitamin A-rich foods and access to healthcare systems and services. Recent consumption of vitamin A-rich foods from the DHS questionnaire is only collected for children aged 6 – 23 months, so this indicator refers predominantly to children who are within the age range where breastfeeding is recommended [18]. Access to healthcare systems and services is measured using the proxy variable of vaccine reception. The first dose of the DTP

For peer review only

vaccine (administered at six weeks of age) is one of the first vaccines given to an infant, where children who do not receive it are expected to have the lowest access to healthcare systems and services. Reception of the first dose of MCV (administered at nine months of age) is expected to be delivered via similar platforms to VAS through both routine provisions and campaigns. Covariates serving as indicators for enabling child nutrition determinants included socioeconomic position, the caregiver's educational attainment, and two women's empowerment dimensions calculated according to the Survey-based Women's Empowerment (SWPER) index [19], which measure the caregiver's social independence and decision-making autonomy. Geographic covariates included urban vs rural residence and administrative regions specific to each country. Descriptions of all covariates are available in Table 1.

For peer review only

Table 1. Description of study covariates used to represent immediate, underlying, and enabling determinants of child nutrition and geography

Determinant	Type	Coding details
Recent consumption of vitamin A-rich foods	Immediate/ Underlying	Whether the following types of food were reported being consumed by the child (aged 6-23 months) in the 24 hours prior to the interview: eggs, any meat, fish, orange-fleshed pumpkin/carrot/squash/sweet potato, vitamin A-rich fruit (mango, papaya), liver/heart/organ meat, dark-leafy-green vegetables
Access to healthcare services & systems (DTP1 vaccine)	Immediate/ Underlying	Whether the child received the first dose of the diphtheria, tetanus, and pertussis (DTP1) vaccine, administered at 6 weeks of age per international guidelines
Access to healthcare services & systems (MCV1 vaccine)	Immediate/ Underlying	Whether the child received the first dose of the measles containing vaccine (MCV1), administered at 9 months of age per international guidelines
Administrative region	Geography	Administrative region the household is located, defined by the sub-national reporting area (provinces or groups of provinces) as defined by the DHS recode
Place of residence	Geography	Whether household is in an urban or rural location
Socioeconomic position	Enabling	Quintile of household wealth defined using a principal component analysis score comprised of household living conditions and durable assets (Poorest = 1st quintile, Wealthiest = 5th quintile)
Caregiver's social independence	Enabling	Women's empowerment indicator calculated as quintiles of SWPER index. Social independence domain included data related to education, frequency of reading newspapers/magazines, and age at first childbirth and at first cohabitation (includes children with partnered caregivers) (Least socially independent = 1st quintile, Most socially independent = 5th quintile)
Caregiver's decision-making autonomy	Enabling	Women's empowerment indicator calculated as quintiles of SWPER index. Decision-making autonomy domain included questions about involvement in household decisions and whether the respondent worked in the past 12 months (includes children with partnered caregivers) (Least decision-making autonomy = 1st quintile, Most decision-making autonomy = 5th quintile)
Caregiver's educational attainment	Enabling	Highest educational attainment of caregiver (None, primary school, secondary school or higher)

For peer review only - http://bmjopen.bmj.com/site/about/guidelines.xhtml

Statistical Analysis

Percentage of children receiving VAS in the prior six months was stratified by each equity covariate independently for every included country. All estimates were adjusted using population sample weights provided by the DHS Program [20]. Percentages of children receiving VAS in each stratum in the prior six months were presented with 95% confidence intervals (95%CI), where statistically significant differences in VAS coverage were identified as those with non-overlapping 95%CIs. VAS coverage by administrative region was mapped using shapefiles downloaded from GADM (version 3.6).

Data analysis was conducted in RStudio (version 3.6.1, the R Foundation for Statistical Computing). Data was accessed through the DHS program's application programming interface (API) on 1 February 2022 via the functions available on the *rdhs* package [21]. Survey weight adjustments and statistical analyses were conducted using the functions available on the *survey* and *srvyr* packages [22] and additional data cleaning and management used a variety of functions from the *tidyverse* package [23].

Patient and Public Involvement

As a secondary analysis of publicly available, de-identified data collected as part of the DHS, patients and the public that were included as part of the study population were not involved in the design of this study.

For peer review only

RESULTS

Summary of included surveys & populations

In total, the birth history records of 1,464,997 women, corresponding to 608,578 children 6-59 months, were included in this study. For the survey question asking about VAS reception within the prior six month, the rate of response was >90% for all countries included. A summary of all included countries and the DHS data used in this study is available in Supplementary Table 1.

The national VAS coverage mean among included countries was 58% and ranged from 28% to 83%, where countries with the lowest national VAS coverage were Papua New Guinea (28%), Haiti (28%) and Kyrgyzstan (39%). The percentage of children who did not recently consume vitamin A-rich foods among included countries ranged from 19% to 74%, where countries with the lowest percentage of consumption were Burkina Faso (74%), Niger (73%), and Ethiopia (70%). For vaccinations, the percentage of non-vaccinated children among included countries ranged from 1% to 48% for DTP1 and 13% to 69% for MCV1. A descriptive summary of the survey population of children aged 6-59 months for each country is provided in Supplementary Table 2.

VAS coverage by immediate & underlying determinants

In figures 1 and 2, we present VAS coverage for all countries stratified by recent consumption of vitamin A-rich foods and access to healthcare systems and services. VAS coverage was significantly higher among children who recently consumed vitamin A-rich foods, compared to children who did not in 85% (n=41) of countries (Figure 1). For access to healthcare systems and

For peer review only

services, VAS coverage was significantly higher in children who had access to their first dose of the DTP vaccine in 47 of the 49 countries compared to those who did not have access to these vaccines (Figure 2a). VAS coverage was significantly higher in children who had access to their first dose of MCV in all 49 countries (Figure 2b).

VAS coverage by geography and other enabling determinants

In Figure 3, VAS coverage is stratified by geographic covariates of vitamin A nutritional status for all countries. For place of residence, 31% of countries (n=15) had significantly lower VAS coverage in populations residing in rural versus urban areas. In countries where VAS coverage in rural residences exceeded that of urban residences, differences in coverage were never >8%. For administrative region, regions with the lowest and highest VAS coverage in each country had significant differences in 88% of countries (n=43) with spatial structure in sub-national VAS coverage visible when mapped (Figure 4 for Chad, India, Nigeria, Ethiopia, Yemen; full set of country maps available in Supplementary Figure 1-5).

In 73% of countries (n=36), VAS coverage was significantly higher in the wealthiest quintile of the population compared to the poorest quintile of the population, where the differences in coverage between poorest and wealthiest were greatest in Nigeria (33% difference), Cote D'Ivoire (25% difference), and the Democratic Republic of the Congo (25% difference) (Supplementary Figure 6). In 37% of countries (n=18), children of caregivers who were the most socially independent had significantly higher VAS coverage compared to caregivers who were the least socially independent (Supplementary Figure 7). In 20% of countries (n=10), VAS

For peer review only

coverage in children of caregivers who were most autonomous in their decision-making was significantly higher compared to children of the least autonomous caregivers (Supplementary Figure 8). Caregivers with higher educational attainment had higher VAS coverage for their children compared to caregivers with lower educational attainment in several countries (Supplementary Figure 9).

For peer review only

DISCUSSION

This study used open-source data to identify inequities in VAS program coverage so that strategies can be devised to improve VAS coverage among unreached populations. We found that children who likely have lower access to vitamin A-rich foods and who have impaired access to healthcare systems and services are also less likely to receive VAS. This pattern was consistent across most countries and highlights the challenges current VAS delivery faces to reach children that are likely most in need of VAS. The analysis also shows that in some countries, children missed by VAS also reside in the poorest households, in rural areas, and have caregivers who are more constrained by gender norms. Although these are the general trends, we also observed considerable variation between countries. While the use of DHS data to gain insight into VAS programs has been conducted in some countries [24–26], future analyses using DHS data can help inform VAS program operations in other country contexts. National programs can use the analysis presented here as a framework to improve targeting and prioritization of children who are likely to be most in need of VAS delivery programs.

The demonstrated use of DHS data to provide sub-national equity perspectives can provide useful VAS program insights. For example, this study indicated that lower VAS coverage for children who may have lower access to vitamin A-rich foods and healthcare systems and services was consistent across most countries. According to global estimates using UNICEF infant and young child feeding data, the consumption of vitamin A-rich foods by complementary feeding children is lowest in poorer and more rural children [27] suggesting that in combination multiple enabling determinants affecting nutrition are likely to contribute

For peer review only

to reduced access to vitamin A-rich foods. However, this study suggests that in many countries, VAS coverage is either equal or lower in these rural and poorest populations despite likely having greater need for VAS.

To address these gaps, VAS programs may benefit from being more aligned with routine community-based delivery programs. In children aged 6 – 11 months, in countries where VAS coverage between vaccinated and unvaccinated children diverges, there is a potential opportunity to improve VAS coverage by aligning programs more closely to the country's EPI systems. This alignment with EPI systems will require program reforms, including the creation of a six month contact point in routine systems, inclusion of VAS dosing schedules on child health cards [28], and strengthening supply chains for vaccines and vitamin A capsules to ensure concomitant availability. For older children who are not serviced by the EPI (12 – 59 months), there may be other established routine early childhood programs where the routine delivery of VAS could be integrated, such as growth monitoring and promotion, counselling on breastfeeding and complementary feeding, early childhood development programs and the early detection and treatment of severe wasting [29]. Current reliance on polio vaccination campaigns to deliver VAS has presented a risk of declined VAS coverage as polio programs cease [30], so identifying other routine community-based delivery programs to integrated VAS should be prioritized.

As the global vitamin A landscape evolves, it is important for national governments to consider how VAS programs can be positioned in combination with other parallel vitamin A interventions

For peer review only

to reduce risks for deficiency for all children. VAS has short-term benefits for children (boosting serum retinol for approximately two months after administration) [31], so other interventions are necessary to sustainably maintain adequate vitamin A intake through the diet. For the countries included in this study, 51% (n=25) have nationally mandated the large-scale vitamin A fortification of industrially produced foods items [32]. However, poorer and rural populations - where VAS coverage is often lower – often consume small quantities of fortified food items (e.g., cooking oil, sugar, wheat flour) and may therefore derive limited benefit from industrial vitamin A fortification schemes [33]. Programs aimed at broader improvements in dietary diversity have also been recommended [34], but variation in diets between and within countries pose challenges when scaling up across different contexts. With multiple parallel vitamin A interventions, future studies that simultaneously consider the individual and combined contributions of all vitamin A interventions could help governments orient their national vitamin A strategies.

Evaluating equity dimensions of VAS programs by stratifying coverage estimates using multiple variables can help identify potential gaps in national programs' delivery strategies. This analysis drew from the DHS Household and Woman's Questionnaires to identify enabling determinants to undernutrition that could affect VAS coverage. If differences in VAS coverage are detected between populations, some of these geographic determinants (e.g., place of residence, administrative regions of the country) can serve as indicators that lead to practical recommendations for programs to adopt to bridge coverage gaps (e.g., strengthening programs in a specific region of the country or for a specific demographic). In contrast, other

For peer review only

determinants, while useful to evaluate whether VAS programs are broadly equitable, lack clear operational directions on how programs can specifically target populations that are left behind (e.g., socioeconomic position, caregiver's social independence, caregiver's decision-making autonomy). This study explored several different indicators made available in the DHS, and VAS programs could benefit from further research aimed at interpreting the combination of multiple indicators in the context of a country's current VAS delivery strategy (e.g., campaign versus routine delivery).

DHS data can be used to support a variety of nationally implemented child health programs, including developing strategies to increase overall VAS coverage. Insights into national VAS programs that can be drawn using analyses of DHS data include VAS coverage differences over multiple survey years, differences between sub-populations, the potential contributions of risk factors affecting VAS coverage, and differences between sub-national geographies and other spatial analyses [15]. DHS data is becoming increasingly more available in several low and lower-middle income countries and is being used more frequently in peer-reviewed studies to support evidence-based approaches to a variety of public health policies and programs [14]. While the importance of the data generated by the DHS program is reflected through increasing and continued investment, additional investment is necessary to prioritize training and capacity of local statisticians and researchers to use data from the DHS program to inform VAS programs at national levels.

For peer review only

When using DHS data to evaluate VAS programs, there are several limitations that are important to consider. First, the question specific to VAS in the DHS questionnaire is dependent on recall by the caregiver of the child. The percentage of respondents who could prove VAS reception using home-based vaccination records varied between countries, but for most countries (69%; n=34) less than half of respondents had proof of VAS reception (Supplementary Figure 10). For children whose records depend solely on recall by the caregiver, recall error (e.g., confusing VAS droplets with polio droplets, imprecision in the exact date of reception, confusion between multiple children) is more likely. Second, the dietary data collected as part of the DHS only contains one dichotomous recall of whether vitamin A-rich foods were consumed in the past 24 hours by complementary feeding children. No information is available regarding food consumption quantities, weekly variation in diets, or for children aged 24 – 59 months. With this kind of dietary data, it is not possible to understand whether VAS is being administered to children who have inadequate dietary vitamin A intake or who have intakes that exceed daily upper limits to put children at risk for toxicity. To fully understand whether VAS programs are addressing children with the greatest vitamin A needs, VAS coverage should be estimated alongside other sub-national nutritional assessment data (e.g., vitamin A inadequacy from dietary assessment) to identify populations with both inadequate dietary vitamin A intake and low VAS coverage. Third, DHS data are not available in all countries, and in countries where they are available, they are only collected approximately every five years. This study included most countries where recent DHS data are available, but in countries and survey years where DHS are not conducted, these kinds of analyses are not possible. Considering these limitations, VAS coverage data available as part of the DHS is not recommended to replace two-

For peer review only

dose coverage estimates. However, two-dose coverage estimates and DHS data analyzed in tandem can provide a more complete perspective to inform national programs on overall program performance and how to potentially reach children who are being missed by current delivery strategies.

For peer review only

CONCLUSION

This study presents a method that can be useful to inform national governments on how to orient their VAS delivery strategy with the potential to integrate with other available data to identify populations with the greatest unmet vitamin A needs. Three decades after the WHO first recommended high-dose vitamin A supplements to infants and children aged 6 – 59 months, the international community has galvanized support to provide countries burdened with high risks for vitamin A deficiency with necessary material resources to progress towards universal VAS coverage. Despite strong political support, in many country settings, VAS programs are unable to reach all eligible infants and children, resulting in a need for evaluations to describe populations that are left behind by current delivery strategies. Evaluations of these sorts, however, require representative subnational data, such as DHS, to describe multiple dimensions of equity to adequately identify populations with the greatest needs and those that are consistently missed. By evaluating the equity dimensions of micronutrient programs, such as VAS, this study can help frame the micronutrient policy agenda towards addressing underlying determinants of undernutrition to inform priority settings and target resources according to need.

For peer review only

Contributions: K.T., K.P.A. and A.H. designed the study. K.T., A.I., K.P.A., and A.H. defined the variables for disaggregation. K.T. and H.E. wrote the code for data extraction and analysis. All authors critically reviewed and approved the final manuscript.

Funding: No funding was provided for this study. K.T., K.P.A., F.S., and E.J.M.J. receive funding from the Bill and Melinda Gates Foundation through the Micronutrient Action Policy Support (MAPS) project (INV-002855). K.T., A.I., G.M., and A.H. are staff members at UNICEF. The contents of this article do not necessarily reflect the views of the Bill and Melinda Gates Foundation or UNICEF.

Competing Interests: None declared

Data availability statement: Data are available in a public, open access repository. The data used are DHS and are publicly available at https://dhsprogram.com/data/available-datasets.cfm.

Ethics statement: This work is a secondary analysis of publicly available, de-identified data collected as part of the DHS. No Ethics Committee or Institutional Board approval is required.

For peer review only

REFERENCES

1 United Nations Children's Fund. UNICEF Nutrition Strategy 2020–2030. New York, USA: UNICEF, 2020.

2 Independent Expert Group of the Global Nutrition Report. 2020 Global Nutrition Report: Act on equity to end malnutrition. Bristol, United Kingdom: Development Initiatives Poverty Research Ltd., 2020. doi:10.2499/9780896295841

3 Stevens GA, Bennett JE, Hennocq Q, *et al.* Trends and mortality effects of vitamin A deficiency in children in 138 low-income and middle-income countries between 1991 and 2013: A pooled analysis of population-based surveys. *Lancet Glob Heal* Published Online First: 2015. doi:10.1016/S2214-109X(15)00039-X

4 Imdad A, Mayo-Wilson E, Herzer K, *et al.* Vitamin A supplementation for preventing morbidity and mortality in children from six months to five years of age. *Cochrane Database Syst Rev* Published Online First: 2017. doi:10.1002/14651858.CD008524.pub3

5 Horton S, Blum LS, Diouf M, *et al.* Delivering vitamin A supplements to children aged 6-59 months: Comparing delivery through campaigns and through routine health services in Senegal. *Curr Dev Nutr* 2018;**2**:nzy006. doi:10.1093/cdn/nzy006

6 Imdad A, Herzer K, Mayo-Wilson E, *et al.* Vitamin A supplementation for preventing morbidity and mortality in children from 6 months to 5 years of age. In: *Cochrane Database of Systematic Reviews*. 2010. doi:10.1002/14651858.cd008524.pub2

7 World Health Organization. Guideline : Vitamin A supplementation in infants and children 6 – 59 months of age. Geneva, Switzerland, WHO, 2011.

8 United Nations Children's Fund. Coverage at a Crossroads: New directions for vitamin A supplementation programmes. New York, USA: UNICEF, 2018.

9 Fawzi WW, Wang D. When should universal distribution of periodic high-dose vitamin A to children cease? *Am J Clin Nutr* 2021;**113**:769–71. doi:10.1093/ajcn/nqaa428

10 World Health Organization. Essential Program on Vaccination: Vitamin A. 2021.https://www.who.int/teams/immunization-vaccines-and-biologicals/essential-programme-on-immunization/integration/linking-with-other-health-interventions/vitamin-a (accessed 7 Oct 2021).

11 Sbarra AN, Rolfe S, Nguyen JQ, *et al.* Mapping routine measles vaccination in low- and middle-income countries. *Nature* Published Online First: 2021. doi:10.1038/s41586-020-03043-4

12 Hosseinpoor AR, Bergen N, Schlotheuber A, *et al.* State of inequality in diphtheria-tetanus-pertussis immunisation coverage in low-income and middle-income countries: a multicountry study of household health surveys. *Lancet Glob Heal* Published Online First: 2016. doi:10.1016/S2214-109X(16)30141-3

13 United Nations Children's Fund. Estimates of Vitamin A Supplementation Coverage in Preschool-age Children: Methods and processes for the UNICEF global database. New York, USA: UNICEF, 2020.

14 Short Fabic M, Choi Y, Bird S. A systematic review of Demographic and Health Surveys: data availability and utilization for research. *Bull World Health Organ* Published Online First: 2012. doi:10.2471/blt.11.095513

15 Corsi DJ, Neuman M, Finlay JE, *et al.* Demographic and health surveys: A profile. *Int J Epidemiol* Published Online First: 2012. doi:10.1093/ije/dys184

For peer review only

16 ICF International. Demographic and Health Surveys Standard Recode Manual for DHS7. Rockville, USA: ICF International, Inc., 2018.

17 ICF International. Demographic and Health Survey Interviewer's Manual. Rockville, USA: ICF International, Inc., 2020.

18 World Health Organization. Complementary feeding of young children in developing countries: a review of current scientific knowledge. Geneva, Switzerland: WHO, 1998. https://apps.who.int/iris/handle/10665/65932

19 Ewerling F, Lynch JW, Victora CG, *et al.* The SWPER index for women's empowerment in Africa: development and validation of an index based on survey data. *Lancet Glob Heal* Published Online First: 2017. doi:10.1016/S2214-109X(17)30292-9

20 Pullum T, Ren R, Elkasabi M. Webinar: Weights & Adjustments for DHS Surveys. DHS Progr. 2015. https://www.youtube.com/watch?v=5UDPYLwWHIA (accessed 7 Oct 2021).

21 Watson OJ, FitzJohn R, Eaton JW. rdhs: an R package to interact with The Demographic and Health Surveys (DHS) Program datasets. *Wellcome Open Res* Published Online First: 2019. doi:10.12688/wellcomeopenres.15311.1

22 Freedman Ellis G, Lumley T, Zoltak T, *et al.* srvyr: 'dplyr'-Like Syntax for Summary Statistics of Survey Data. 2021.https://cran.r-project.org/web/packages/srvyr/index.html

23 Wickham H, Averick M, Bryan J, *et al.* Welcome to the Tidyverse. *J Open Source Softw* 2019;**4**:1686. doi:10.21105/joss.01686

24 Aghaji AE, Duke R, Aghaji UCW. Inequitable coverage of vitamin A supplementation in Nigeria and implications for childhood blindness. *BMC Public Health* 2019;**19**:282. doi:10.1186/s12889-019-6413-1

25 Millogo O, Doamba JEO, Sié A, *et al.* Geographical variation in the association of child, maternal and household health interventions with under-five mortality in Burkina Faso. *PLoS One* 2019;**14**:e0218163. doi:10.1371/journal.pone.0218163

26 Ribaudo I, Aramburú-Duclos CB, Blitchtein D. Is Vitamin A Supplementation Associated With Anemia in Children Under 5 Years of Age in Peru: Secondary Analysis of the "Demographic Health Survey" 2015-2018? *Glob Pediatr Heal* 2021;**8**:1–11. doi:10.1177/2333794X211048385

27 White JM, Bégin F, Kumapley R, *et al.* Complementary feeding practices: Current global and regional estimates. *Matern Child Nutr* Published Online First: 2017. doi:10.1111/mcn.12505

28 Hodges MH, Sesay FF, Kamara HI, *et al.* Integrating Vitamin A Supplementation at 6 months into the Expanded Program of Immunization in Sierra Leone. *Matern Child Health J* Published Online First: 2015. doi:10.1007/s10995-015-1706-1

29 Hasman A, Moloney G, Aguayo V. Regular vitamin A supplementation: Prioritizing the youngest children. *Am J Clin Nutr* 2021;**114.1**:390–1. doi:10.1093/ajcn/nqab179

30 Chehab ET, Anya BPM, Onyango AW, *et al.* Experience of integrating vitamin A supplementation into polio campaigns in the African Region. *Vaccine* Published Online First: 2016. doi:10.1016/j.vaccine.2016.05.056

31 Palmer AC, West KP, Dalmiya N, *et al.* The use and interpretation of serum retinol distributions in evaluating the public health impact of vitamin A programmes. Public Health Nutr. 2012. doi:10.1017/S1368980012000560

32 Food Fortification Initiative, Global Alliance for Improved Nutrition, Network IG, *et al.*

For peer review only

Global Fortification Data Exchange. 2018. https://fortificationdata.org/ (accessed 7 Oct 2021).

33 Tang K, Adams KP, Ferguson EL, *et al.* Modeling food fortification contributions to micronutrient requirements in Malawi using Household Consumption and Expenditure Surveys. *Ann N Y Acad Sci* 2021;:1–18. doi:10.1111/nyas.14697

34 Karlsson O, Kim R, Hasman A, *et al.* Consumption of Vitamin-A-Rich Foods and Vitamin A Supplementation for Children under Two Years Old in 51 Low- and Middle-Income Countries. *Nutrients* 2022;**14**:188. doi:10.3390/nu14010188

For peer review only

FIGURE LEGEND

Figure 1. Vitamin A supplementation (VAS) coverage among children 6-23 months who have and have not recently consumed vitamin A-rich foods (in the 24 hours prior to the interview). Philippines DHS did not collect data on child consumption of vitamin A-rich foods.

Figure 2. Vitamin A supplementation (VAS) coverage among children who have and have not received (A) the first dose of the diphtheria-tetanus-pertussis (DTP) vaccine and (B) the first dose of the measles containing vaccine (indicator of access to healthcare systems and services).

Figure 3. Vitamin A supplementation (VAS) coverage disaggregated by geography: (A) rural vs urban residence and (B) administrative regions.

Figure 4. Vitamin A supplementation (VAS) coverage maps by administrative regions (maps of all countries available in the supplementary material)

Supplementary Figure 1. Vitamin A supplementation (VAS) coverage between administrative regions for included countries in East and Southern Africa.

Supplementary Figure 2. Vitamin A supplementation (VAS) coverage between administrative regions for included countries in West and Central Africa.

Supplementary Figure 3. Vitamin A supplementation (VAS) coverage between administrative regions for included countries in South Asia, East Asia, and the Western Pacific.

Supplementary Figure 4. Vitamin A supplementation (VAS) coverage between administrative regions for included countries in the Eastern Mediterranean Region.

Supplementary Figure 5. Vitamin A supplementation (VAS) coverage between administrative regions for included countries in the Americas.

Supplementary Figure 6. Vitamin A supplementation (VAS) coverage among children from households of the poorest versus the richest socioeconomic quintiles.

Supplementary Figure 7. Differences in vitamin A supplementation (VAS) coverage between children whose caregivers are the least versus the most socially independent in their country.

Supplementary Figure 8. Differences in vitamin A supplementation (VAS) coverage between children whose caregivers have the least versus the most decision-making autonomy in their country.

Supplementary Figure 9. Differences in vitamin A supplementation (VAS) coverage between children based on the educational attainment of the child's caregiver.

Supplementary Figure 10. Proportion of children who had received vitamin A supplementation (VAS) whose caregiver confirmed VAS reception with a vaccination card.

For peer review only

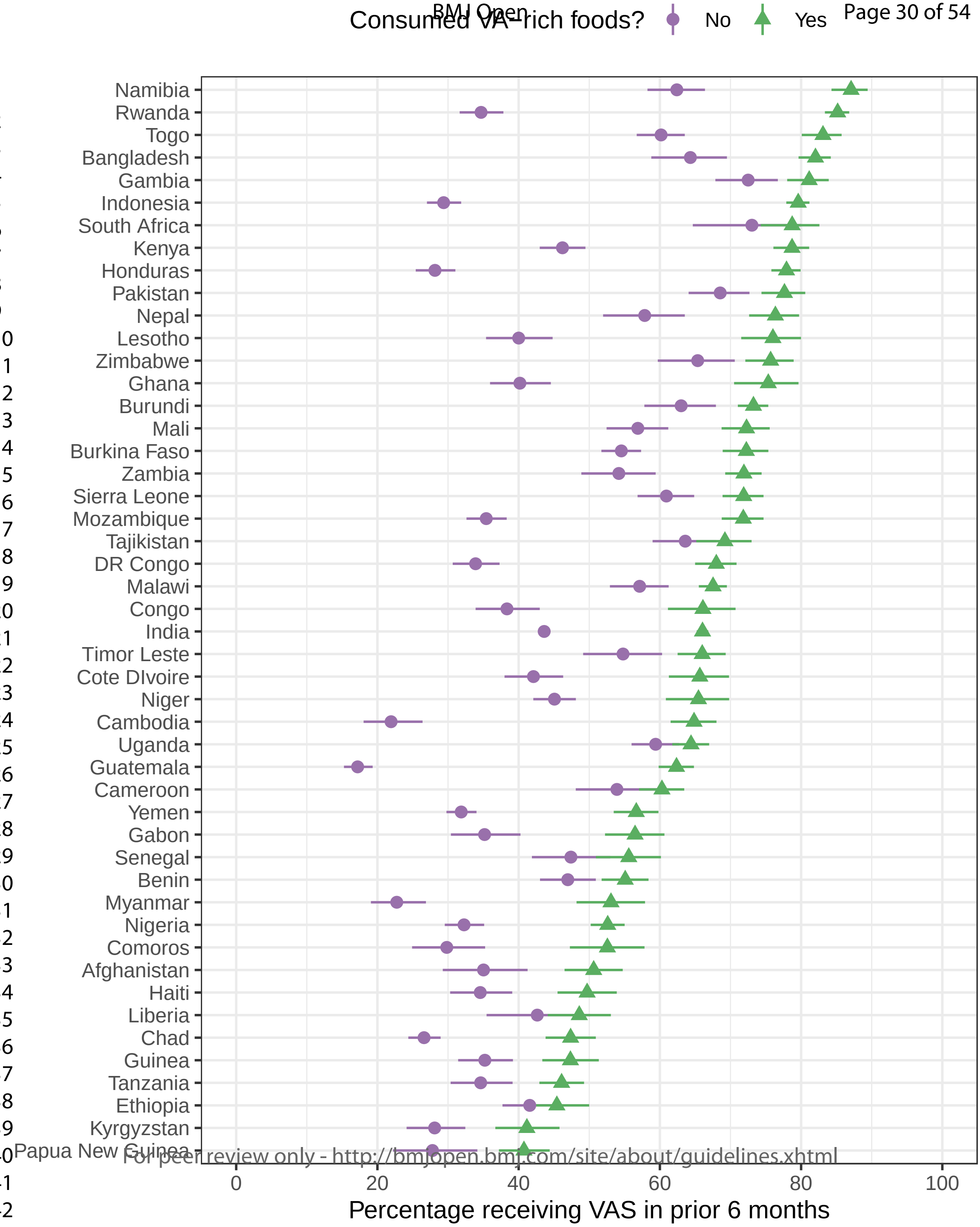
Consumed VA−rich foods?
No
Yes
Namibia
Rwanda
Togo
Bangladesh
Gambia
Indonesia
South Africa
Kenya
Honduras
Pakistan
Nepal
Lesotho
Zimbabwe
Ghana
Burundi
Mali
Burkina Faso
Zambia
Sierra Leone
Mozambique
Tajikistan
DR Congo
Malawi
Congo
India
Timor Leste
Cote DIvoire
Niger
Cambodia
Uganda
Guatemala
Cameroon
Yemen
Gabon
Senegal
Benin
Myanmar
Nigeria
Comoros
Afghanistan
Haiti
Liberia
Chad
Guinea
Tanzania
Ethiopia
Kyrgyzstan
Papua New Guinea
0
20
40
60
80
100
Percentage receiving VAS in prior 6 months

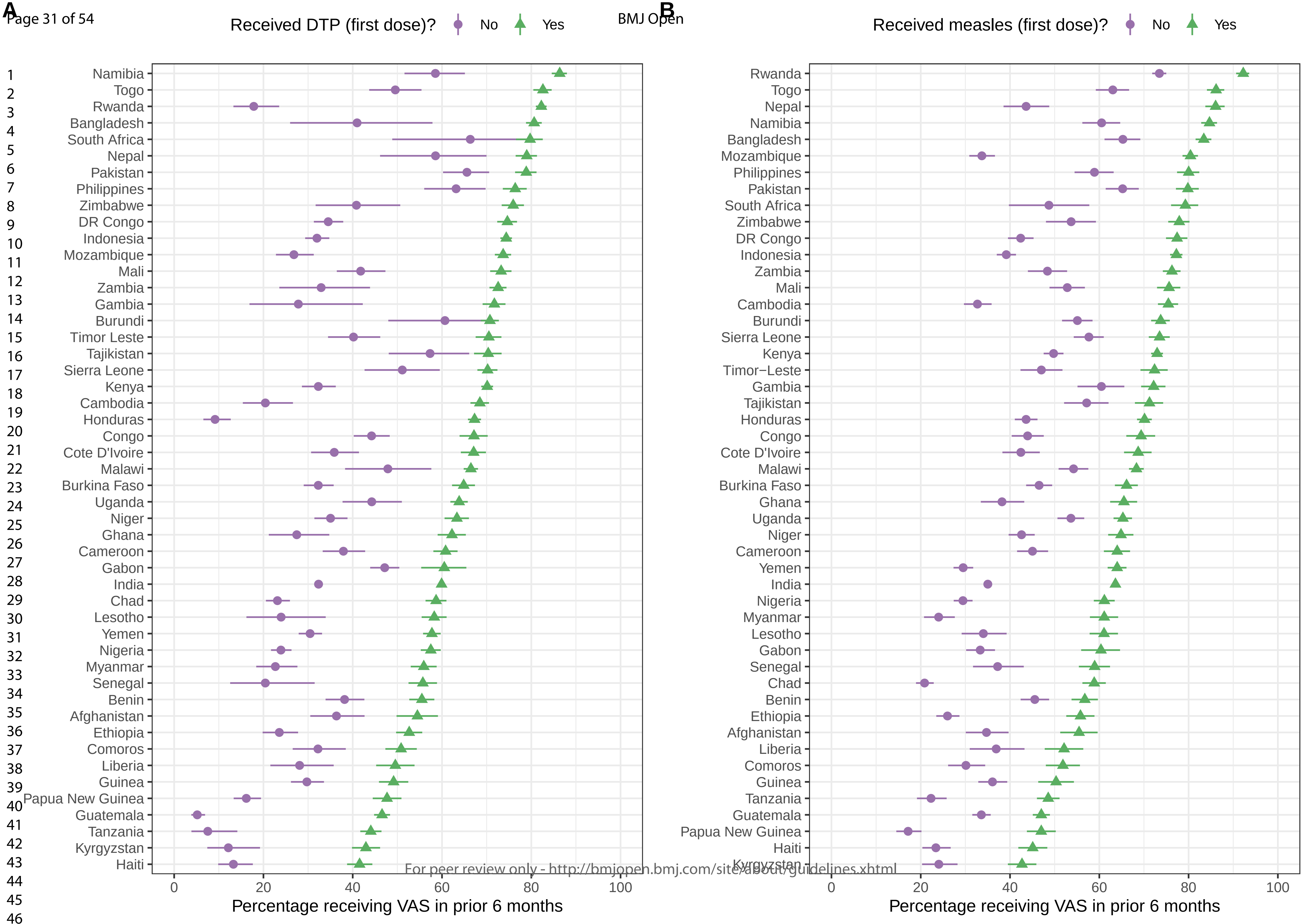
A
Received DTP (first dose)?
No
Yes
Namibia
Togo
Rwanda
Bangladesh
South Africa
Nepal
Pakistan
Philippines
Zimbabwe
DR Congo
Indonesia
Mozambique
Mali
Zambia
Gambia
Burundi
Timor Leste
Tajikistan
Sierra Leone
Kenya
Cambodia
Honduras
Congo
Cote D'Ivoire
Malawi
Burkina Faso
Uganda
Niger
Ghana
Cameroon
Gabon
India
Chad
Lesotho
Yemen
Nigeria
Myanmar
Senegal
Benin
Afghanistan
Ethiopia
Comoros
Liberia
Guinea
Papua New Guinea
Guatemala
Tanzania
Kyrgyzstan
Haiti
0
20
40
60
80
100
Percentage receiving VAS in prior 6 months
B
Received measles (first dose)?
No
Yes
Rwanda
Togo
Nepal
Namibia
Bangladesh
Mozambique
Philippines
Pakistan
South Africa
Zimbabwe
DR Congo
Indonesia
Zambia
Mali
Cambodia
Burundi
Sierra Leone
Kenya
Timor−Leste
Gambia
Tajikistan
Honduras
Congo
Cote D'Ivoire
Malawi
Burkina Faso
Ghana
Uganda
Niger
Cameroon
Yemen
India
Nigeria
Myanmar
Lesotho
Gabon
Senegal
Chad
Benin
Ethiopia
Afghanistan
Liberia
Comoros
Guinea
Tanzania
Guatemala
Papua New Guinea
Haiti
Kyrgyzstan
0
20
40
60
80
100
Percentage receiving VAS in prior 6 months

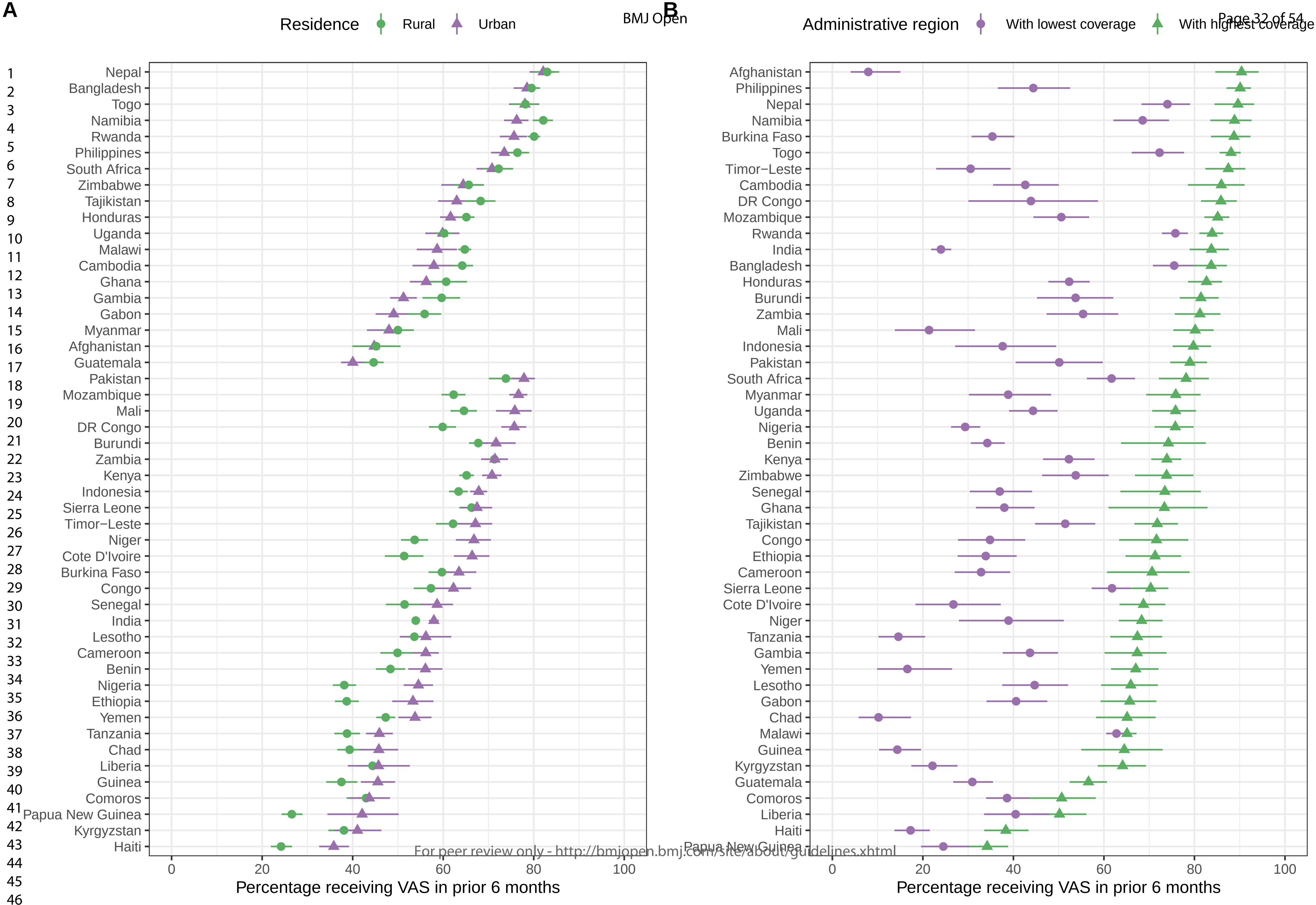
A
Residence
Rural
Urban
Nepal
Bangladesh
Togo
Namibia
Rwanda
Philippines
South Africa
Zimbabwe
Tajikistan
Honduras
Uganda
Malawi
Cambodia
Ghana
Gambia
Gabon
Myanmar
Afghanistan
Guatemala
Pakistan
Mozambique
Mali
DR Congo
Burundi
Zambia
Kenya
Indonesia
Sierra Leone
Timor−Leste
Niger
Cote D'Ivoire
Burkina Faso
Congo
Senegal
India
Lesotho
Cameroon
Benin
Nigeria
Ethiopia
Yemen
Tanzania
Chad
Liberia
Guinea
Comoros
Papua New Guinea
Kyrgyzstan
Haiti
0 20 40 60 80 100
Percentage receiving VAS in prior 6 months
B
Administrative region
With lowest coverage
With highest coverage
Afghanistan
Philippines
Nepal
Namibia
Burkina Faso
Togo
Timor−Leste
Cambodia
DR Congo
Mozambique
Rwanda
India
Bangladesh
Honduras
Burundi
Zambia
Mali
Indonesia
Pakistan
South Africa
Myanmar
Uganda
Nigeria
Benin
Kenya
Zimbabwe
Senegal
Ghana
Tajikistan
Congo
Ethiopia
Cameroon
Sierra Leone
Cote D'Ivoire
Niger
Tanzania
Gambia
Yemen
Lesotho
Gabon
Chad
Malawi
Guinea
Kyrgyzstan
Guatemala
Comoros
Liberia
Haiti
Papua New Guinea
0 20 40 60 80 100
Percentage receiving VAS in prior 6 months

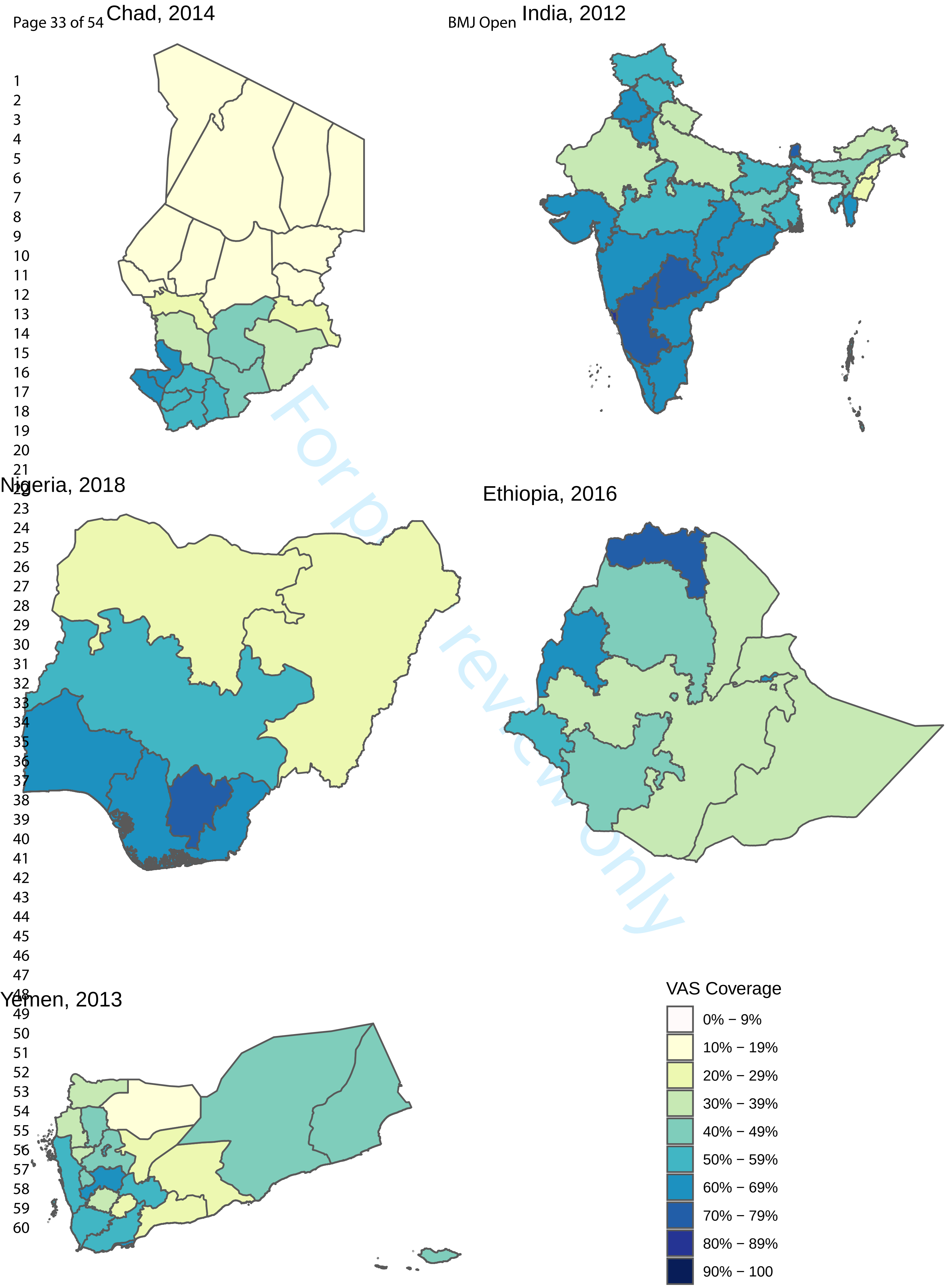

For peer review only

Evaluating equity dimensions of infant and child vitamin A supplementation programs using Demographic and Health Surveys from 49 countries

- SUPPLEMENTARY MATERIAL –

Table of Contents

Supplementary Table 1. Countries included in the study and summary information on their respective Demographic and Health Surveys 2

Supplementary Table 2. Descriptive summary of the survey population of children aged 6-59 months for each country included in this study 4

Supplementary Figure 1. Vitamin A supplementation (VAS) coverage between administrative regions for included countries in East and Southern Africa. 6

Supplementary Figure 2. Vitamin A supplementation (VAS) coverage between administrative regions for included countries in West and Central Africa. 9

Supplementary Figure 3. Vitamin A supplementation (VAS) coverage between administrative regions for included countries in South Asia, East Asia, and the Western Pacific. 13

Supplementary Figure 4. Vitamin A supplementation (VAS) coverage between administrative regions for included countries in the Eastern Mediterranean Region. 16

Supplementary Figure 5. Vitamin A supplementation (VAS) coverage between administrative regions for included countries in the Americas. 17

Supplementary Figure 6. Vitamin A supplementation (VAS) coverage among children from households of the poorest versus the richest socioeconomic quintiles. 18

Supplementary Figure 7. Differences in vitamin A supplementation (VAS) coverage between children whose caregivers are the least versus the most socially independent in their country. 19

Supplementary Figure 8. Differences in vitamin A supplementation (VAS) coverage between children whose caregivers have the least versus the most decision-making autonomy in their country. 20

Supplementary Figure 9. Differences in vitamin A supplementation (VAS) coverage between children based on the educational attainment of the child's caregiver. 21

Supplementary Figure 10. Proportion of children who had received vitamin A supplementation (VAS) whose caregiver confirmed VAS reception with a vaccination card. 22

For peer review only - http://bmjopen.bmj.com/site/about/guidelines.xhtml

Supplementary Table 1. Countries included in the study and summary information on their respective Demographic and Health Surveys

Country	Survey year	Women surveyed (n)	Children recalled (n)
Afghanistan	2015	29,461	29,312
Bangladesh	2017	20,127	4137
Benin	2017	15,928	6378
Burkina Faso	2010	17,087	13,644
Burundi	2016	17,269	6161
Cambodia	2014	17,578	6909
Cameroon	2018	13,527	4337
Chad	2014	17,719	16,441
Comoros	2012	5329	2879
Congo	2011	10,819	8725
Cote D'Ivoire	2012	10,060	6882
Dem. Rep. of the Congo	2013	18,827	16,952
Ethiopia	2016	15,683	9504
Gabon	2012	8422	5747
Gambia	2019	11,865	3769
Ghana	2014	9396	5531
Guatemala	2015	25,914	12,071
Guinea	2018	10,874	3288
Haiti	2016	14,371	2922
Honduras	2011	22,757	10,592
India	2012	699,686	244,869
Indonesia	2017	45,607	17,367
Kenya	2014	31,079	19,887
Kyrgyzstan	2012	8208	4247
Lesotho	2014	6621	2855
Liberia	2019	8065	2493
Malawi	2015	24,562	7870
Mali	2018	10,519	4442
Mozambique	2011	13,745	10,214
Myanmar	2016	12,885	4550
Namibia	2013	9176	4818
Nepal	2016	12,862	2354
Niger	2012	11,160	11,338
Nigeria	2018	41,821	14,434
Pakistan	2017	15,068	5589
Papua New Guinea	2017	15,198	4436
Philippines	2017	25,074	4882
Rwanda	2015	13,497	7504
Senegal	2018	9414	3147
Sierra Leone	2019	15,574	4344
South Africa	2016	8514	3413
Tajikistan	2017	10,718	3016
Tanzania	2015	13,266	4864
Timor-Leste	2016	12,607	3332
Togo	2013	9480	6467
Uganda	2016	18,506	7138
Yemen	2013	25,434	15,136

For peer review only

Zambia	2018	13,683	4616
Zimbabwe	2015	9955	2775

For peer review only

For peer review only - http://bmjopen.bmj.com/site/about/guidelines.xhtml

Supplementary Table 2. Descriptive summary of the survey population of children aged 6-59 months for each country included in this study

Country	Received VAS in past 6 months, % (95%CI)	Did not recently consume vitamin A rich foods	Did not received DTP1	Did not received MCV1	Rural
Afghanistan	45.1 (40.9, 49.4)	62.5 (59.7, 65.2)	35.8 (31.9, 39.9)	49.3 (46.1, 52.6)	76.8 (71.4, 81.4)
Bangladesh	79.2 (77.6, 80.8)	41.8 (39.9, 43.7)	1.5 (1.0, 2.3)	19.7 (18.3, 21.3)	71.4 (68.4, 76.1)
Benin	51.4 (48.9, 53.9)	53.8 (51.9, 55.8)	16.2 (14.4, 18.3)	42.7 (40.5, 65.9)	61.3 (56.6, 65.9)
Burkina Faso	60.4 (57.8, 65.9)	73.6 (71.8, 75.4)	12.6 (11.3, 13.9)	28.7 (27.3, 30.1)	82.7 (79.3, 85.6)
Burundi	68.1 (66.2, 69.9)	33.1 (31.6, 34.7)	1.3 (1.0, 1.7)	17.3 (16.2, 18.4)	91.1 (88.2, 93.3)
Cambodia	63.3 (61.1, 65.4)	35.2 (32.7, 37.9)	9.7 (8.6, 10.9)	28.5 (26.9, 30.1)	85.3 (82.0, 88.1)
Cameroon	52.7 (50.3, 55.2)	41.4 (39.3, 43.4)	17.0 (14.6, 19.6)	39.5 (36.9, 42.2)	55.3 (49.1, 61.4)
Chad	40.6 (38.3, 43.0)	65.2 (62.9, 67.4)	47.5 (44.8, 50.3)	47.4 (45.3, 49.5)	80.4 (76.5, 83.9)
Comoros	43.2 (39.8, 46.6)	43.7 (39.8, 47.6)	24.4 (21.5, 27.6)	36.9 (33.9, 40.0)	72.7 (65.6, 78.8)
Congo	60.3 (57.4, 63.1)	40.5 (37.7, 43.3)	24.9 (22.8, 27.2)	35.5 (33.3, 37.8)	39.5 (33.4, 46.0)
Cote D'Ivoire	57.0 (53.8, 60.3)	54.1 (51.6, 56.7)	26.5 (23.6, 29.6)	43.7 (40.9, 46.5)	62.3 (55.6, 68.5)
Dem. Rep. of the Congo	64.8 (62.3, 67.1)	37.1 (35.0, 39.2)	22.5 (20.3, 24.9)	36.0 (34.2, 37.9)	69.1 (63.4, 74.3)
Ethiopia	40.1 (37.9, 42.8)	69.9 (67.0, 72.7)	27.8 (25.0, 30.9)	60.2 (57.2, 63.1)	88.8 (85.4, 91.5)
Gabon	50.1 (46.7, 53.6)	48.2 (45.1, 51.2)	47.4 (44.5, 50.3)	36.2 (33.6, 38.8)	15.6 (11.9, 20.1)
Gambia	54.1 (51.6, 56.5)	56.3 (53.5, 59.0)	2.4 (1.7, 3.2)	19.8 (17.9, 21.9)	34.1 (27.6, 41.2)
Ghana	58.7 (55.6, 61.7)	49.0 (45.7, 52.2)	7.2 (6.2, 8.5)	24.9 (23.3, 26.6)	54.9 (48.5, 61.1)
Guatemala	43.0 (41.3, 44.7)	19.2 (18.2, 20.3)	8.6 (7.9, 9.3)	30.0 (29.0, 31.0)	64.0 (59.9, 67.9)
Guinea	40.0 (37.3, 42.6)	66.7 (64.1, 69.1)	36.6 (33.2, 40.1)	60.1 (56.5, 63.7)	71.0 (65.9, 75.7)
Haiti	28.3 (26.3, 30.4)	55.9 (53.1, 58.6)	19.3 (16.8, 22.0)	42.7 (39.8, 45.6)	64.4 (58.6, 69.9)
Honduras	63.5 (62.0, 64.9)	32.2 (30.4, 34.0)	4.8 (4.4, 5.3)	24.5 (23.6, 25.5)	53.8 (50.2, 57.3)
India	55.1 (54.6, 55.6)	38.2 (37.6, 38.7)	15.0 (14.7, 15.3)	27.7 (25.3, 30.1)	68.1 (62.1, 73.6)
Indonesia	65.6 (64.2, 67.0)	34.3 (32.6, 36.0)	16.5 (15.3, 17.7)	30.2 (28.9, 31.5)	50.1 (46.7, 53.6)
Kenya	67.2 (65.9, 68.5)	41.3 (38.9, 43.8)	5.7 (5.2, 6.2)	24.8 (23.8, 25.7)	64.3 (60.9, 67.6)
Kyrgyzstan	39.0 (36.1, 41.9)	47.2 (43.9, 50.6)	4.0 (3.2, 5.1)	19.0 (17.6, 20.4)	70.1 (63.4, 76.1)
Lesotho	54.4 (51.6, 57.1)	52.6 (48.8, 56.4)	6.6 (5.5, 7.9)	24.1 (22.3, 26.0)	71.0 (64.2, 76.9)
Liberia	45.1 (41.1, 49.1)	54.7 (51.6, 57.8)	9.4 (7.5, 11.6)	35.9 (32.7, 39.2)	47.2 (39.4, 55.0)
Malawi	63.9 (62.5, 65.3)	38.9 (37.5, 40.4)	2.6 (2.1, 3.2)	18.4 (17.3, 19.5)	86.7 (83.3, 89.5)
Mali	66.9 (64.4, 69.3)	55.2 (52.9, 57.6)	17.4 (15.2, 19.9)	36.7 (34.0, 39.4)	80.0 (74.7, 84.3)

For peer review only - http://bmjopen.bmj.com/site/about/guidelines.xhtml

Mozambique	66.3 (64.2, 68.4)	35.7 (33.7, 37.6)	15.0 (13.2, 16.9)	30.1 (28.2, 32.1)	72.2 (67.8, 76.2)
Myanmar	49.6 (46.6, 52.5)	46.2 (42.9, 49.6)	18.0 (15.6, 20.7)	31.3 (28.8, 33.9)	77.4 (72.3, 81.9)
Namibia	79.2 (77.4, 81.0)	46.1 (43.2, 49.0)	8.7 (7.6, 9.9)	22.1 (20.6, 23.6)	51.0 (45.6, 56.4)
Nepal	82.5 (80.4, 84.4)	43.7 (40.3, 47.1)	3.3 (2.3, 4.8)	18.8 (16.8, 20.9)	46.1 (39.5, 52.8)
Niger	55.5 (52.8, 58.1)	72.7 (70.6, 74.8)	22.5 (20.5, 24.6)	41.1 (39.3, 42.9)	86.3 (83.2, 89.0)
Nigeria	44.7 (42.6, 46.8)	53.7 (52.1, 55.3)	36.1 (34.0, 38.3)	50.7 (48.8, 52.6)	61.4 (58.0, 64.8)
Pakistan	75.1 (72.5, 77.6)	59.0 (56.0, 62.0)	15.9 (12.9, 19.4)	33.6 (30.4, 36.9)	68.0 (62.1, 73.4)
Papua New Guinea	28.2 (25.9, 30.6)	29.7 (27.4, 32.2)	35.5 (32.3, 38.9)	44.1 (40.7, 47.5)	89.6 (86.5, 92.0)
Philippines	75.1 (73.1, 77.0)	-	14.3 (12.5, 16.4)	28.4 (26.0, 30.8)	55.7 (51.2, 60.1)
Rwanda	79.3 (78.1, 80.6)	40.5 (38.5, 42.5)	3.7 (3.2, 4.4)	68.5 (66.0, 70.9)	83.1 (79.2, 86.4)
Senegal	54.2 (51.2, 57.2)	52.6 (49.3, 56.0)	3.9 (2.8, 5.6)	24.3 (22.0, 26.7)	61.8 (52.4, 70.3)
Sierra Leone	66.7 (64.5, 68.8)	52.8 (50.7, 54.8)	5.5 (4.6, 6.5)	32.3 (30.3, 34.4)	65.0 (60.3, 69.5)
South Africa	71.3 (68.8, 73.7)	44.3 (40.7, 48.0)	7.7 (6.1, 9.8)	13.4 (11.3, 15.9)	36.3 (31.2, 41.7)
Tajikistan	67.2 (64.3, 69.9)	58.7 (56.0, 61.4)	7.4 (6.1, 9.0)	23.8 (21.5, 26.2)	79.0 (74.6, 82.9)
Tanzania	40.6 (38.4, 42.9)	40.3 (38.1, 42.5)	3.7 (2.7, 5.0)	24.0 (22.0, 26.3)	72.9 (68.1, 77.2)
Timor-Leste	63.6 (60.7, 66.4)	45.0 (42.4, 47.7)	22.9 (20.4, 25.6)	36.3 (33.8, 38.9)	71.6 (65.4, 77.1)
Togo	78.1 (75.8, 80.3)	45.6 (43.5, 47.8)	11.1 (9.7, 12.7)	34.7 (32.4, 37.0)	63.7 (57.7, 69.3)
Uganda	60.1 (58.4, 61.9)	48.0 (46.2, 49.8)	6.1 (5.4, 7.1)	28.3 (26.9, 29.9)	78.4 (74.5, 81.8)
Yemen	49.1 (47.2, 50.9)	54.8 (52.8, 56.7)	28.9 (26.9, 31.0)	43.3 (41.4, 45.2)	72.7 (68.3, 76.7)
Zambia	71.4 (69.5, 73.1)	39.6 (37.5, 41.7)	1.9 (1.4, 2.5)	18.5 (17.1, 20.0)	64.4 (58.8, 69.6)
Zimbabwe	65.2 (62.6, 67.8)	41.7 (39.3, 44.1)	11.8 (9.8, 14.2)	27.7 (25.3, 30.1)	68.1 (62.1, 73.6)

For peer review only

Supplementary Figure 1. Vitamin A supplementation (VAS) coverage between administrative regions for included countries in East and Southern Africa.

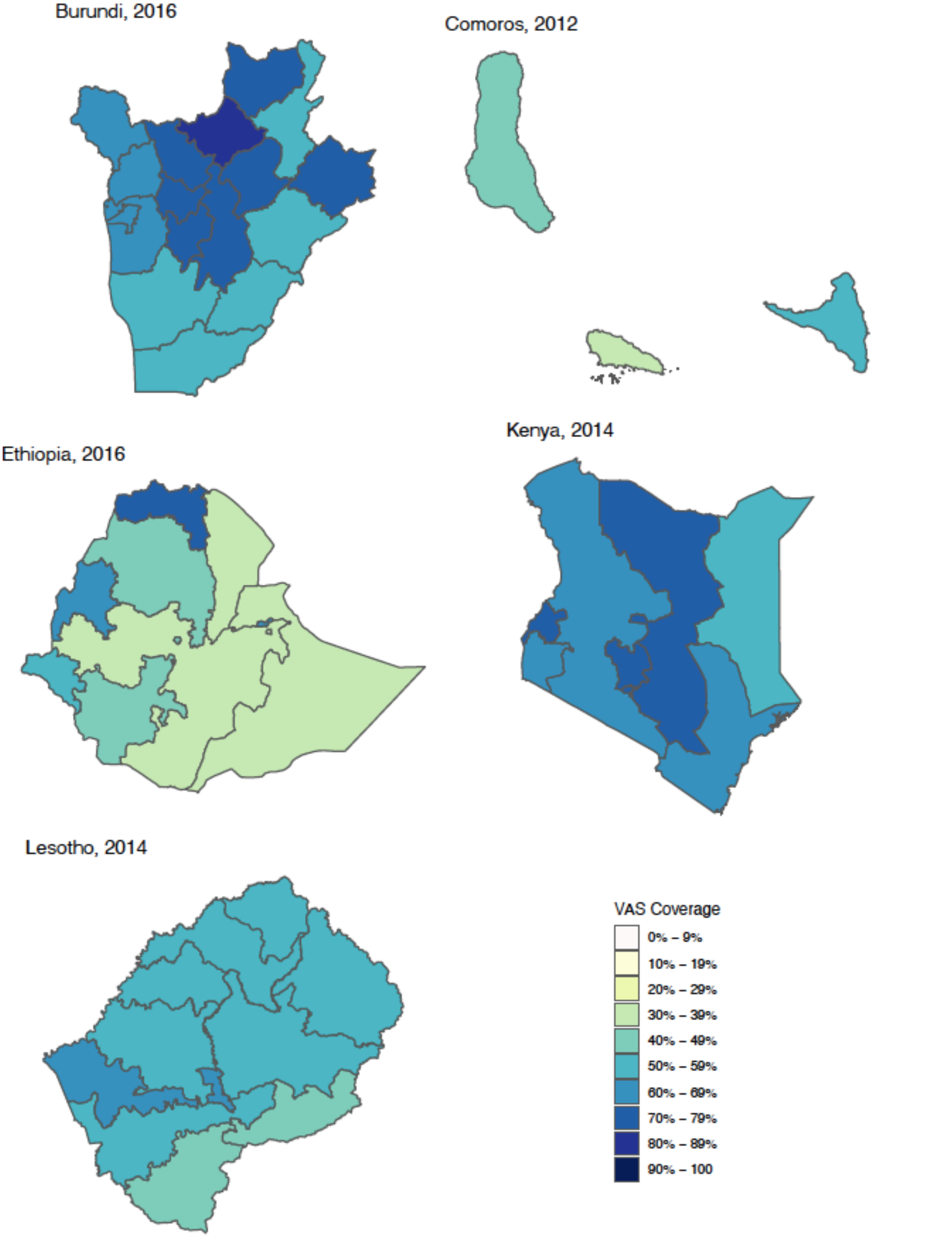

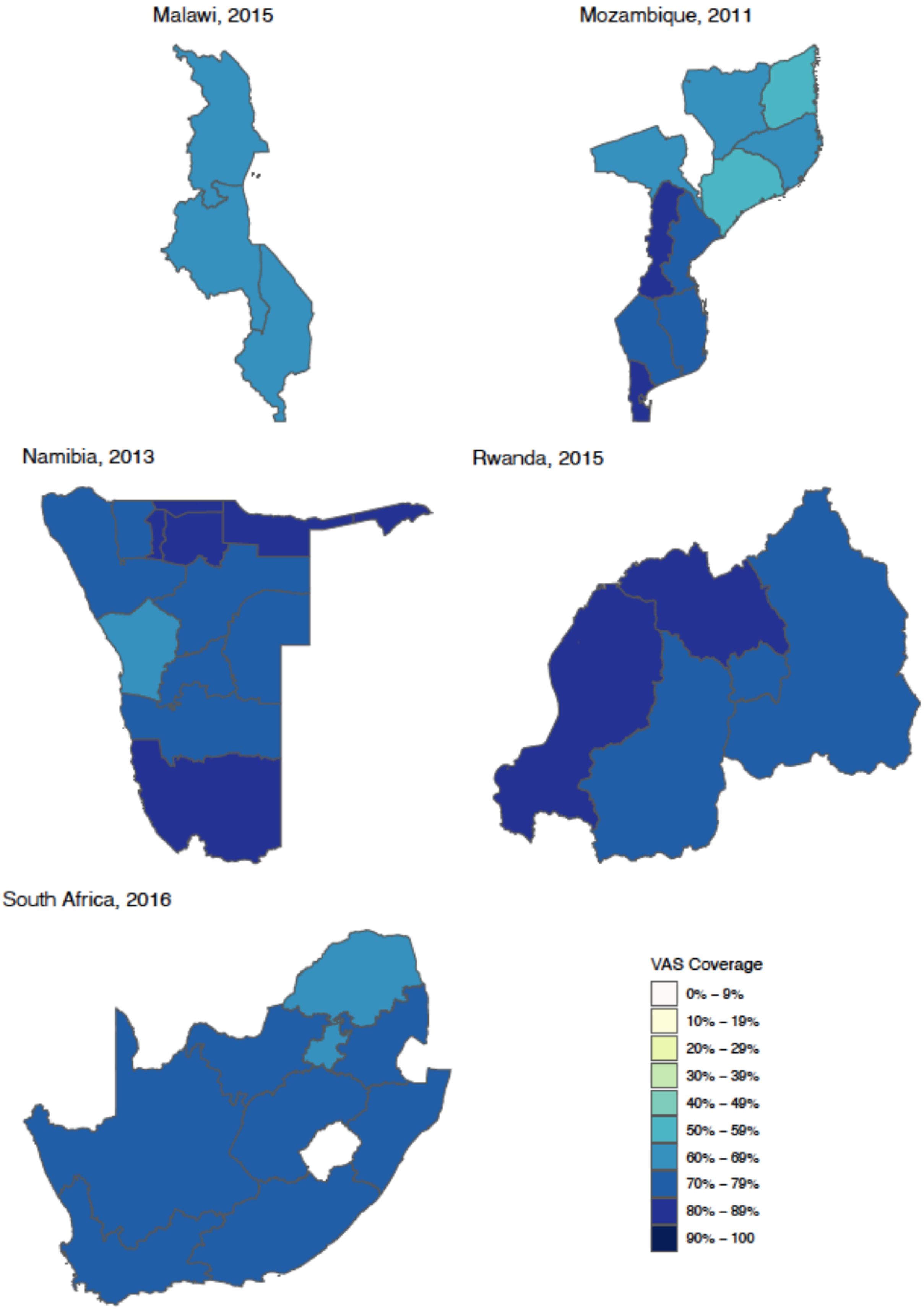
Malawi, 2015
Mozambique, 2011
Namibia, 2013
Rwanda, 2015
South Africa, 2016
VAS Coverage
0% – 9%
10% – 19%
20% – 29%
30% – 39%
40% – 49%
50% – 59%
60% – 69%
70% – 79%
80% – 89%
90% – 100

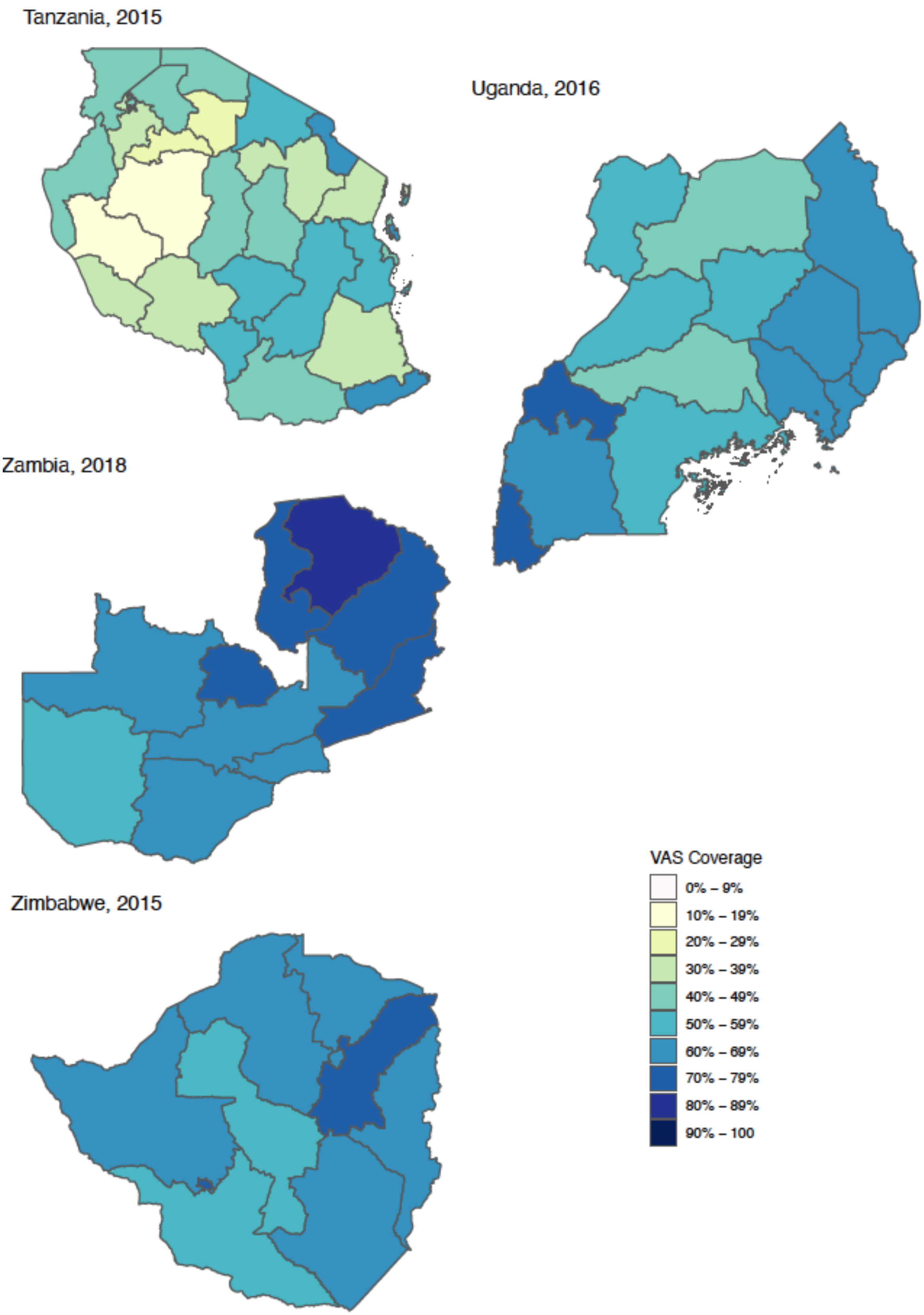
Tanzania, 2015
Uganda, 2016
Zambia, 2018
Zimbabwe, 2015
VAS Coverage
0% – 9%
10% – 19%
20% – 29%
30% – 39%
40% – 49%
50% – 59%
60% – 69%
70% – 79%
80% – 89%
90% – 100

8

Supplementary Figure 2. Vitamin A supplementation (VAS) coverage between administrative regions for included countries in West and Central Africa.

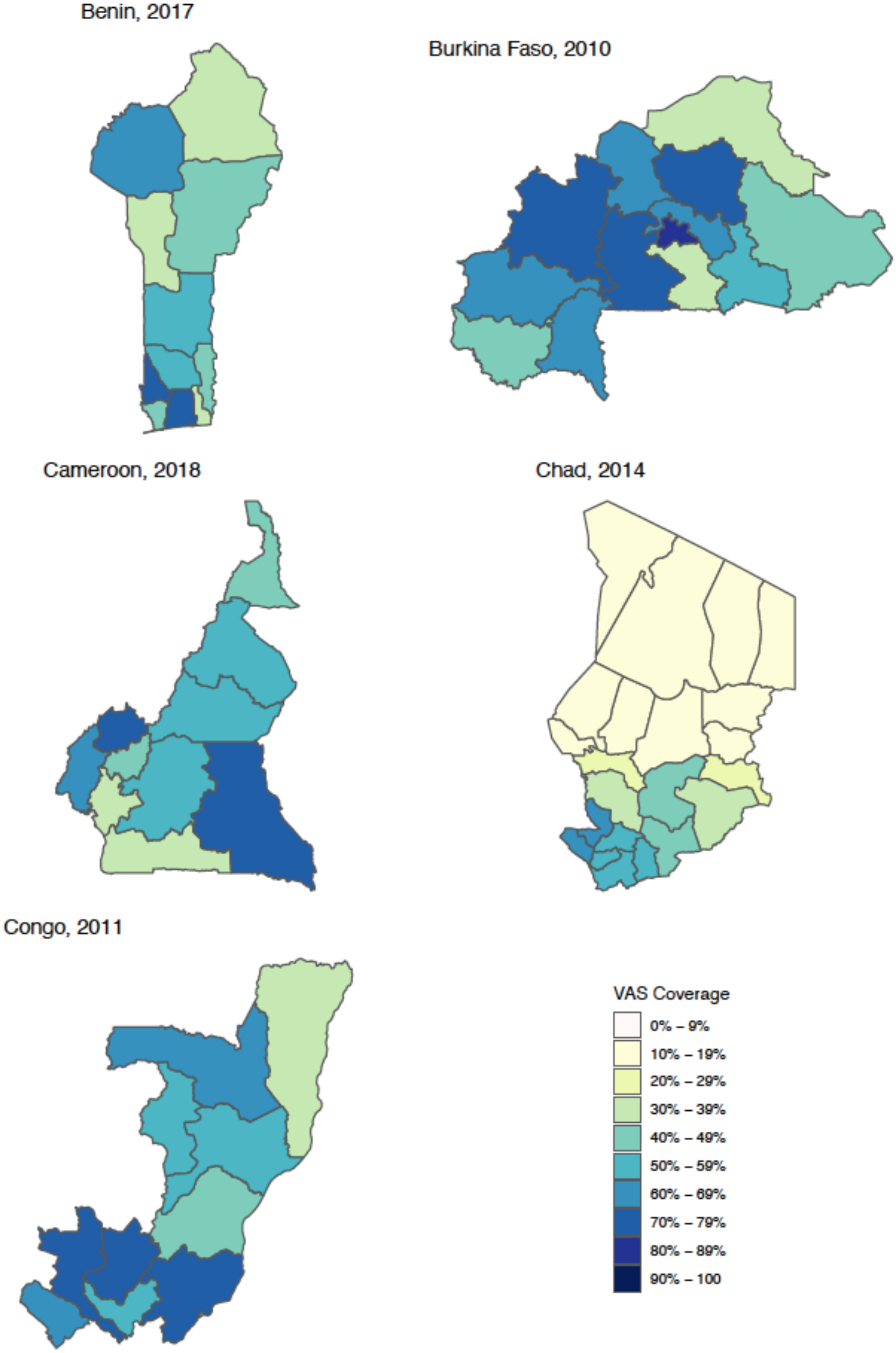

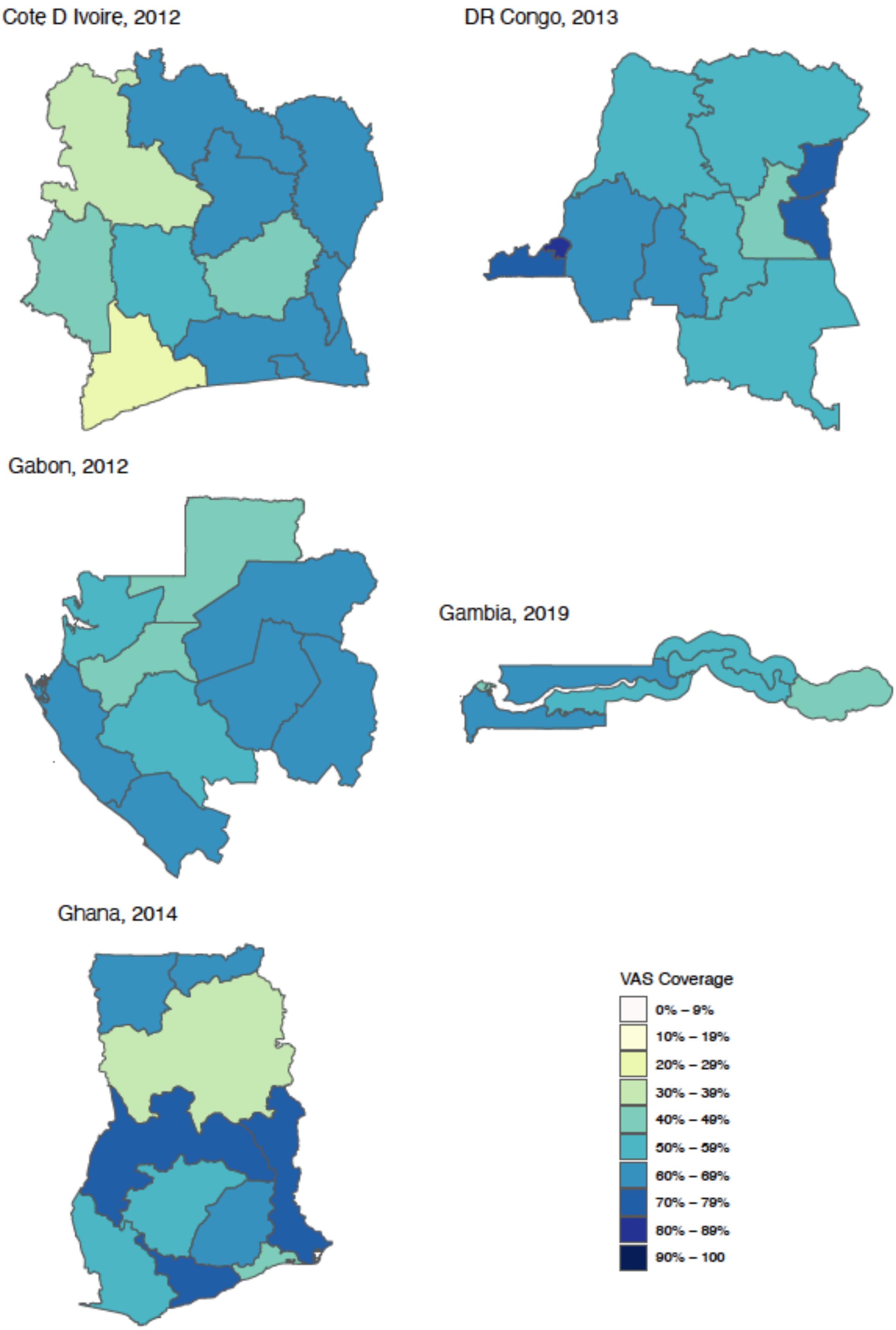
Cote D Ivoire, 2012
DR Congo, 2013
Gabon, 2012
Gambia, 2019
Ghana, 2014
VAS Coverage
0% – 9%
10% – 19%
20% – 29%
30% – 39%
40% – 49%
50% – 59%
60% – 69%
70% – 79%
80% – 89%
90% – 100

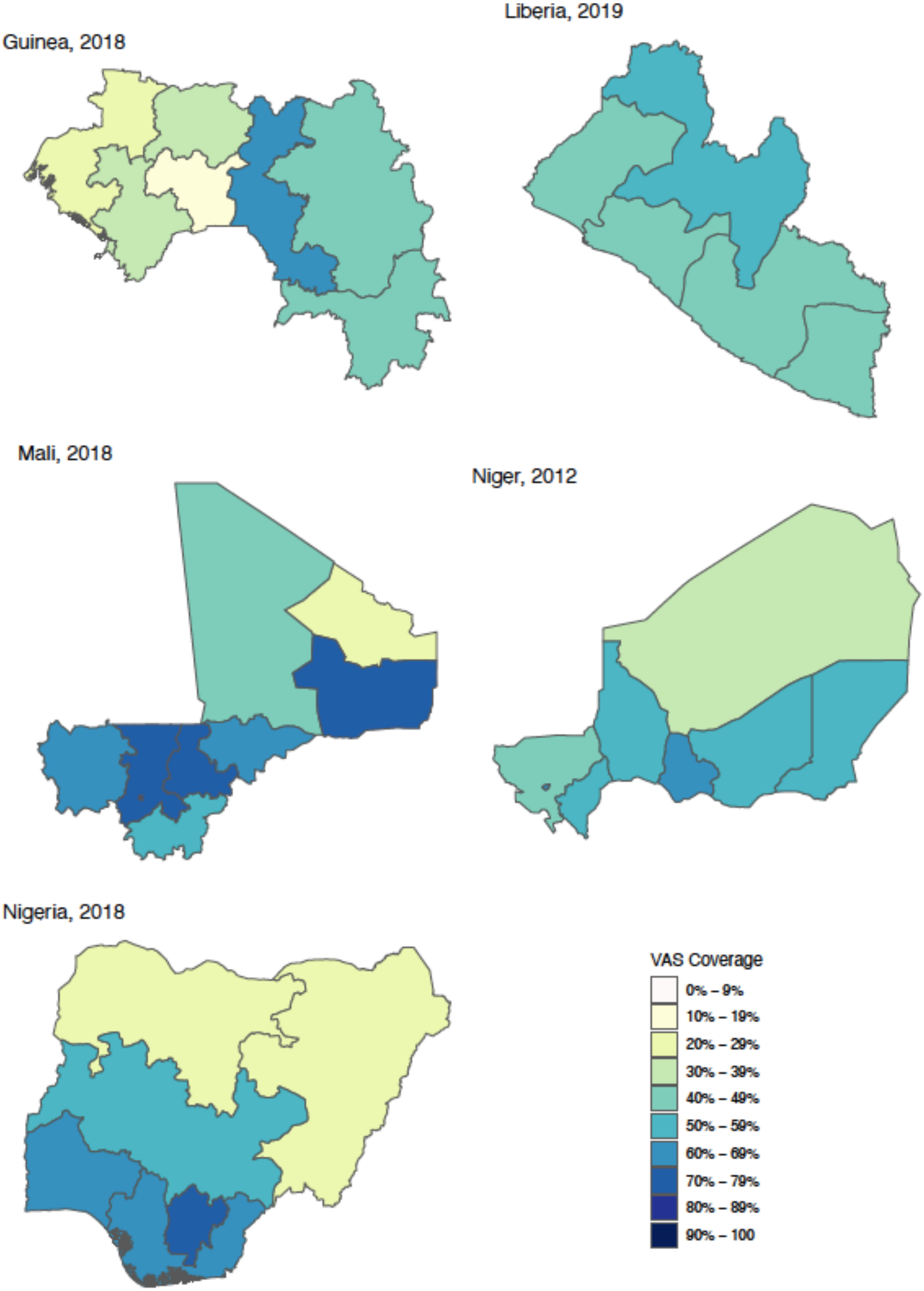
Guinea, 2018
Liberia, 2019
Mali, 2018
Niger, 2012
Nigeria, 2018
VAS Coverage
0% – 9%
10% – 19%
20% – 29%
30% – 39%
40% – 49%
50% – 59%
60% – 69%
70% – 79%
80% – 89%
90% – 100

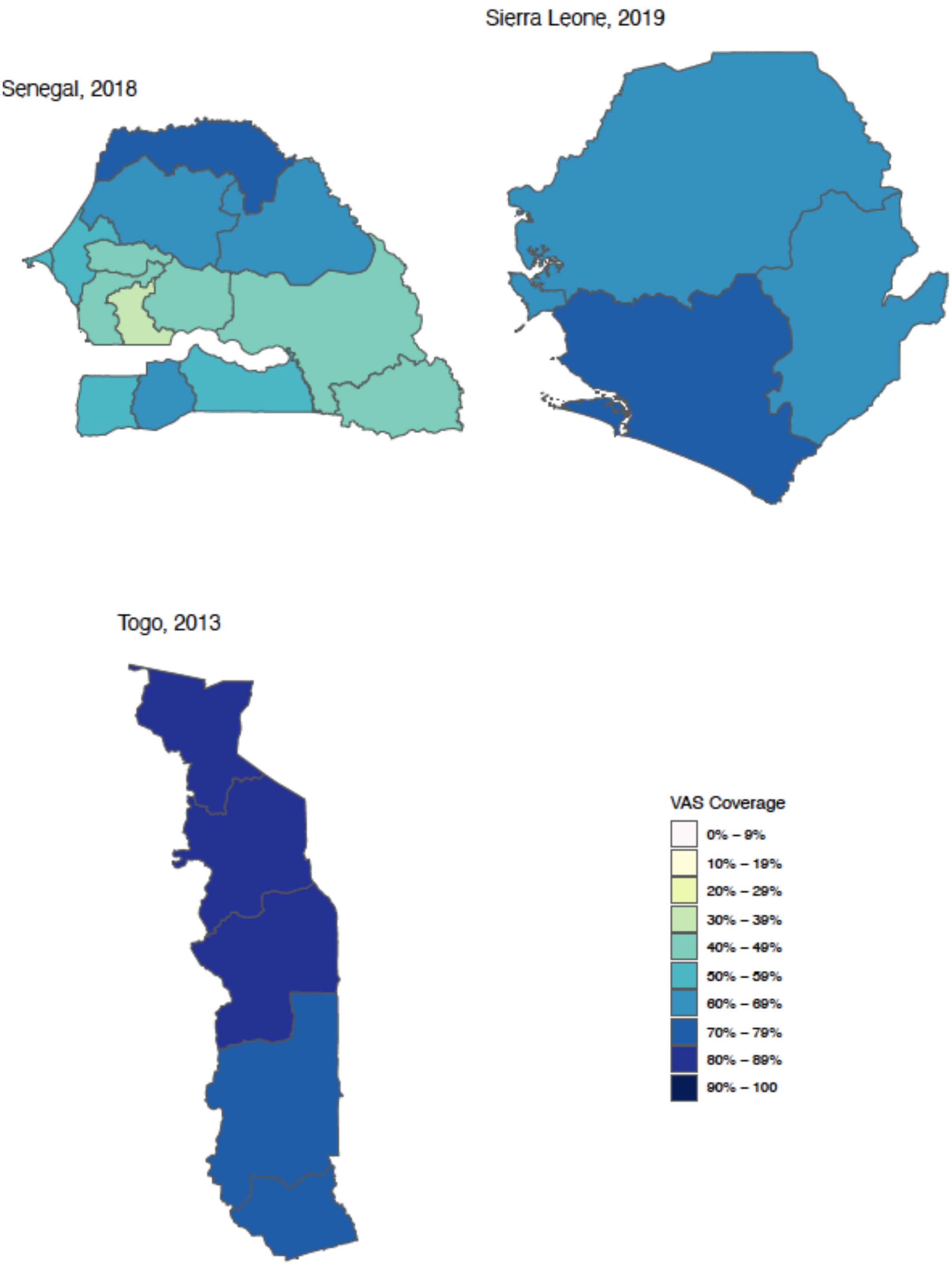

For peer review only - http://bmjopen.bmj.com/site/about/guidelines.xhtml

Supplementary Figure 3. Vitamin A supplementation (VAS) coverage between administrative regions for included countries in South Asia, East Asia, and the Western Pacific.

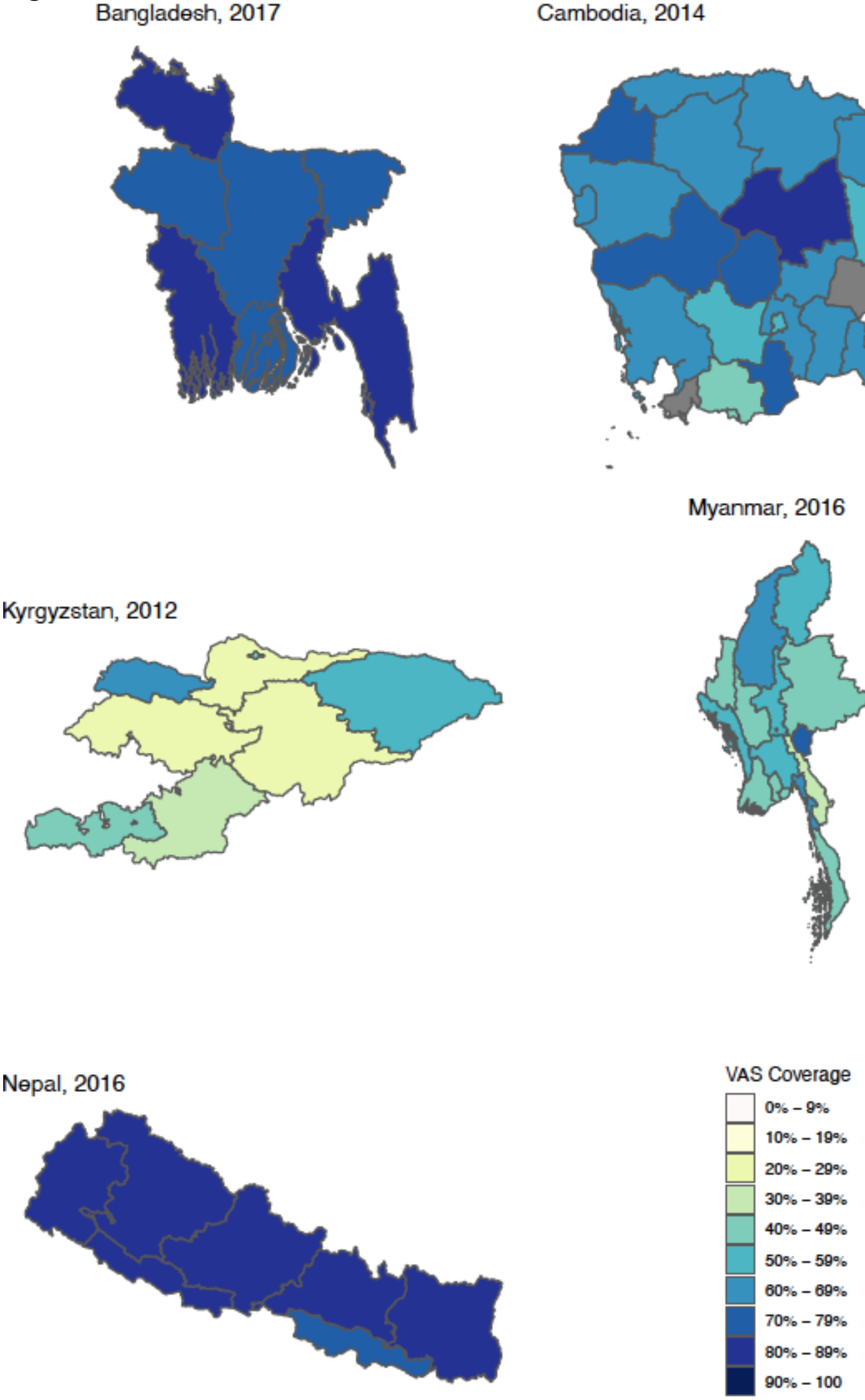

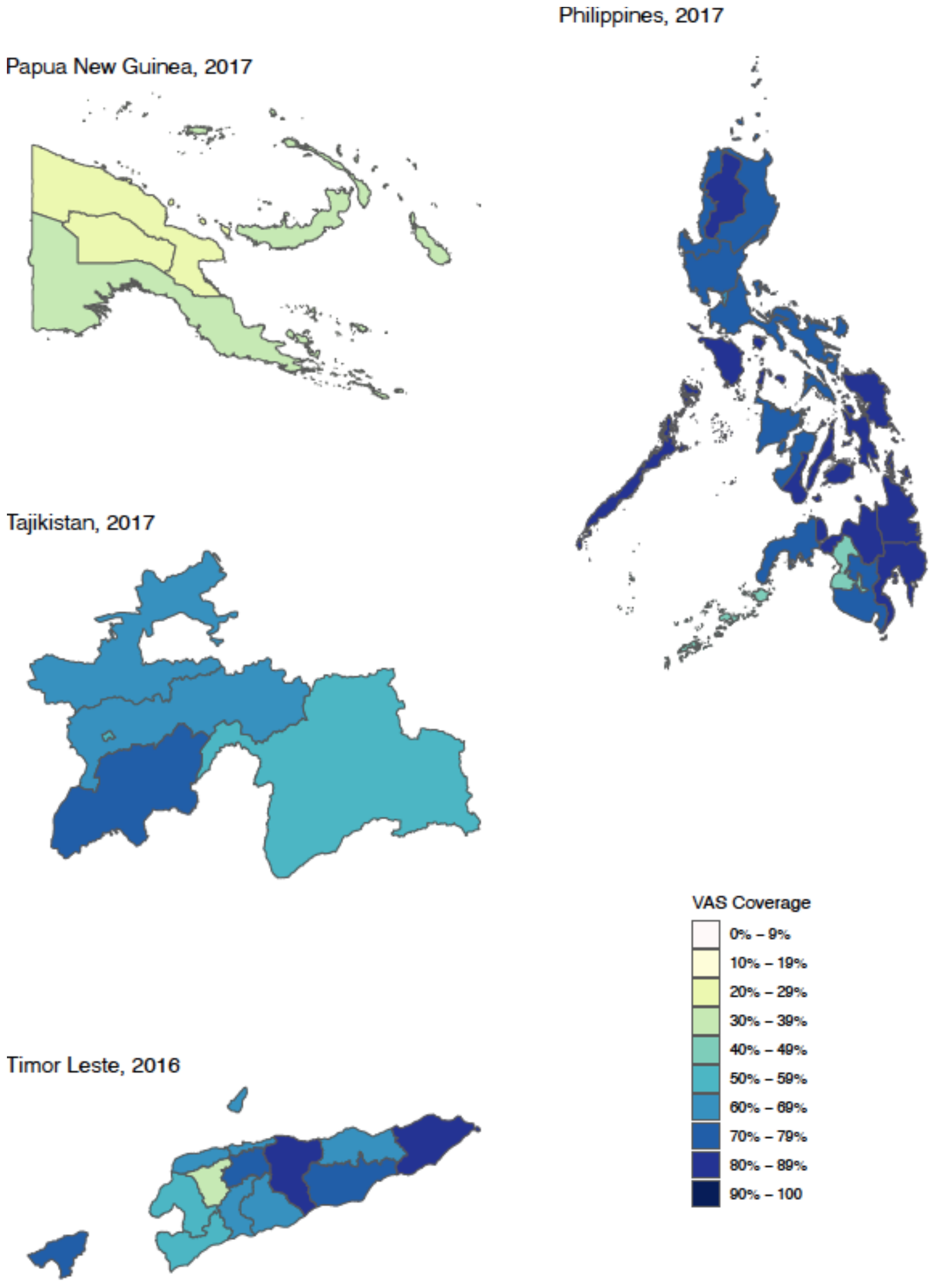

Philippines, 2017
Papua New Guinea, 2017
Tajikistan, 2017
Timor Leste, 2016
VAS Coverage
0% – 9%
10% – 19%
20% – 29%
30% – 39%
40% – 49%
50% – 59%
60% – 69%
70% – 79%
80% – 89%
90% – 100

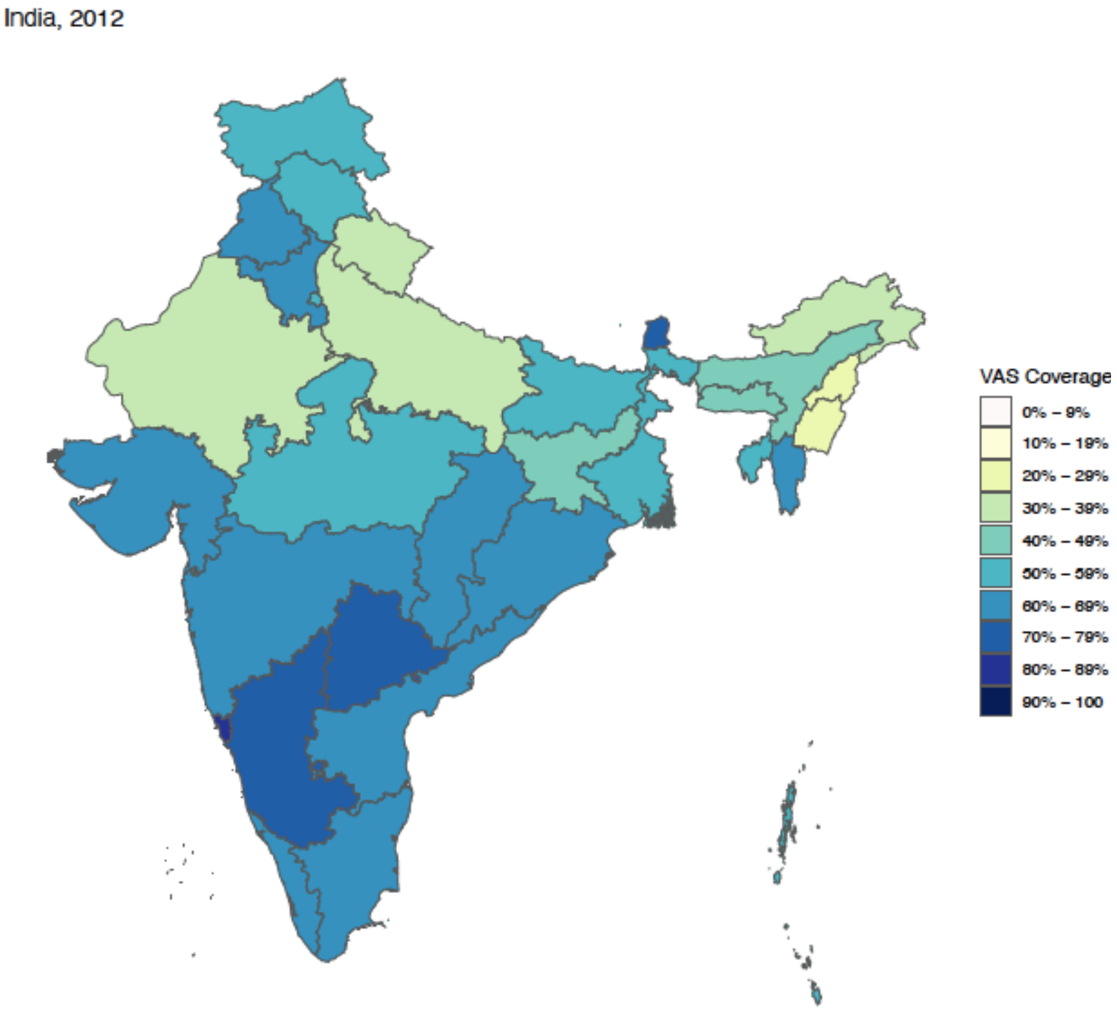
India, 2012
VAS Coverage
0% – 9%
10% – 19%
20% – 29%
30% – 39%
40% – 49%
50% – 59%
60% – 69%
70% – 79%
80% – 89%
90% – 100

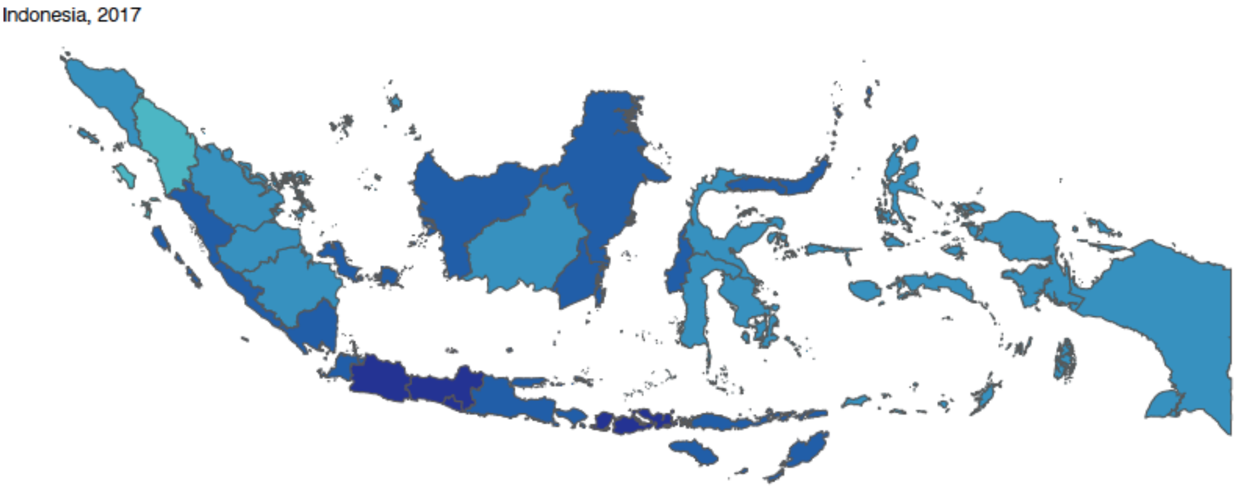
Indonesia, 2017

Supplementary Figure 4. Vitamin A supplementation (VAS) coverage between administrative regions for included countries in the Eastern Mediterranean Region.

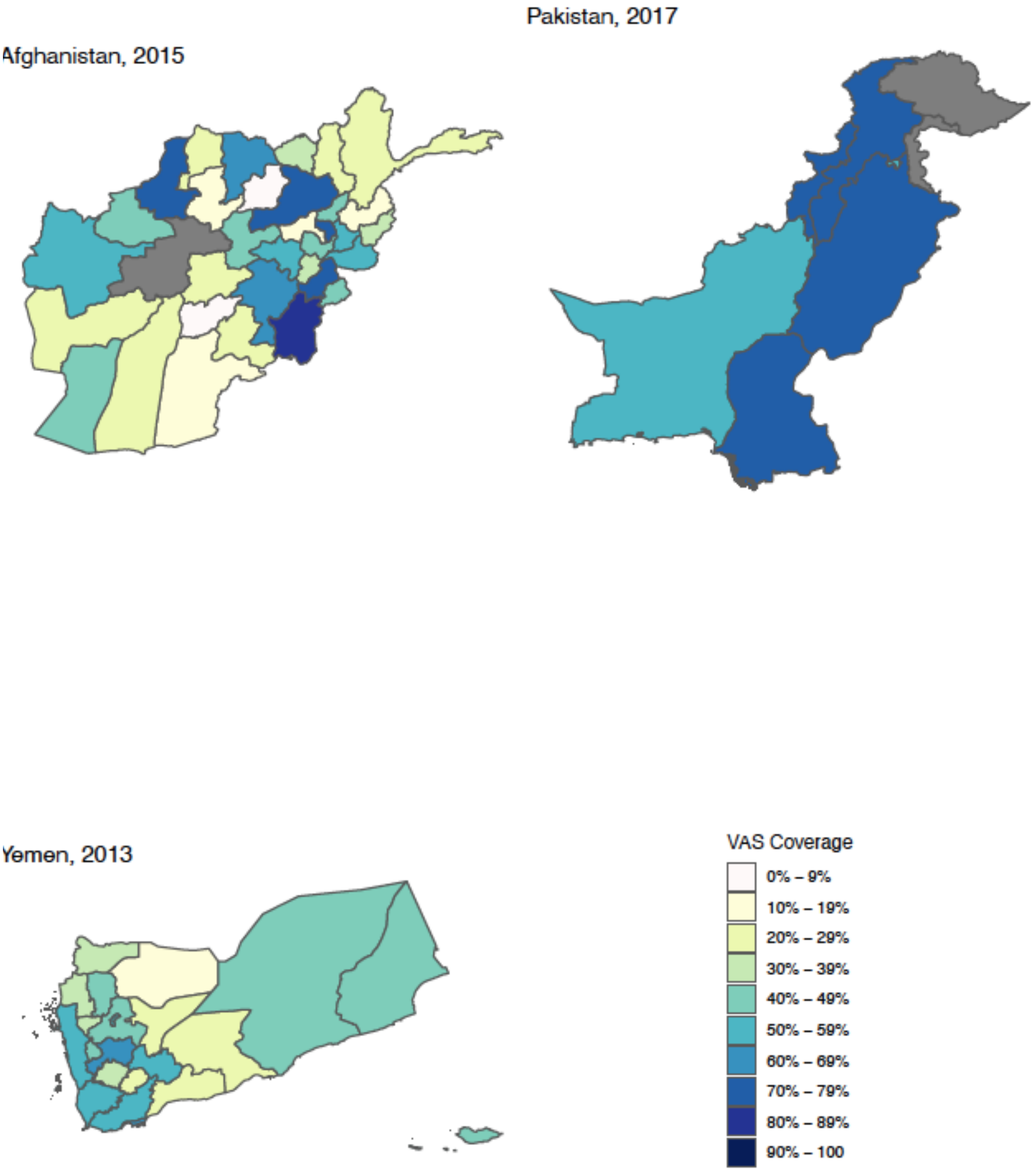

Supplementary Figure 5. Vitamin A supplementation (VAS) coverage between administrative regions for included countries in the Americas.

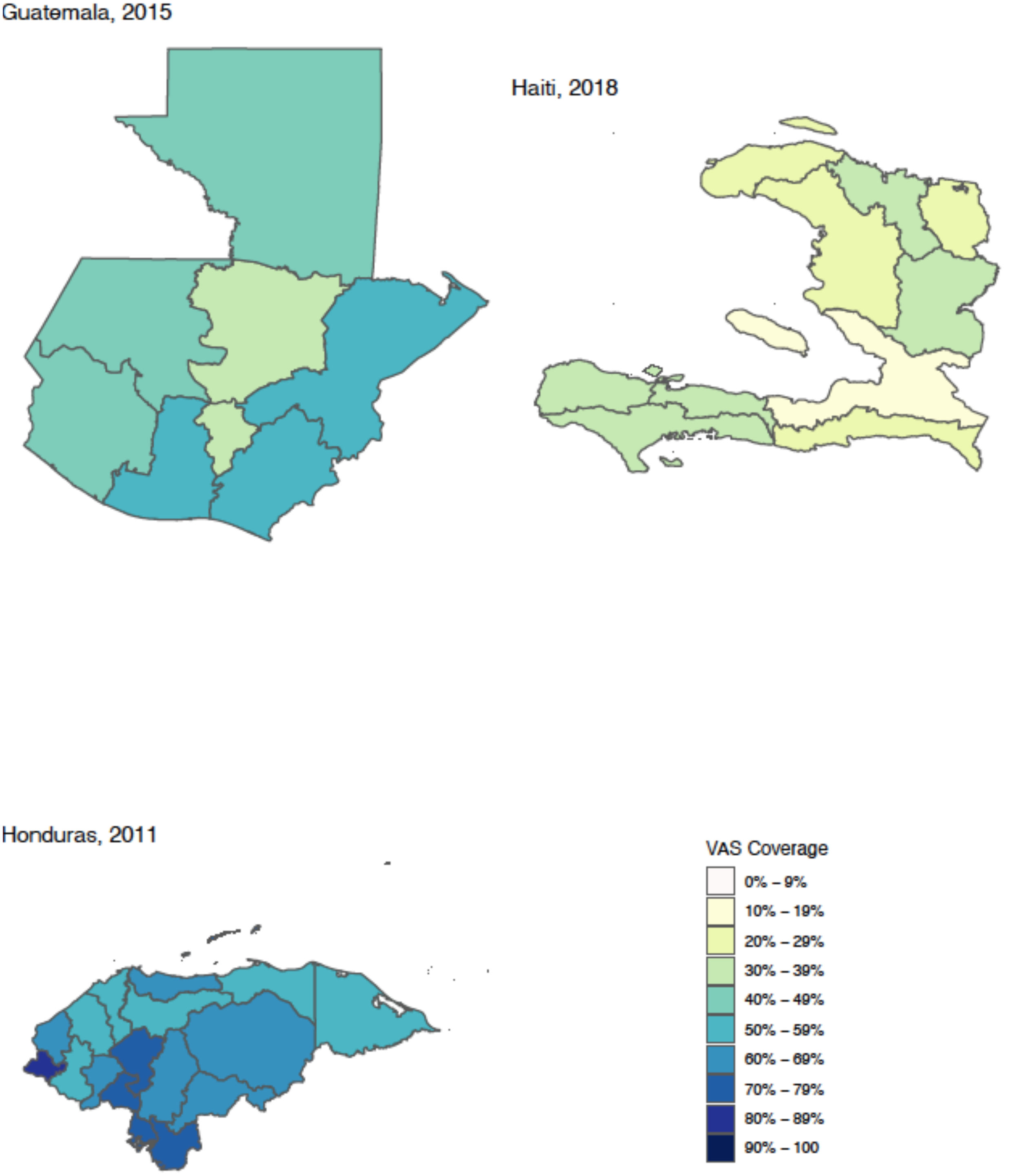

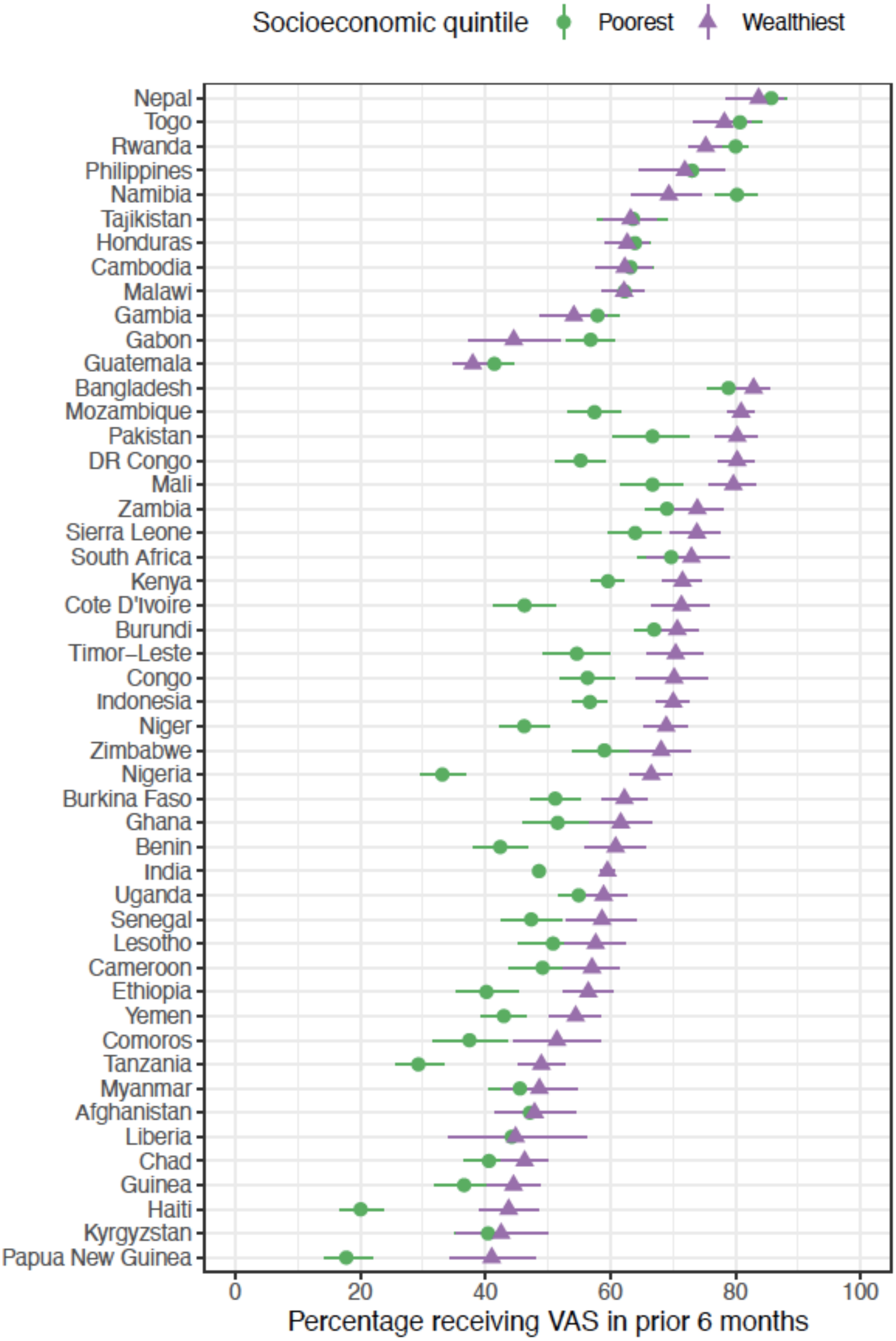

Supplementary Figure 6. Vitamin A supplementation (VAS) coverage among children from households of the poorest versus the richest socioeconomic quintiles.

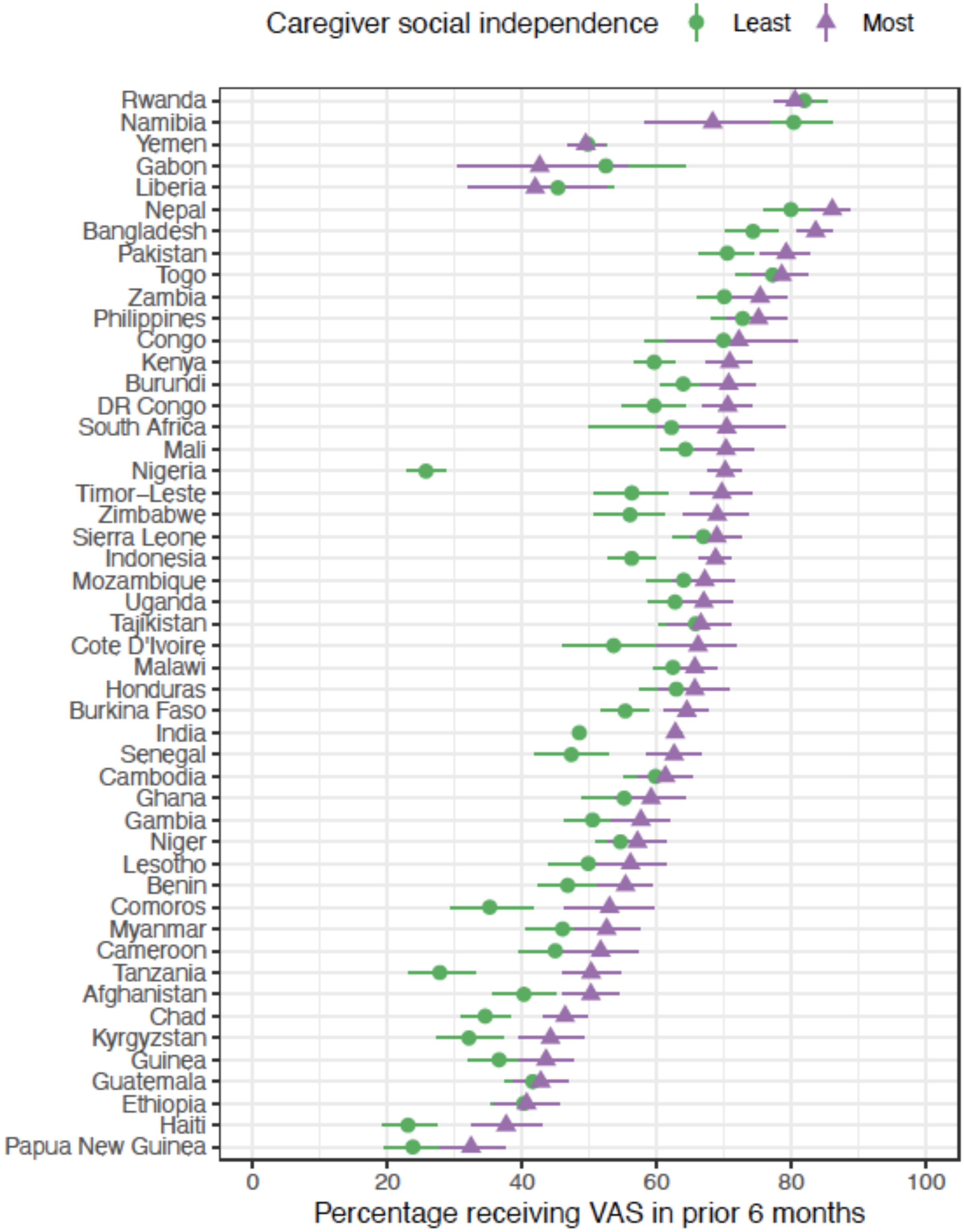

Supplementary Figure 7. Differences in vitamin A supplementation (VAS) coverage between children whose caregivers are the least versus the most socially independent in their country.

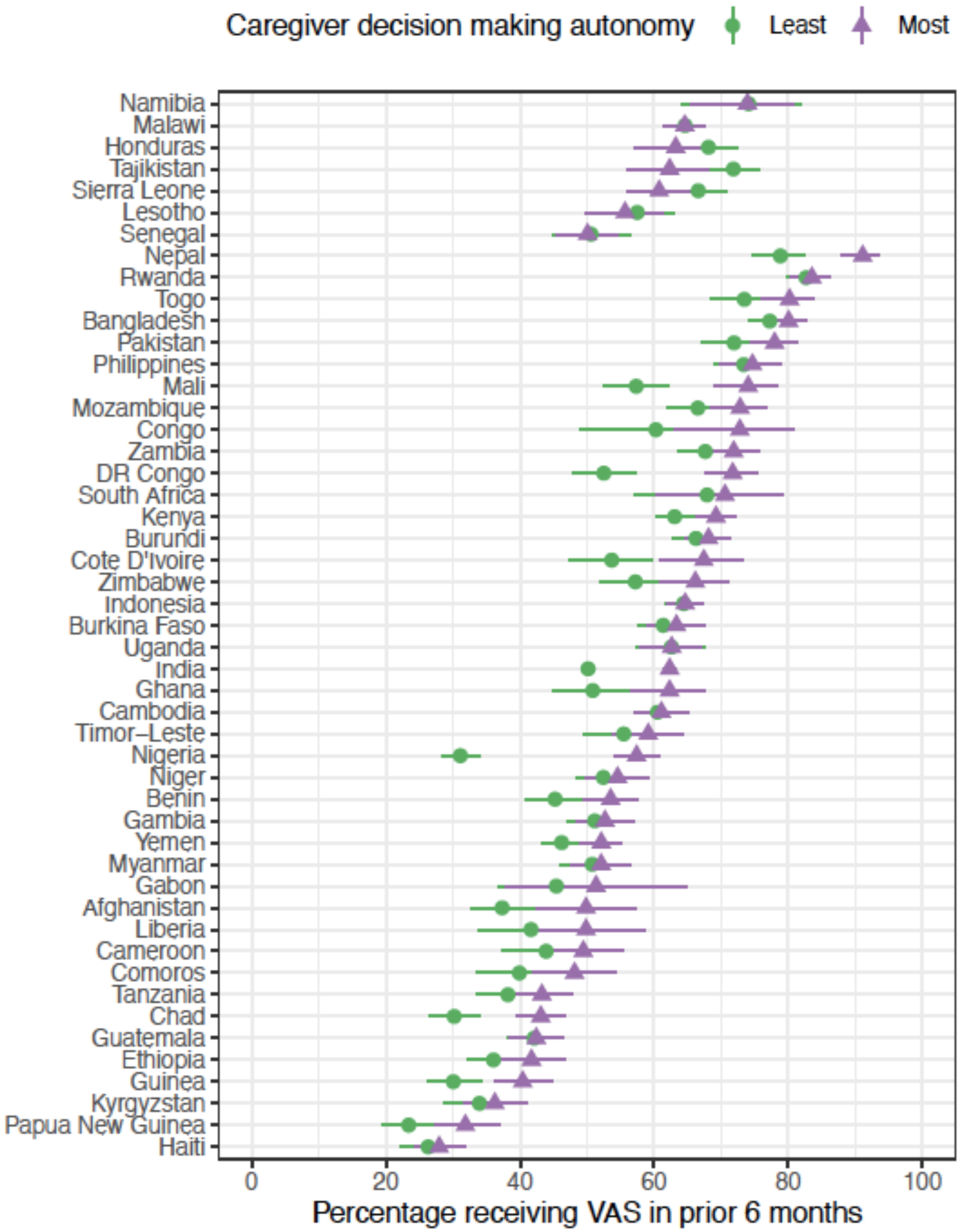

Supplementary Figure 8. Differences in vitamin A supplementation (VAS) coverage between children whose caregivers have the least versus the most decision-making autonomy in their country.

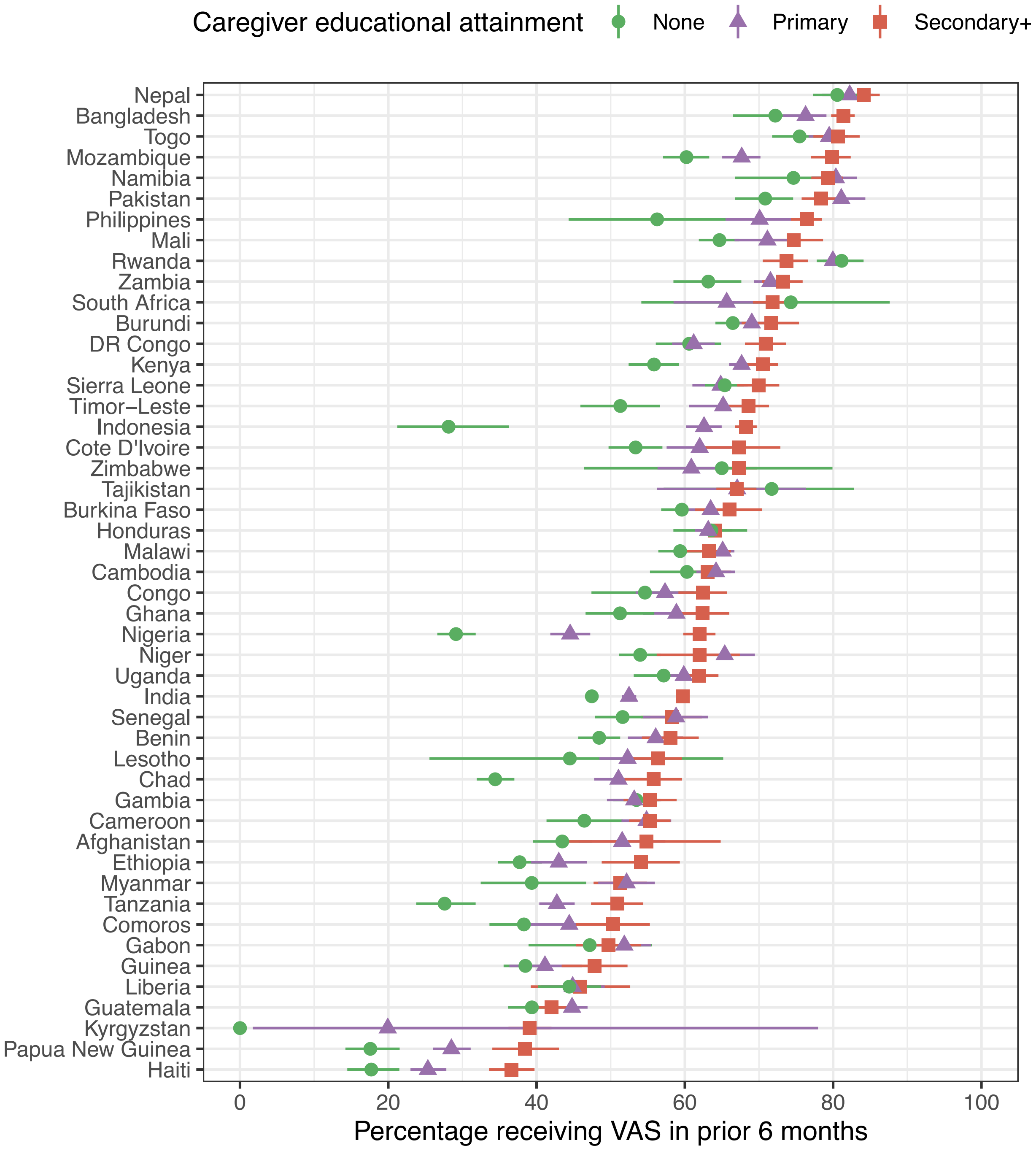

Supplementary Figure 9. Differences in vitamin A supplementation (VAS) coverage between children based on the educational attainment of the child's caregiver.

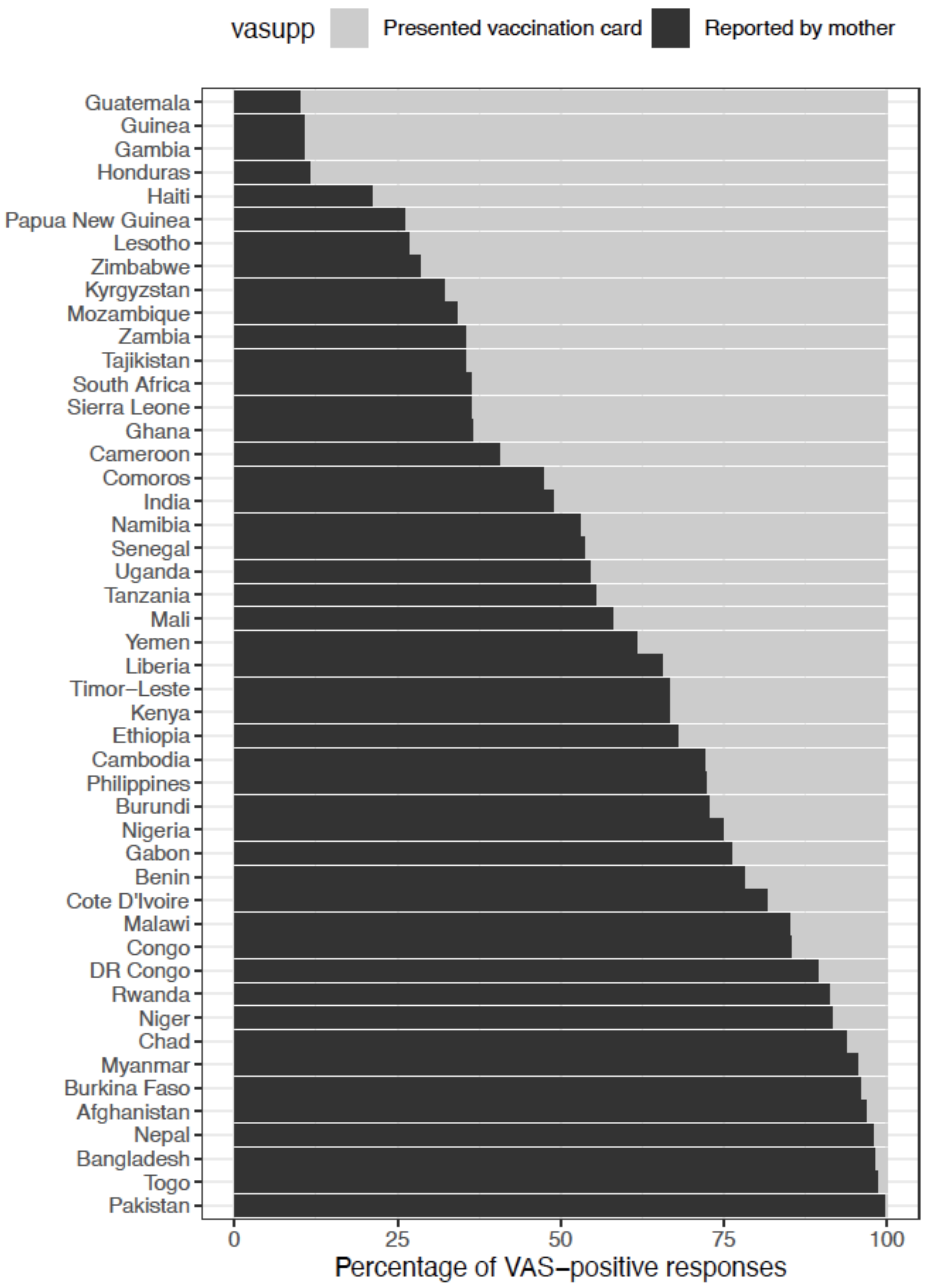

Supplementary Figure 10. Proportion of children who had received vitamin A supplementation (VAS) whose caregiver confirmed VAS reception with a vaccination card.

Evaluating equity dimensions of infant and child vitamin A supplementation programs using Demographic and Health Surveys from 49 countries

Journal:	*BMJ Open*
Manuscript ID	bmjopen-2022-062387.R1
Article Type:	Original research
Date Submitted by the Author:	01-Dec-2022
Complete List of Authors:	Tang, Kevin; London School of Hygiene & Tropical Medicine, Department of Population Health; UNICEF, Programme Division<br>Eilerts, Hallie; London School of Hygiene & Tropical Medicine, Department of Population Health<br>Imohe, Annette; UNICEF, Nutrition<br>Adams, Katherine; University of California Davis, Institute for Global Nutrition<br>Sandalinas, Fanny ; London School of Hygiene & Tropical Medicine, Department of Population Health<br>Joy, Edward; London School of Hygiene & Tropical Medicine, Department of Population Health<br>Moloney, Grainne; UNICEF, Programme Division<br>Hasman, Andreas; UNICEF, Nutrition
**Primary Subject Heading**:	Health policy
Secondary Subject Heading:	Paediatrics
Keywords:	Health policy < HEALTH SERVICES ADMINISTRATION & MANAGEMENT, Nutrition < TROPICAL MEDICINE, PAEDIATRICS, Public health < INFECTIOUS DISEASES, Geographical mapping < TROPICAL MEDICINE, International health services < HEALTH SERVICES ADMINISTRATION & MANAGEMENT

I, the Submitting Author has the right to grant and does grant on behalf of all authors of the Work (as defined in the below author licence), an exclusive licence and/or a non-exclusive licence for contributions from authors who are: i) UK Crown employees; ii) where BMJ has agreed a CC-BY licence shall apply, and/or iii) in accordance with the terms applicable for US Federal Government officers or employees acting as part of their official duties; on a worldwide, perpetual, irrevocable, royalty-free basis to BMJ Publishing Group Ltd ("BMJ") its licensees and where the relevant Journal is co-owned by BMJ to the co-owners of the Journal, to publish the Work in this journal and any other BMJ products and to exploit all rights, as set out in our licence.

The Submitting Author accepts and understands that any supply made under these terms is made by BMJ to the Submitting Author unless you are acting as an employee on behalf of your employer or a postgraduate student of an affiliated institution which is paying any applicable article publishing charge ("APC") for Open Access articles. Where the Submitting Author wishes to make the Work available on an Open Access basis (and intends to pay the relevant APC), the terms of reuse of such Open Access shall be governed by a Creative Commons licence – details of these licences and which Creative Commons licence will apply to this Work are set out in our licence referred to above.

Other than as permitted in any relevant BMJ Author's Self Archiving Policies, I confirm this Work has not been accepted for publication elsewhere, is not being considered for publication elsewhere and does not duplicate material already published. I confirm all authors consent to publication of this Work and authorise the granting of this licence.

For peer review only

Evaluating equity dimensions of infant and child vitamin A supplementation programs using Demographic and Health Surveys from 49 countries

Author names: Kevin Tang[1 2], Hallie Eilerts[1], Annette Imohe[2], Katherine P Adams[3], Fanny Sandalinas[1], Grainne Moloney[2], Edward JM Joy[1], Andreas Hasman[2]

Author affiliations:

[1] Department of Population Health, London School of Hygiene & Tropical Medicine, London, WC1E 7HT, United Kingdom

[2] UNICEF Programme Division, 3 United Nations Plaza, New York, NY 10017, United States

[3] Institute for Global Nutrition, University of California, Davis, One Shield Avenue, Davis, CA, 95616, United States

*Corresponding Author

Kevin Tang

Department of Population Health

London School of Hygiene & Tropical Medicine

Keppel Street

London, WC1E 7HT, United Kingdom

P: (+44) 20 7636 8636

F: (+44) 20 7436 5389

Email: kevin.tang1@lshtm.ac.uk

For peer review only

For peer review only - http://bmjopen.bmj.com/site/about/guidelines.xhtml

ABSTRACT (269/300 words max)

Objectives: Vitamin A deficiency affects an estimated 29% of all children under five years of age in low- and middle-income countries, contributing to child mortality and exacerbating severity of infections. Bi-annual vitamin A supplementation (VAS) for children aged 6 – 59 months can be a low-cost intervention to meet vitamin A needs. This study aimed to present a framework for evaluating the equity dimensions of national VAS programs according to determinants known to affect child nutrition and assist programming by highlighting geographic variation in coverage.

Methods: We used open-source data from the Demographic and Health Survey for 49 countries to identify differences in VAS coverage between sub-populations characterized by various immediate, underlying, and enabling determinants of vitamin A status and geographically. This included recent consumption of vitamin A-rich foods, access to health systems and services, administrative region of the country, place of residence (rural vs urban), socioeconomic position, caregiver educational attainment, and caregiver empowerment.

Results: Children who did not recently consume vitamin A-rich foods and who had poorer access to health systems and services were less likely to receive VAS in most countries despite potentially having a greater vitamin A need. Differences in coverage were also observed when disaggregated by administrative regions (88% of countries) and urban vs rural residence (35% of countries). Differences in vitamin A coverage between sub-populations characterized by other determinants of vitamin A status varied considerably between countries.

For peer review only

Conclusion: VAS programs are unable to reach all eligible infants and children, and sub-population differences in VAS coverage characterized by various determinants of vitamin A status suggest that VAS programs may not be operating equitably in many countries.

For peer review only

STRENGTHS AND LIMITATIONS

Strengths

- We used Demographic and Health Survey (DHS) data from 49 low- and middle-income countries to conduct a standardized analysis of the equity dimensions of vitamin A supplementation (VAS) coverage, where consistencies in findings could be broadly applied to inform global VAS policy.
- The covariates included in this study, guided by the 2020 UNICEF conceptual framework, covered a diverse range of social and geographic determinants with relevance to equitable VAS programming.

Limitations

- The question specific to VAS in the DHS questionnaire is dependent on a multiple month recall by the caregiver of the child, which is subject to recall bias.
- In settings where VAS is delivered via biannual campaigns, DHS VAS coverage may be underestimated if the timing of the survey is planned immediately before a campaign.

For peer review only

INTRODUCTION

The UNICEF Nutrition Strategy 2020 – 2030 commits to a goal of protecting and promoting diets, services, and practices that support optimal nutrition, growth, and development for all children to end child malnutrition in all its forms [1]. Central to achieving this goal requires recognizing the interaction between five core systems (food, health, water & sanitation, education, social protection) to improve nutrition outcomes. However, a strategy focused on working within established systems may exacerbate unequal nutrition outcomes rooted in deeper inequities that arise from already inequitable systems [2]. Therefore, when implementing policies to ensure the most vulnerable have access to services, governments should conduct regular reviews of coverage of programs to allow for reorientation as needed.

Vitamin A deficiency affects an estimated 29% of all children under five years of age in low- and middle-income countries [3], contributing to child mortality and disease burden through direct clinical manifestation (e.g., xerophthalmia) and susceptibility to and exacerbated adverse outcomes from infection (e.g., measles, diarrhea) [4]. For children with vitamin A deficiency or who have an increased risk of mortality, high-dose vitamin A supplementation (VAS) can be a low-cost intervention to meet vitamin A needs when delivered using infrastructure from existing community-based delivery programs [5]. Following an exhaustive review of the current evidence exploring the effect of VAS in preventing morbidity and mortality in infants and children [4,6], in 2011 the World Health Organization (WHO) confirmed a recommendation for infants and children 6 – 59 months of age to receive a dose of VAS every four to six months in contexts where vitamin A deficiency is a public health problem [7]. Although the past two

For peer review only

decades have seen increases in global VAS coverage [8], stagnating VAS coverage in recent years brings into question whether current universal delivery strategies are consistently missing hard-to-reach children most in need, especially in settings with low coverage [9]. This is particularly the case as VAS programs move away from delivery in campaigns towards routine delivery [8].

The delivery of VAS can be integrated into routine community-based health service delivery programs. In many countries, community-based delivery programs, such as the national Essential Programme on Immunization (EPI), offer the most consistent contacts between the youngest children and the health system, thus creating a platform for the large-scale administration of VAS. However, in contrast to VAS administration protocols, childhood immunization programs typically benefit only children up to one year of age when the last vaccine dose is administered, and few countries have a contact point for immunization at six months. Moreover, multi-country analyses of diphtheria-tetanus-pertussis (DTP) and measles containing vaccines (MCV) identified gaps in program coverage between geographic and socioeconomic subpopulations [10,11]. All together, these differences suggest that the services these community-based delivery programs provide are not equitably accessible to children with the greatest needs and may affect the VAS programs that depend on these services for delivery.

Global guidance recommends using "two-dose coverage" as a metric for advocacy to promote national and global progress towards achieving universal VAS coverage [12]. In this context, "coverage" is estimated as the nationally aggregated number of VAS doses administered in a

For peer review only - http://bmjopen.bmj.com/site/about/guidelines.xhtml

country over a six-month period (also referred to as semester) from administrative records divided by the estimated number of children ages 6 – 59 months in that country for that specific semester. “Two-dose coverage” is thus established on an annual basis as the semester in a given year with lower coverage. Two-dose coverage increases the feasibility of collating national VAS program data bi-annually, which is advantageous for advocacy and other such uses. From the programmatic perspective, both two-dose coverage and single semester coverage are limited in identifying differences in coverage among sub-populations within a country. To evaluate whether national VAS programs are missing the children with the greatest needs, global guidance recommends using indicators generated from subnational data that are readily available, easy to understand, and relevant to the information needs of program managers [13].

There is a significant information gap in evaluating the equity dimensions of nutrition interventions, or the extent to which a community-based nutrition program is reaching the children most in need [2]. Nationally representative household surveys, such as the Demographic and Health Surveys (DHS), can potentially provide insight into the equity dimensions of program delivery. Since 1985, the DHS program has conducted nationally-representative cross-sectional surveys in over 90 predominantly low- and middle-income countries, producing detailed, cross-sectional data on a variety of indicators describing demographics, health, economics, and social welfare [14]. DHS questionnaires and variable nomenclature are consistent between all countries in the DHS program, facilitating rapid analysis across multiple countries and survey years. DHS microdata enable the exploration of a

For peer review only - http://bmjopen.bmj.com/site/about/guidelines.xhtml
For peer review only

variety of indicators to measure population health outcomes and the mechanisms influencing them in great depth.

DHS collect detailed information on the reception of VAS by infants and children, however using such data to inform national VAS programs is not straightforward. The aim of this study was to evaluating the equity dimensions of national VAS programs according to determinants known to affect child nutrition and geographically using DHS data from multiple countries.

For peer review only

134 METHODS

135 ***Demographic and Health Surveys***

136 This was a secondary data analysis using data collected as part of the DHS from various
137 countries [15]. We reviewed DHS data from 64 countries that have been prioritized by UNICEF
138 for support in their current national VAS programming efforts and ultimately included 49
139 countries in which a complete DHS survey was conducted since 2010, using the most recent
140 data in countries with multiple surveys. The analysis depended on data from the Woman's
141 Questionnaire, which contains information on the survival status of the children born to the
142 respondent, and more detailed information on children born in the last five years, such as
143 vaccination history, breastfeeding practice, recent illness, and anthropometry.

144

145 ***Sampling Strategy & Study Population***

146 The DHS protocol employs a two-stage sampling procedure [16]. In the first stage, enumeration
147 areas are defined geographically (stratified by urban/rural residence and administrative region)
148 using the country's most recent population census to establish the survey's primary sampling
149 unit. In the second stage, systematic sampling identifies 20 – 30 households for inclusion,
150 where selected households are visited by trained interviewers who administer the Woman's
151 Questionnaire to women of reproductive age (15-49 years).

152

153 Our analysis included all children aged 6 – 59 months with available VAS history data. VAS
154 program participation is probed in the DHS questionnaire's *vaccination history section* by asking
155 caregivers of children whether her child (or children) aged 6 – 59 months has (or have) received

For peer review only

a vitamin A dose in the last six months [17]. If possible, caregivers are asked to present proof of VAS reception using a vaccination history card, and if not available, are asked to recall VAS administration from memory after probing the caregivers to describe post-natal care activities in which her and each child have participated [18]. The primary outcome of this study was defined as the percentage of children receiving VAS in the prior six months, or more simply, "VAS coverage".

Equity and Geographic Covariates

We stratified the primary outcome by several covariates describing various dimensions of equity, which were selected in accordance with the 2020 UNICEF Conceptual Framework on the Determinants of Maternal and Child Nutrition [1]. The framework used in this analysis was developed to guide nutrition programming by outlining three levels of determinants (i.e., immediate, underlying, enabling) that contribute to preventing malnutrition in all its forms. By using this framework to guide covariate selection, this study recognizes the levels and interconnectedness of various determinants affecting vitamin A status to define the underlying systems and processes that affect children with the greatest vitamin A needs. Geographic covariates were also selected as targeting geographically defined populations is more operationally feasible.

Covariates used to represent immediate and underlying child nutrition determinants included recent consumption of vitamin A-rich foods and access to healthcare systems and services. Recent consumption of vitamin A-rich foods from the DHS questionnaire is only collected for

For peer review only

children aged 6 – 23 months, so this indicator refers predominantly to children who are within the age range where breastfeeding is recommended [19]. Access to healthcare systems and services is measured using the proxy variable of vaccine reception. The first dose of the DTP vaccine (administered at six weeks of age) is one of the first vaccines given to an infant, where children who do not receive it are expected to have the lowest access to healthcare systems and services. Reception of the first dose of MCV (administered at nine months of age) is expected to be delivered via similar platforms to VAS through both routine provisions and campaigns. Covariates serving as indicators for enabling child nutrition determinants included socioeconomic position, the caregiver's educational attainment, and two women's empowerment dimensions calculated according to the Survey-based Women's Empowerment (SWPER) index [20], which measure the caregiver's social independence and decision-making autonomy. Geographic covariates included urban vs rural residence and administrative regions specific to each country. Descriptions of all covariates are available in Table 1.

For peer review only

For peer review only - http://bmjopen.bmj.com/site/about/guidelines.xhtml

Table 1. Description of study covariates used to represent immediate, underlying, and enabling determinants of child nutrition and geography

Determinant	Type	Coding details
Recent consumption of vitamin A-rich foods	Immediate/ Underlying	Whether the following types of food were reported being consumed by the child (aged 6-23 months) in the 24 hours prior to the interview: eggs, any meat, fish, orange-fleshed pumpkin/carrot/squash/sweet potato, vitamin A-rich fruit (mango, papaya), liver/heart/organ meat, dark-leafy-green vegetables
Access to healthcare services & systems (DTP1 vaccine)	Immediate/ Underlying	Whether the child received the first dose of the diphtheria, tetanus, and pertussis (DTP1) vaccine, administered at 6 weeks of age per international guidelines
Access to healthcare services & systems (MCV1 vaccine)	Immediate/ Underlying	Whether the child received the first dose of the measles containing vaccine (MCV1), administered at 9 months of age per international guidelines
Administrative region	Geography	Administrative region the household is located, defined by the sub-national reporting area (provinces or groups of provinces) as defined by the DHS recode
Place of residence	Geography	Whether household is in an urban or rural location
Socioeconomic position	Enabling	Quintile of household wealth defined using a principal component analysis score comprised of household living conditions and durable assets (Poorest = 1st quintile, Wealthiest = 5th quintile)
Caregiver's social independence	Enabling	Women's empowerment indicator calculated as quintiles of SWPER index. Social independence domain included data related to education, frequency of reading newspapers/magazines, and age at first childbirth and at first cohabitation (includes children with partnered caregivers) (Least socially independent = 1st quintile, Most socially independent = 5th quintile)
Caregiver's decision-making autonomy	Enabling	Women's empowerment indicator calculated as quintiles of SWPER index. Decision-making autonomy domain included questions about involvement in household decisions and whether the respondent worked in the past 12 months (includes children with partnered caregivers) (Least decision-making autonomy = 1st quintile, Most decision-making autonomy = 5th quintile)
Caregiver's educational attainment	Enabling	Highest educational attainment of caregiver (None, primary school, secondary school or higher)

Statistical Analysis

Percentage of children receiving VAS in the prior six months was stratified by each equity covariate independently for every included country. All estimates were adjusted using population sample weights provided by the DHS Program [21]. Percentages of children receiving VAS in each stratum in the prior six months were presented with 95% confidence intervals (95%CI), where statistically significant differences in VAS coverage were identified as those with non-overlapping 95%CIs. VAS coverage by administrative region was mapped using shapefiles downloaded from GADM (version 3.6) [22].

Data analysis was conducted in RStudio (version 3.6.1, the R Foundation for Statistical Computing). Data was accessed through the DHS program's application programming interface (API) on 23 November 2022 via the functions available on the *rdhs* package [23]. Survey weight adjustments and statistical analyses were conducted using the functions available on the *survey* and *srvyr* packages [24] and additional data cleaning and management used a variety of functions from the *tidyverse* package [25].

Patient and Public Involvement

As a secondary analysis of publicly available, de-identified data collected as part of the DHS, patients and the public that were included as part of the study population were not involved in the design of this study.

For peer review only

RESULTS

Summary of included surveys & populations

In total, the birth history records of 1,465,369 women, corresponding to 608,388 children 6-59 months, were included in this study. For the survey question asking about VAS reception within the prior six month, the rate of response was >90% for all countries included. A summary of all included countries and the DHS data used in this study is available in Supplementary Table 1.

The national VAS coverage mean among included countries was 58% and ranged from 28% to 83%, where countries with the lowest national VAS coverage were Papua New Guinea (28%), Haiti (28%), Kyrgyzstan (39%) and Guinea (40%). The percentage of children who did not recently consume vitamin A-rich foods among included countries ranged from 19% to 74%, where countries with the lowest percentage of consumption were Burkina Faso (74%), Niger (73%), and Ethiopia (70%). For vaccinations, the percentage of non-vaccinated children among included countries ranged from 1% to 48% for DTP1 and 13% to 69% for MCV1. A descriptive summary of the survey population of children aged 6-59 months for each country is provided in Supplementary Table 2.

VAS coverage by immediate & underlying determinants

In figures 1 and 2, we present VAS coverage for all countries stratified by recent consumption of vitamin A-rich foods and access to healthcare systems and services. VAS coverage was significantly higher among children who recently consumed vitamin A-rich foods, compared to children who did not in 85% (n=41) of countries (Figure 1). For access to healthcare systems and

For peer review only - http://bmjopen.bmj.com/site/about/guidelines.xhtml

services, VAS coverage was significantly higher in children who had access to their first dose of the DTP vaccine in 47 of the 49 countries compared to those who did not have access to these vaccines (Figure 2a). VAS coverage was significantly higher in children who had access to their first dose of MCV in all 49 countries (Figure 2b).

VAS coverage by geography and other enabling determinants

In Figure 3, VAS coverage is stratified by geographic covariates of vitamin A nutritional status for all countries. For place of residence, 31% of countries (n=15) had significantly lower VAS coverage in populations residing in rural versus urban areas. In countries where VAS coverage in rural residences exceeded that of urban residences, differences in coverage were never >8%. For administrative region, regions with the lowest and highest VAS coverage in each country had significant differences in 88% of countries (n=43) with spatial structure in sub-national VAS coverage visible when mapped (Figure 4 for Chad, India, Nigeria, Ethiopia, Yemen; full set of country maps available in Supplementary Figure 1-5).

In 73% of countries (n=36), VAS coverage was significantly higher in the wealthiest quintile of the population compared to the poorest quintile of the population, where the differences in coverage between poorest and wealthiest were greatest in Nigeria (33% difference), Cote D'Ivoire (25% difference), and the Democratic Republic of the Congo (25% difference) (Supplementary Figure 6). In 37% of countries (n=18), children of caregivers who were the most socially independent had significantly higher VAS coverage compared to caregivers who were the least socially independent (Supplementary Figure 7). In 20% of countries (n=10), VAS

For peer review only - http://bmjopen.bmj.com/site/about/guidelines.xhtml

coverage in children of caregivers who were most autonomous in their decision-making was significantly higher compared to children of the least autonomous caregivers (Supplementary Figure 8). Caregivers with higher educational attainment had higher VAS coverage for their children compared to caregivers with lower educational attainment in several countries (Supplementary Figure 9).

For peer review only

DISCUSSION

This study used open-source data to identify inequities in VAS program coverage so that strategies can be devised to improve VAS coverage among unreached populations. We found that children who likely have lower access to vitamin A-rich foods and who have impaired access to healthcare systems and services are also less likely to receive VAS. This pattern was consistent across most countries and highlights the challenges current VAS delivery faces to reach children that are likely most in need of VAS. The analysis also shows that in some countries, children missed by VAS also reside in the poorest households, in rural areas, and have caregivers who are more constrained by gender norms. Although these are the general trends, we also observed considerable variation between countries. While the use of DHS data to gain insight into VAS programs has been conducted in some countries [26–28], future analyses using DHS data can help inform VAS program operations in other country contexts. National programs can use the analysis presented here as an advocacy tool for universal coverage, or as a framework to improve targeting and prioritization of children who are likely to be most in need of VAS delivery programs.

The demonstrated use of DHS data to provide sub-national equity perspectives can provide useful VAS program insights. For example, this study indicated that lower VAS coverage for children who may have lower access to vitamin A-rich foods and healthcare systems and services was consistent across most countries. According to global estimates using UNICEF infant and young child feeding data, the consumption of vitamin A-rich foods by complementary feeding children is lowest in poorer and more rural children [29] suggesting

For peer review only

that in combination multiple enabling determinants affecting nutrition are likely to contribute to reduced access to vitamin A-rich foods. However, this study suggests that in many countries, VAS coverage is either equal or lower in these rural and poorest populations despite likely having greater need for VAS.

To address these gaps, VAS programs may benefit from being more aligned with routine community-based delivery programs. In children aged 6 – 11 months, in countries where VAS coverage between vaccinated and unvaccinated children diverges, there is a potential opportunity to improve VAS coverage by aligning programs more closely to the country's EPI systems. This alignment with EPI systems will require program reforms, including the creation of a six month contact point in routine systems, inclusion of VAS dosing schedules on child health cards [30], and strengthening supply chains for vaccines and vitamin A capsules to ensure concomitant availability. For older children who are not serviced by the EPI (12 – 59 months), there may be other established routine early childhood programs where the routine delivery of VAS could be integrated, such as growth monitoring and promotion, counselling on breastfeeding and complementary feeding, early childhood development programs and the early detection and treatment of severe wasting [31]. Current reliance on polio vaccination campaigns to deliver VAS has presented a risk of declined VAS coverage as polio programs cease [32], so identifying other routine community-based delivery programs to integrated VAS should be prioritized.

For peer review only

As the global vitamin A landscape evolves, it is important for national governments to consider how VAS programs can be positioned in combination with other parallel vitamin A interventions to reduce risks for deficiency for all children. VAS has short-term benefits for children (boosting serum retinol for approximately two months after administration) [33], so other interventions are necessary to sustainably maintain adequate vitamin A intake through the diet. For the countries included in this study, 51% (n=25) have nationally mandated the large-scale vitamin A fortification of industrially produced foods items [34]. However, poorer and rural populations - where VAS coverage is often lower – often consume small quantities of fortified food items (e.g., cooking oil, sugar, wheat flour) and may therefore derive limited benefit from industrial vitamin A fortification schemes [35]. Programs aimed at broader improvements in dietary diversity have also been recommended [36], but variation in diets between and within countries pose challenges when scaling up across different contexts. With multiple parallel vitamin A interventions, future studies that simultaneously consider the individual and combined contributions of all vitamin A interventions could help governments orient their national vitamin A strategies.

Evaluating equity dimensions of VAS programs by stratifying coverage estimates using multiple variables can help identify potential gaps in national programs' delivery strategies. This analysis drew from the DHS Household and Woman's Questionnaires to identify enabling determinants to undernutrition that could affect VAS coverage. If differences in VAS coverage are detected between populations, some of these geographic determinants (e.g., place of residence, administrative regions of the country) can serve as indicators that lead to practical

For peer review only

recommendations for programs to adopt to bridge coverage gaps (e.g., strengthening programs in a specific region of the country or for a specific demographic). In contrast, other determinants, while useful to evaluate whether VAS programs are broadly equitable, lack clear operational directions on how programs can specifically target populations that are left behind (e.g., socioeconomic position, caregiver's social independence, caregiver's decision-making autonomy). This study explored several different indicators made available in the DHS, and VAS programs could benefit from further research aimed at interpreting the combination of multiple indicators in the context of a country's current VAS delivery strategy (e.g., campaign versus routine delivery).

In our analysis, there are several limitations that are important to consider. First, the question specific to VAS in the DHS questionnaire is dependent on recall by the caregiver of the child. The percentage of respondents who could prove VAS reception using home-based vaccination records varied between countries, but for most countries (69%; n=34) less than half of respondents had proof of VAS reception (Supplementary Figure 10). For children whose records depend solely on recall by the caregiver, recall error (e.g., confusing VAS droplets with polio droplets, imprecision in the exact date of reception, confusion between multiple children) is more likely. Second, in settings where VAS is delivered via biannual campaigns, interpretation of DHS VAS coverage must cautiously consider the timing of the survey relative to the timing of the campaign (e.g., VAS coverage may be underestimated if the DHS was implemented immediately before the start of a new campaign). Across several settings, the characteristics of children missed by current VAS programs remained relatively consistent, but recommendations

For peer review only - http://bmjopen.bmj.com/site/about/guidelines.xhtml

related to specific settings would benefit from country-specific interpretation to place this information in the context of a country's current VAS policies and programs. Third, the dietary data collected as part of the DHS only contains one dichotomous recall of whether vitamin A-rich foods were consumed in the past 24 hours by complementary feeding children. No information is available regarding food consumption quantities, weekly variation in diets, or for children aged 24 – 59 months. With this kind of dietary data, it is not possible to understand whether VAS is being administered to children who have inadequate dietary vitamin A intake or who have intakes that exceed daily upper limits to put children at risk for toxicity. To fully understand whether VAS programs are addressing children with the greatest vitamin A needs, VAS coverage should be estimated alongside other sub-national nutritional assessment data (e.g., vitamin A inadequacy from dietary assessment) to identify populations with both inadequate dietary vitamin A intake and low VAS coverage. Considering these limitations, VAS coverage data available as part of the DHS is not recommended to replace either single semester coverage, two-dose coverage, or efforts to advocate for additional repeated, representative surveys. However, DHS data analyzed in parallel to these other available data can provide a more complete understanding of the VAS context in a country to inform governments on national program performance and help characterize missed children so that strategies can be adapted to reach them.

For peer review only

For peer review only - http://bmjopen.bmj.com/site/about/guidelines.xhtml

CONCLUSION

This analysis further contributes clear and consistent evidence that VAS programs are unable to reach all eligible infants and children. This analysis also highlighted inequity in access to VAS, as children who are likely the most in need are more often not reached by this life-saving intervention. Three decades after the WHO first recommended high-dose vitamin A supplements to infants and children aged 6 – 59 months, countries with high risks for vitamin A deficiency have not yet achieved the goal of universal VAS coverage. Particular attention should focus on settings with low coverage, where children who are the most in need are also more likely to be missed. While DHS data can be useful to identify variations in VAS coverage based on equity dimensions already included in the questionnaire, challenges remain for programmatic questions requiring current, context-specific data.

For peer review only

Contributions: K.T., K.P.A. and A.H. designed the study. K.T., A.I., K.P.A., and A.H. defined the variables for disaggregation. K.T. and H.E. wrote the code for data extraction and analysis. F.S., G.M., E.J.M.J., and A.H. contributed the contextualization of analysis results to the broader field of research and policy. All authors critically reviewed and approved the final manuscript.

Funding: No funding was provided for this study. K.T., K.P.A., F.S., and E.J.M.J. receive funding from the Bill and Melinda Gates Foundation through the Micronutrient Action Policy Support (MAPS) project (INV-002855). K.T., A.I., G.M., and A.H. are staff members at UNICEF. The contents of this article do not necessarily reflect the views of the Bill and Melinda Gates Foundation or UNICEF.

Competing Interests: None declared

Data availability statement: Data are available in a public, open access repository. The data used are DHS and are publicly available at https://dhsprogram.com/data/available-datasets.cfm.

Ethics statement: This work is a secondary analysis of publicly available, de-identified data collected as part of the DHS. No Ethics Committee or Institutional Board approval is required.

For peer review only

REFERENCES

1. United Nations Children's Fund. UNICEF Nutrition Strategy 2020–2030. New York, USA: UNICEF, 2020.
2. Independent Expert Group of the Global Nutrition Report. 2020 Global Nutrition Report: Act on equity to end malnutrition. Bristol, United Kingdom: Development Initiatives Poverty Research Ltd., 2020. doi:10.2499/9780896295841
3. Stevens GA, Bennett JE, Hennocq Q, *et al.* Trends and mortality effects of vitamin A deficiency in children in 138 low-income and middle-income countries between 1991 and 2013: A pooled analysis of population-based surveys. *Lancet Glob Health* Published Online First: 2015. doi:10.1016/S2214-109X(15)00039-X
4. Imdad A, Mayo-Wilson E, Herzer K, *et al.* Vitamin A supplementation for preventing morbidity and mortality in children from six months to five years of age. *Cochrane Database of Systematic Reviews* Published Online First: 2017. doi:10.1002/14651858.CD008524.pub3
5. Horton S, Blum LS, Diouf M, *et al.* Delivering vitamin A supplements to children aged 6-59 months: Comparing delivery through campaigns and through routine health services in Senegal. *Curr Dev Nutr* 2018;**2**:nzy006. doi:10.1093/cdn/nzy006
6. Imdad A, Herzer K, Mayo-Wilson E, *et al.* Vitamin A supplementation for preventing morbidity and mortality in children from 6 months to 5 years of age. In: *Cochrane Database of Systematic Reviews*. 2010. doi:10.1002/14651858.cd008524.pub2
7. World Health Organization. Guideline: Vitamin A supplementation in infants and children 6 – 59 months of age. Geneva, Switzerland, WHO, 2011.

For peer review only

For peer review only - http://bmjopen.bmj.com/site/about/guidelines.xhtml

8 United Nations Children's Fund. Coverage at a Crossroads: New directions for vitamin A supplementation programmes. New York, USA: UNICEF, 2018.

9 Fawzi WW, Wang D. When should universal distribution of periodic high-dose vitamin A to children cease? *American Journal of Clinical Nutrition* 2021;**113**:769–71. doi:10.1093/ajcn/nqaa428

10 Sbarra AN, Rolfe S, Nguyen JQ, *et al.* Mapping routine measles vaccination in low- and middle-income countries. *Nature* Published Online First: 2021. doi:10.1038/s41586-020-03043-4

11 Hosseinpoor AR, Bergen N, Schlotheuber A, *et al.* State of inequality in diphtheria-tetanus-pertussis immunisation coverage in low-income and middle-income countries: a multicountry study of household health surveys. *Lancet Glob Health* Published Online First: 2016. doi:10.1016/S2214-109X(16)30141-3

12 Global Alliance for Vitamin A (GAVA). Monitoring of Vitamin A Supplementation: A Guide for National Programme Managers. Ottawa, Canada: Micronutrient Initiative, 2017.

13 The Global Alliance for Vitamin A (GAVA). Monitoring of Vitamin A Supplementation: A Guide for District (Area-based) Programme Managers. Ottowa, Canada: Micronutrient Initiative, 2017.

14 Short Fabic M, Choi Y, Bird S. A systematic review of Demographic and Health Surveys: data availability and utilization for research. *Bull World Health Organ* Published Online First: 2012. doi:10.2471/blt.11.095513

15 [datasets] ICF (Distributor), 2010-2019. Demographic and Health Surveys (various). Funded by USAID. Rockville, Maryland: ICF (Distributor).

For peer review only - http://bmjopen.bmj.com/site/about/guidelines.xhtml

16 Corsi DJ, Neuman M, Finlay JE, *et al.* Demographic and health surveys: A profile. *Int J Epidemiol* Published Online First: 2012. doi:10.1093/ije/dys184

17 ICF (Distributor). Demographic and Health Surveys Standard Recode Manual for DHS7. Rockville, Maryland: 2018.

18 ICF (Distributor). Demographic and Health Survey Interviewer's Manual. Rockville, Maryland: 2020.

19 World Health Organization. Complementary feeding of young children in developing countries: a review of current scientific knowledge. Geneva, Switzerland: WHO, 1998. https://apps.who.int/iris/handle/10665/65932

20 Ewerling F, Lynch JW, Victora CG, *et al.* The SWPER index for women's empowerment in Africa: development and validation of an index based on survey data. *Lancet Glob Health* Published Online First: 2017. doi:10.1016/S2214-109X(17)30292-9

21 Pullum T, Ren R, Elkasabi M. Webinar: Weights & Adjustments for DHS Surveys. The DHS Program. 2015.https://www.youtube.com/watch?v=5UDPYLwWHIA (accessed 7 Oct 2021).

22 Database of Global Administrative Areas (GADM). GADM Data. 2018.https://gadm.org/data.html (accessed 1 Feb 2022).

23 Watson OJ, FitzJohn R, Eaton JW. rdhs: an R package to interact with The Demographic and Health Surveys (DHS) Program datasets. *Wellcome Open Res* Published Online First: 2019. doi:10.12688/wellcomeopenres.15311.1

24 Freedman Ellis G, Lumley T, Zoltak T, *et al.* srvyr: 'dplyr'-Like Syntax for Summary Statistics of Survey Data. 2021.https://cran.r-project.org/web/packages/srvyr/index.html

For peer review only

25 Wickham H, Averick M, Bryan J, *et al.* Welcome to the Tidyverse. *J Open Source Softw* 2019;**4**:1686. doi:10.21105/joss.01686

26 Aghaji AE, Duke R, Aghaji UCW. Inequitable coverage of vitamin A supplementation in Nigeria and implications for childhood blindness. *BMC Public Health* 2019;**19**:282. doi:10.1186/s12889-019-6413-1

27 Millogo O, Doamba JEO, Sié A, *et al.* Geographical variation in the association of child, maternal and household health interventions with under-five mortality in Burkina Faso. *PLoS One* 2019;**14**:e0218163. doi:10.1371/journal.pone.0218163

28 Ribaudo I, Aramburú-Duclos CB, Blitchtein D. Is Vitamin A Supplementation Associated With Anemia in Children Under 5 Years of Age in Peru: Secondary Analysis of the “Demographic Health Survey” 2015-2018? *Glob Pediatr Health* 2021;**8**:1–11. doi:10.1177/2333794X211048385

29 White JM, Bégin F, Kumapley R, *et al.* Complementary feeding practices: Current global and regional estimates. *Matern Child Nutr* Published Online First: 2017. doi:10.1111/mcn.12505

30 Hodges MH, Sesay FF, Kamara HI, *et al.* Integrating Vitamin A Supplementation at 6 months into the Expanded Program of Immunization in Sierra Leone. *Matern Child Health J* Published Online First: 2015. doi:10.1007/s10995-015-1706-1

31 Hasman A, Moloney G, Aguayo V. Regular vitamin A supplementation: Prioritizing the youngest children. *American Journal of Clinical Nutrition* 2021;**114.1**:390–1. doi:10.1093/ajcn/nqab179

For peer review only

For peer review only - http://bmjopen.bmj.com/site/about/guidelines.xhtml

32 Chehab ET, Anya BPM, Onyango AW, *et al.* Experience of integrating vitamin A supplementation into polio campaigns in the African Region. *Vaccine* Published Online First: 2016. doi:10.1016/j.vaccine.2016.05.056

33 Palmer AC, West KP, Dalmiya N, *et al.* The use and interpretation of serum retinol distributions in evaluating the public health impact of vitamin A programmes. Public Health Nutr. 2012. doi:10.1017/S1368980012000560

34 Food Fortification Initiative, Global Alliance for Improved Nutrition, Network IG, *et al.* Global Fortification Data Exchange. 2018.https://fortificationdata.org/ (accessed 7 Oct 2021).

35 Tang K, Adams KP, Ferguson EL, *et al.* Modeling food fortification contributions to micronutrient requirements in Malawi using Household Consumption and Expenditure Surveys. *Ann N Y Acad Sci* 2021;:1–18. doi:10.1111/nyas.14697

36 Karlsson O, Kim R, Hasman A, *et al.* Consumption of Vitamin-A-Rich Foods and Vitamin A Supplementation for Children under Two Years Old in 51 Low- and Middle-Income Countries. *Nutrients* 2022;**14**:188. doi:10.3390/nu14010188

For peer review only

FIGURE LEGEND

Figure 1. Vitamin A supplementation (VAS) coverage among children 6-23 months who have and have not recently consumed vitamin A-rich foods (in the 24 hours prior to the interview). Philippines DHS did not collect data on child consumption of vitamin A-rich foods.

Figure 2. Vitamin A supplementation (VAS) coverage among children who have and have not received (A) the first dose of the diphtheria-tetanus-pertussis (DTP) vaccine and (B) the first dose of the measles containing vaccine (indicator of access to healthcare systems and services).

Figure 3. Vitamin A supplementation (VAS) coverage disaggregated by geography: (A) rural vs urban residence and (B) administrative regions.

Figure 4. Vitamin A supplementation (VAS) coverage maps by administrative regions (maps of all countries available in the supplementary material)

Supplementary Figure 1. Vitamin A supplementation (VAS) coverage between administrative regions for included countries in East and Southern Africa.

Supplementary Figure 2. Vitamin A supplementation (VAS) coverage between administrative regions for included countries in West and Central Africa.

Supplementary Figure 3. Vitamin A supplementation (VAS) coverage between administrative regions for included countries in South Asia, East Asia, and the Western Pacific.

Supplementary Figure 4. Vitamin A supplementation (VAS) coverage between administrative regions for included countries in the Eastern Mediterranean Region.

Supplementary Figure 5. Vitamin A supplementation (VAS) coverage between administrative regions for included countries in the Americas.

Supplementary Figure 6. Vitamin A supplementation (VAS) coverage among children from households of the poorest versus the richest socioeconomic quintiles.

Supplementary Figure 7. Differences in vitamin A supplementation (VAS) coverage between children whose caregivers are the least versus the most socially independent in their country.

Supplementary Figure 8. Differences in vitamin A supplementation (VAS) coverage between children whose caregivers have the least versus the most decision-making autonomy in their country.

Supplementary Figure 9. Differences in vitamin A supplementation (VAS) coverage between children based on the educational attainment of the child's caregiver.

Supplementary Figure 10. Proportion of children who had received vitamin A supplementation (VAS) whose caregiver confirmed VAS reception with a vaccination card.

For peer review only

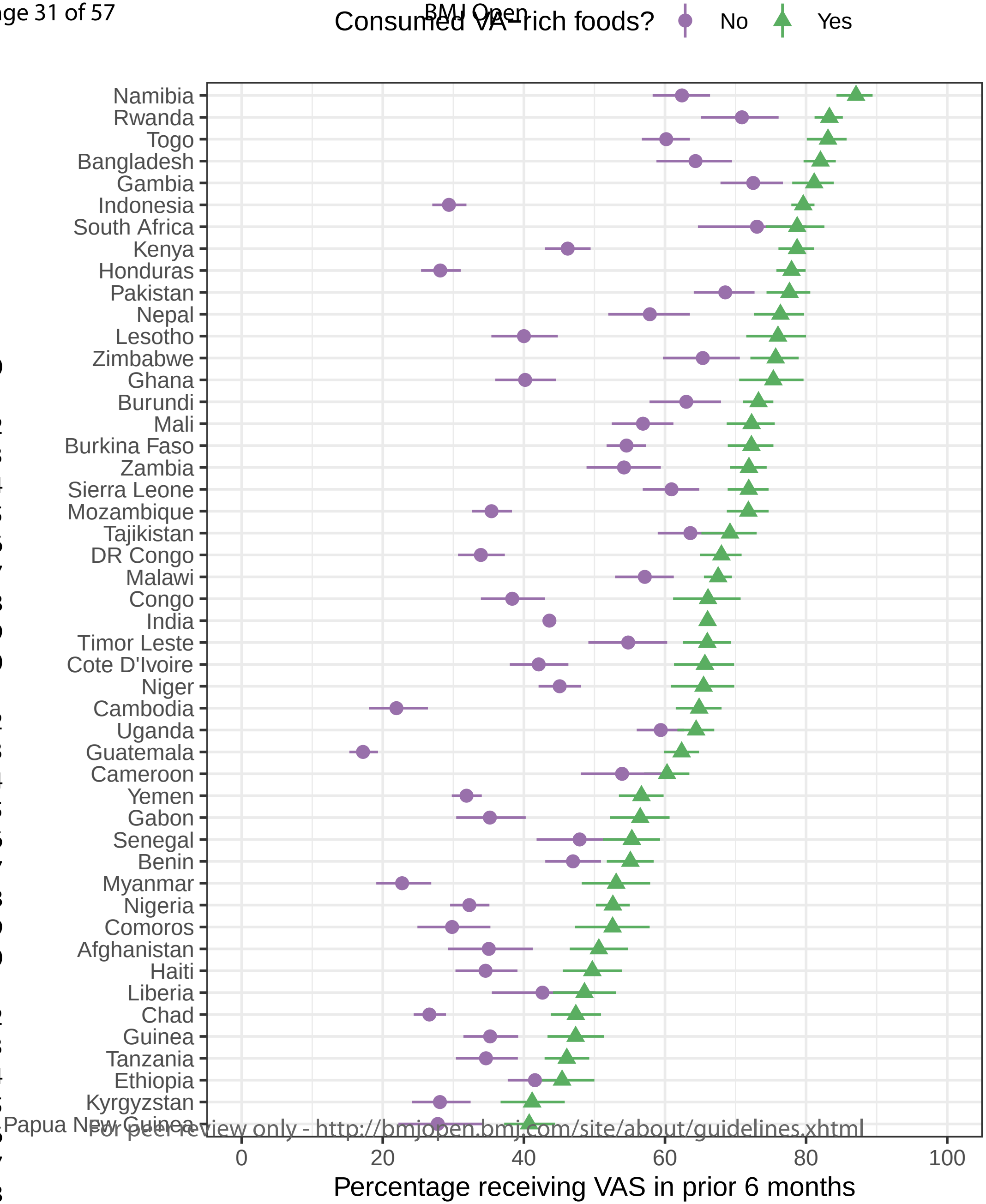
Consumed VA−rich foods?
No
Yes
Namibia
Rwanda
Togo
Bangladesh
Gambia
Indonesia
South Africa
Kenya
Honduras
Pakistan
Nepal
Lesotho
Zimbabwe
Ghana
Burundi
Mali
Burkina Faso
Zambia
Sierra Leone
Mozambique
Tajikistan
DR Congo
Malawi
Congo
India
Timor Leste
Cote D'Ivoire
Niger
Cambodia
Uganda
Guatemala
Cameroon
Yemen
Gabon
Senegal
Benin
Myanmar
Nigeria
Comoros
Afghanistan
Haiti
Liberia
Chad
Guinea
Tanzania
Ethiopia
Kyrgyzstan
Papua New Guinea
0
20
40
60
80
100
Percentage receiving VAS in prior 6 months

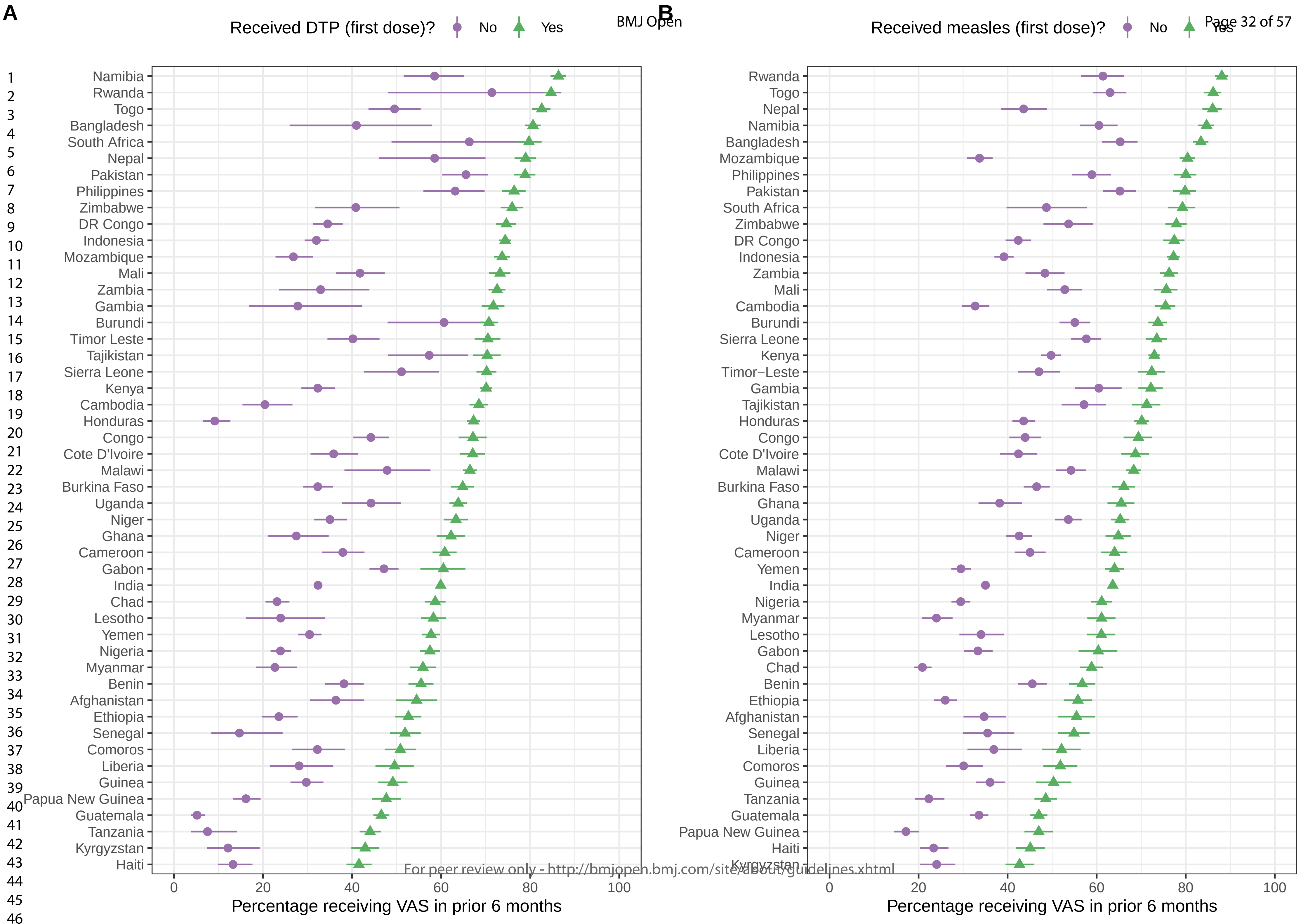
A
Received DTP (first dose)?
No
Yes
Namibia
Rwanda
Togo
Bangladesh
South Africa
Nepal
Pakistan
Philippines
Zimbabwe
DR Congo
Indonesia
Mozambique
Mali
Zambia
Gambia
Burundi
Timor Leste
Tajikistan
Sierra Leone
Kenya
Cambodia
Honduras
Congo
Cote D'Ivoire
Malawi
Burkina Faso
Uganda
Niger
Ghana
Cameroon
Gabon
India
Chad
Lesotho
Yemen
Nigeria
Myanmar
Benin
Afghanistan
Ethiopia
Senegal
Comoros
Liberia
Guinea
Papua New Guinea
Guatemala
Tanzania
Kyrgyzstan
Haiti
0
20
40
60
80
100
Percentage receiving VAS in prior 6 months
B
Received measles (first dose)?
No
Yes
Rwanda
Togo
Nepal
Namibia
Bangladesh
Mozambique
Philippines
Pakistan
South Africa
Zimbabwe
DR Congo
Indonesia
Zambia
Mali
Cambodia
Burundi
Sierra Leone
Kenya
Timor−Leste
Gambia
Tajikistan
Honduras
Congo
Cote D'Ivoire
Malawi
Burkina Faso
Ghana
Uganda
Niger
Cameroon
Yemen
India
Nigeria
Myanmar
Lesotho
Gabon
Chad
Benin
Ethiopia
Afghanistan
Senegal
Liberia
Comoros
Guinea
Tanzania
Guatemala
Papua New Guinea
Haiti
Kyrgyzstan
0
20
40
60
80
100
Percentage receiving VAS in prior 6 months

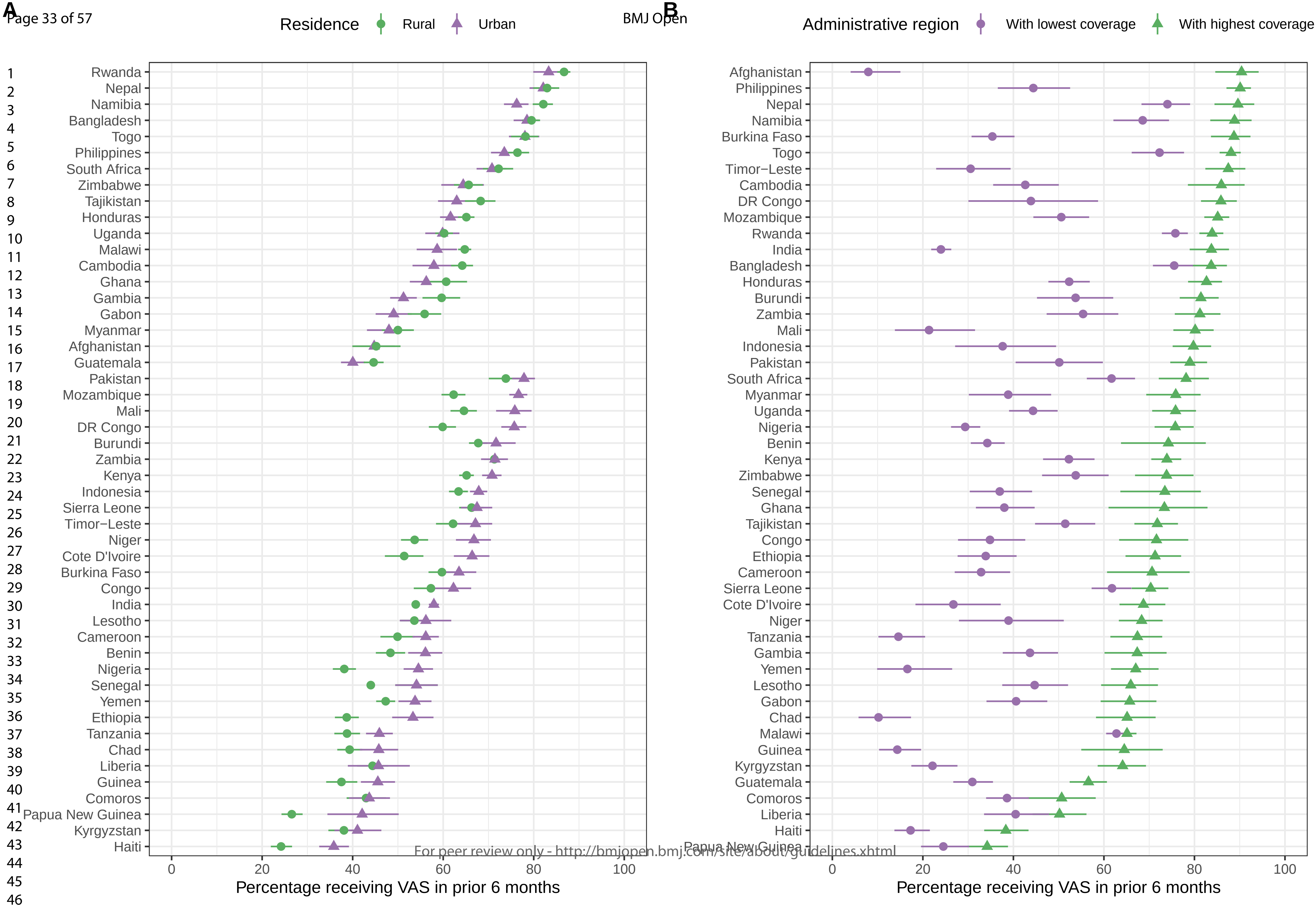

For peer review only - http://bmjopen.bmj.com/site/about/guidelines.xhtml

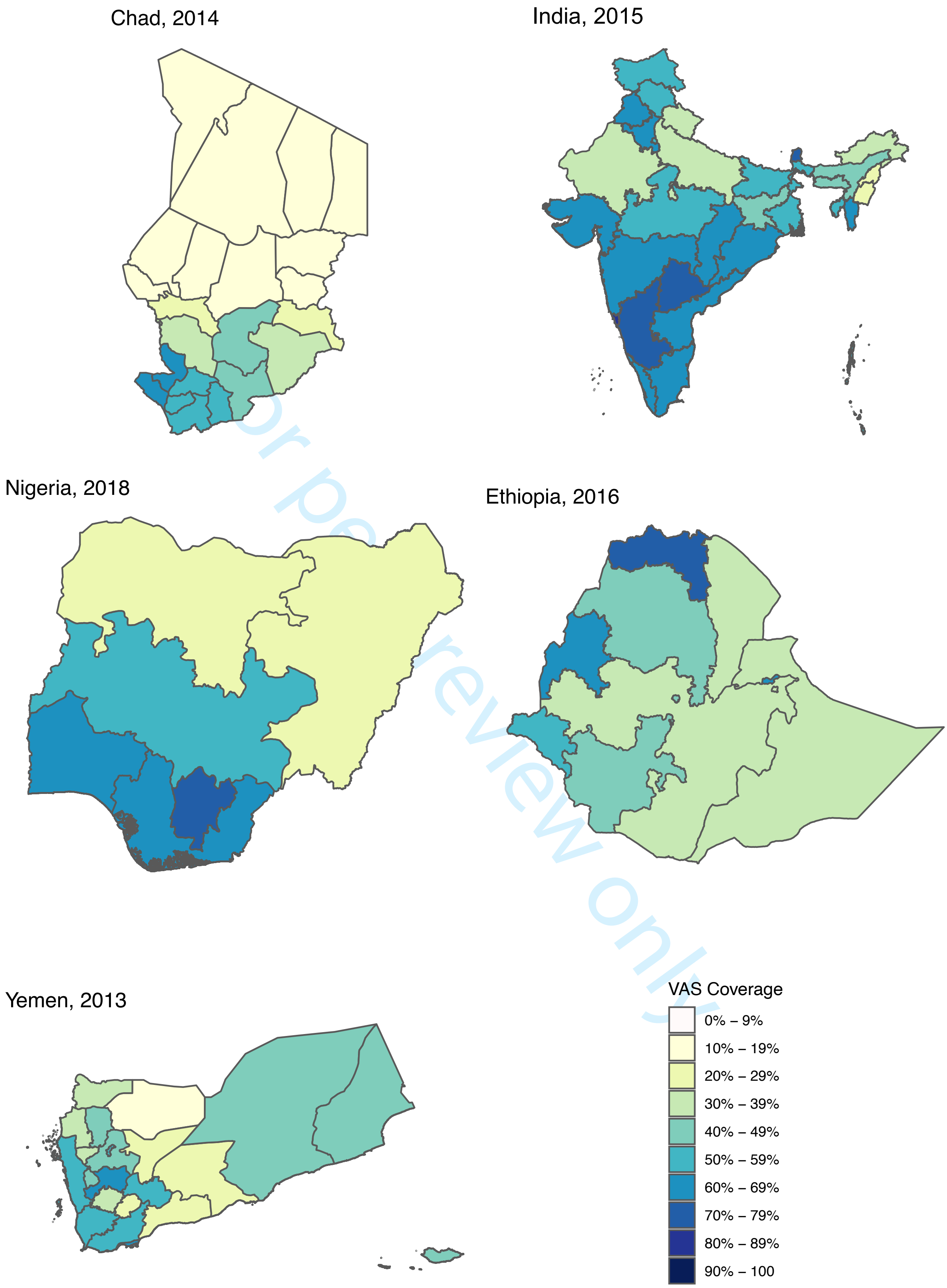
Chad, 2014
India, 2015
Nigeria, 2018
Ethiopia, 2016
Yemen, 2013
VAS Coverage
0% – 9%
10% – 19%
20% – 29%
30% – 39%
40% – 49%
50% – 59%
60% – 69%
70% – 79%
80% – 89%
90% – 100

Evaluating equity dimensions of infant and child vitamin A supplementation programs using Demographic and Health Surveys from 49 countries

- SUPPLEMENTARY MATERIAL –

Table of Contents

Supplementary Table 1. Countries included in the study and summary information on their respective Demographic and Health Surveys 2

Supplementary Table 2. Descriptive summary of the survey population of children aged 6-59 months for each country included in this study 4

Supplementary Figure 1. Vitamin A supplementation (VAS) coverage between administrative regions for included countries in East and Southern Africa. 6

Supplementary Figure 2. Vitamin A supplementation (VAS) coverage between administrative regions for included countries in West and Central Africa. 9

Supplementary Figure 3. Vitamin A supplementation (VAS) coverage between administrative regions for included countries in South Asia, East Asia, and the Western Pacific. 13

Supplementary Figure 4. Vitamin A supplementation (VAS) coverage between administrative regions for included countries in the Eastern Mediterranean Region. 16

Supplementary Figure 5. Vitamin A supplementation (VAS) coverage between administrative regions for included countries in the Americas. 17

Supplementary Figure 6. Vitamin A supplementation (VAS) coverage among children from households of the poorest versus the richest socioeconomic quintiles. 18

Supplementary Figure 7. Differences in vitamin A supplementation (VAS) coverage between children whose caregivers are the least versus the most socially independent in their country. .19

Supplementary Figure 8. Differences in vitamin A supplementation (VAS) coverage between children whose caregivers have the least versus the most decision-making autonomy in their country. 20

Supplementary Figure 9. Differences in vitamin A supplementation (VAS) coverage between children based on the educational attainment of the child's caregiver. 21

Supplementary Figure 10. Proportion of children who had received vitamin A supplementation (VAS) whose caregiver confirmed VAS reception with a vaccination card. 22

For peer review only - http://bmjopen.bmj.com/site/about/guidelines.xhtml

Supplementary Table 1. Countries included in the study and summary information on their respective Demographic and Health Surveys

Country	Survey year	Women surveyed (n)	Children recalled (n)
Afghanistan	2015	29,461	29,312
Bangladesh	2017	20,127	4137
Benin	2017	15,928	6378
Burkina Faso	2010	17,087	13,644
Burundi	2016	17,269	6161
Cambodia	2014	17,578	6909
Cameroon	2018	13,527	4337
Chad	2014	17,719	16,441
Comoros	2012	5329	2879
Congo	2011	10,819	8725
Cote D'Ivoire	2012	10,060	6882
Dem. Rep. of the Congo	2013	18,827	16,952
Ethiopia	2016	15,683	9504
Gabon	2012	8422	5747
Gambia	2019	11,865	3769
Ghana	2014	9396	5531
Guatemala	2015	25,914	12,071
Guinea	2018	10,874	3288
Haiti	2016	14,371	2922
Honduras	2011	22,757	10,592
India	2015	699,686	244,869
Indonesia	2017	45,607	17,367
Kenya	2014	31,079	19,887
Kyrgyzstan	2012	8208	4247
Lesotho	2014	6621	2855
Liberia	2019	8065	2493
Malawi	2015	24,562	7870
Mali	2018	10,519	4442
Mozambique	2011	13,745	10,214
Myanmar	2016	12,885	4550
Namibia	2013	9176	4818
Nepal	2016	12,862	2354
Niger	2012	11,160	11,338
Nigeria	2018	41,821	14,434
Pakistan	2017	15,068	5589
Papua New Guinea	2017	15,198	4436
Philippines	2017	25,074	4882
Rwanda	2019	14,634	7314
Senegal	2019	8649	3147
Sierra Leone	2019	15,574	4344
South Africa	2016	8514	3413
Tajikistan	2017	10,718	3016
Tanzania	2015	13,266	4864
Timor-Leste	2016	12,607	3332
Togo	2013	9480	6467
Uganda	2016	18,506	7138
Yemen	2013	25,434	15,136

For peer review only

Zambia	2018	13,683	4616
Zimbabwe	2015	9955	2775

For peer review only

For peer review only - http://bmjopen.bmj.com/site/about/guidelines.xhtml

Supplementary Table 2. Descriptive summary of the survey population of children aged 6-59 months for each country included in this study

Country	Received VAS in past 6 months, % (95%CI)	Did not recently consume vitamin A rich foods	Did not received DTP1	Did not received MCV1	Rural
Afghanistan	45.1 (40.9, 49.4)	62.5 (59.7, 65.2)	35.8 (31.9, 39.9)	49.3 (46.1, 52.6)	76.8 (71.4, 81.4)
Bangladesh	79.2 (77.6, 80.8)	41.8 (39.9, 43.7)	1.5 (1.0, 2.3)	19.7 (18.3, 21.3)	71.4 (68.4, 76.1)
Benin	51.4 (48.9, 53.9)	53.8 (51.9, 55.8)	16.2 (14.4, 18.3)	42.7 (40.5, 65.9)	61.3 (56.6, 65.9)
Burkina Faso	60.4 (57.8, 65.9)	73.6 (71.8, 75.4)	12.6 (11.3, 13.9)	28.7 (27.3, 30.1)	82.7 (79.3, 85.6)
Burundi	68.1 (66.2, 69.9)	33.1 (31.6, 34.7)	1.3 (1.0, 1.7)	17.3 (16.2, 18.4)	91.1 (88.2, 93.3)
Cambodia	63.3 (61.1, 65.4)	35.2 (32.7, 37.9)	9.7 (8.6, 10.9)	28.5 (26.9, 30.1)	85.3 (82.0, 88.1)
Cameroon	52.7 (50.3, 55.2)	41.4 (39.3, 43.4)	17.0 (14.6, 19.6)	39.5 (36.9, 42.2)	55.3 (49.1, 61.4)
Chad	40.6 (38.3, 43.0)	65.2 (62.9, 67.4)	47.5 (44.8, 50.3)	47.4 (45.3, 49.5)	80.4 (76.5, 83.9)
Comoros	43.2 (39.8, 46.6)	43.7 (39.8, 47.6)	24.4 (21.5, 27.6)	36.9 (33.9, 40.0)	72.7 (65.6, 78.8)
Congo	60.3 (57.4, 63.1)	40.5 (37.7, 43.3)	24.9 (22.8, 27.2)	35.5 (33.3, 37.8)	39.5 (33.4, 46.0)
Cote D'Ivoire	57.0 (53.8, 60.3)	54.1 (51.6, 56.7)	26.5 (23.6, 29.6)	43.7 (40.9, 46.5)	62.3 (55.6, 68.5)
Dem. Rep. of the Congo	64.8 (62.3, 67.1)	37.1 (35.0, 39.2)	22.5 (20.3, 24.9)	36.0 (34.2, 37.9)	69.1 (63.4, 74.3)
Ethiopia	40.1 (37.9, 42.8)	69.9 (67.0, 72.7)	27.8 (25.0, 30.9)	60.2 (57.2, 63.1)	88.8 (85.4, 91.5)
Gabon	50.1 (46.7, 53.6)	48.2 (45.1, 51.2)	47.4 (44.5, 50.3)	36.2 (33.6, 38.8)	15.6 (11.9, 20.1)
Gambia	54.1 (51.6, 56.5)	56.3 (53.5, 59.0)	2.4 (1.7, 3.2)	19.8 (17.9, 21.9)	34.1 (27.6, 41.2)
Ghana	58.7 (55.6, 61.7)	49.0 (45.7, 52.2)	7.2 (6.2, 8.5)	24.9 (23.3, 26.6)	54.9 (48.5, 61.1)
Guatemala	43.0 (41.3, 44.7)	19.2 (18.2, 20.3)	8.6 (7.9, 9.3)	30.0 (29.0, 31.0)	64.0 (59.9, 67.9)
Guinea	40.0 (37.3, 42.6)	66.7 (64.1, 69.1)	36.6 (33.2, 40.1)	60.1 (56.5, 63.7)	71.0 (65.9, 75.7)
Haiti	28.3 (26.3, 30.4)	55.9 (53.1, 58.6)	19.3 (16.8, 22.0)	42.7 (39.8, 45.6)	64.4 (58.6, 69.9)
Honduras	63.5 (62.0, 64.9)	32.2 (30.4, 34.0)	4.8 (4.4, 5.3)	24.5 (23.6, 25.5)	53.8 (50.2, 57.3)
India	55.1 (54.6, 55.6)	38.2 (37.6, 38.7)	15.0 (14.7, 15.3)	27.7 (25.3, 30.1)	68.1 (62.1, 73.6)
Indonesia	65.6 (64.2, 67.0)	34.3 (32.6, 36.0)	16.5 (15.3, 17.7)	30.2 (28.9, 31.5)	50.1 (46.7, 53.6)
Kenya	67.2 (65.9, 68.5)	41.3 (38.9, 43.8)	5.7 (5.2, 6.2)	24.8 (23.8, 25.7)	64.3 (60.9, 67.6)
Kyrgyzstan	39.0 (36.1, 41.9)	47.2 (43.9, 50.6)	4.0 (3.2, 5.1)	19.0 (17.6, 20.4)	70.1 (63.4, 76.1)
Lesotho	54.4 (51.6, 57.1)	52.6 (48.8, 56.4)	6.6 (5.5, 7.9)	24.1 (22.3, 26.0)	71.0 (64.2, 76.9)
Liberia	45.1 (41.1, 49.1)	54.7 (51.6, 57.8)	9.4 (7.5, 11.6)	35.9 (32.7, 39.2)	47.2 (39.4, 55.0)
Malawi	63.9 (62.5, 65.3)	38.9 (37.5, 40.4)	2.6 (2.1, 3.2)	18.4 (17.3, 19.5)	86.7 (83.3, 89.5)
Mali	66.9 (64.4, 69.3)	55.2 (52.9, 57.6)	17.4 (15.2, 19.9)	36.7 (34.0, 39.4)	80.0 (74.7, 84.3)

For peer review only - http://bmjopen.bmj.com/site/about/guidelines.xhtml

Mozambique	66.3 (64.2, 68.4)	35.7 (33.7, 37.6)	15.0 (13.2, 16.9)	30.1 (28.2, 32.1)	72.2 (67.8, 76.2)
Myanmar	49.6 (46.6, 52.5)	46.2 (42.9, 49.6)	18.0 (15.6, 20.7)	31.3 (28.8, 33.9)	77.4 (72.3, 81.9)
Namibia	79.2 (77.4, 81.0)	46.1 (43.2, 49.0)	8.7 (7.6, 9.9)	22.1 (20.6, 23.6)	51.0 (45.6, 56.4)
Nepal	82.5 (80.4, 84.4)	43.7 (40.3, 47.1)	3.3 (2.3, 4.8)	18.8 (16.8, 20.9)	46.1 (39.5, 52.8)
Niger	55.5 (52.8, 58.1)	72.7 (70.6, 74.8)	22.5 (20.5, 24.6)	41.1 (39.3, 42.9)	86.3 (83.2, 89.0)
Nigeria	44.7 (42.6, 46.8)	53.7 (52.1, 55.3)	36.1 (34.0, 38.3)	50.7 (48.8, 52.6)	61.4 (58.0, 64.8)
Pakistan	75.1 (72.5, 77.6)	59.0 (56.0, 62.0)	15.9 (12.9, 19.4)	33.6 (30.4, 36.9)	68.0 (62.1, 73.4)
Papua New Guinea	28.2 (25.9, 30.6)	29.7 (27.4, 32.2)	35.5 (32.3, 38.9)	44.1 (40.7, 47.5)	89.6 (86.5, 92.0)
Philippines	75.1 (73.1, 77.0)	-	14.3 (12.5, 16.4)	28.4 (26.0, 30.8)	55.7 (51.2, 60.1)
Rwanda	86.1 (84.7, 87.4)	14.9 (13.2, 16.7)	0.5 (0.3, 0.7)	23.6 (21.2, 26.2)	82.3 (78.1, 85.9)
Senegal	47.7 (44.6, 50.9)	59.5 (55.6, 63.3)	4.4 (3.2, 6.1)	23.6 (21.2, 26.2)	63.4 (54.9, 71.7)
Sierra Leone	66.7 (64.5, 68.8)	52.8 (50.7, 54.8)	5.5 (4.6, 6.5)	32.3 (30.3, 34.4)	65.0 (60.3, 69.5)
South Africa	71.3 (68.8, 73.7)	44.3 (40.7, 48.0)	7.7 (6.1, 9.8)	13.4 (11.3, 15.9)	36.3 (31.2, 41.7)
Tajikistan	67.2 (64.3, 69.9)	58.7 (56.0, 61.4)	7.4 (6.1, 9.0)	23.8 (21.5, 26.2)	79.0 (74.6, 82.9)
Tanzania	40.6 (38.4, 42.9)	40.3 (38.1, 42.5)	3.7 (2.7, 5.0)	24.0 (22.0, 26.3)	72.9 (68.1, 77.2)
Timor-Leste	63.6 (60.7, 66.4)	45.0 (42.4, 47.7)	22.9 (20.4, 25.6)	36.3 (33.8, 38.9)	71.6 (65.4, 77.1)
Togo	78.1 (75.8, 80.3)	45.6 (43.5, 47.8)	11.1 (9.7, 12.7)	34.7 (32.4, 37.0)	63.7 (57.7, 69.3)
Uganda	60.1 (58.4, 61.9)	48.0 (46.2, 49.8)	6.1 (5.4, 7.1)	28.3 (26.9, 29.9)	78.4 (74.5, 81.8)
Yemen	49.1 (47.2, 50.9)	54.8 (52.8, 56.7)	28.9 (26.9, 31.0)	43.3 (41.4, 45.2)	72.7 (68.3, 76.7)
Zambia	71.4 (69.5, 73.1)	39.6 (37.5, 41.7)	1.9 (1.4, 2.5)	18.5 (17.1, 20.0)	64.4 (58.8, 69.6)
Zimbabwe	65.2 (62.6, 67.8)	41.7 (39.3, 44.1)	11.8 (9.8, 14.2)	27.7 (25.3, 30.1)	68.1 (62.1, 73.6)

For peer review only - http://bmjopen.bmj.com/site/about/guidelines.xhtml

Supplementary Figure 1. Vitamin A supplementation (VAS) coverage between administrative regions for included countries in East and Southern Africa.

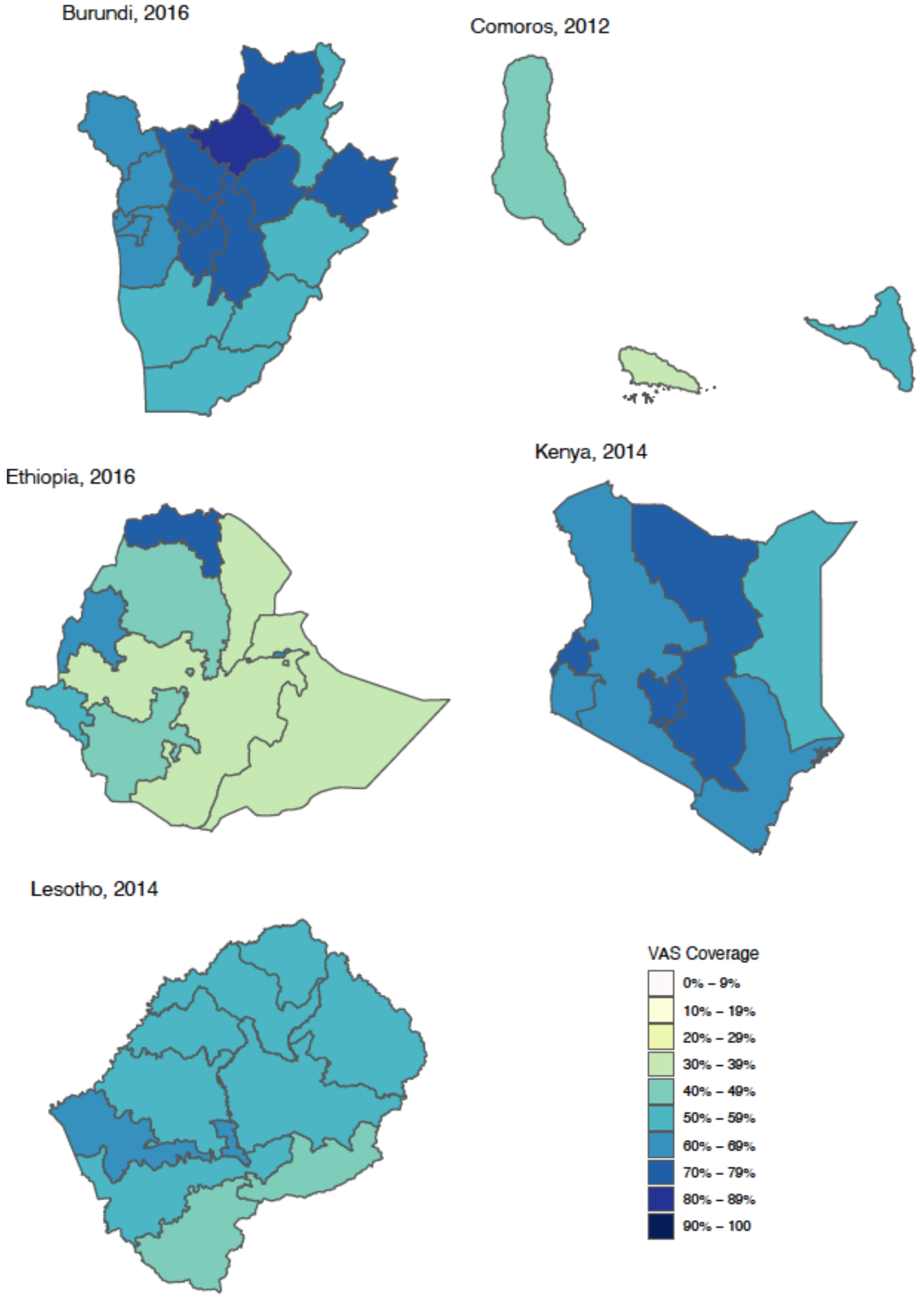

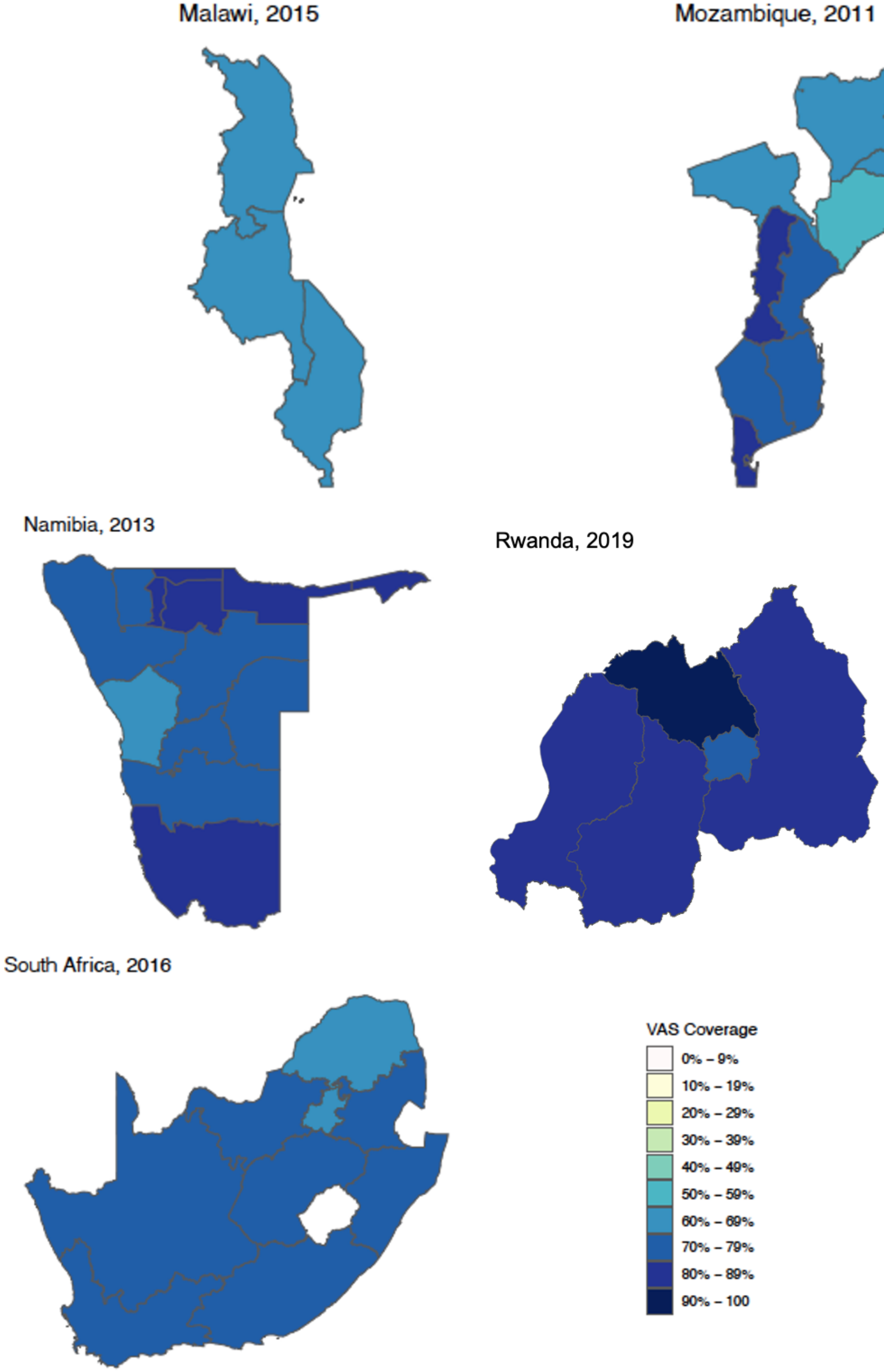
Malawi, 2015
Mozambique, 2011
Namibia, 2013
Rwanda, 2019
South Africa, 2016
VAS Coverage
0% – 9%
10% – 19%
20% – 29%
30% – 39%
40% – 49%
50% – 59%
60% – 69%
70% – 79%
80% – 89%
90% – 100

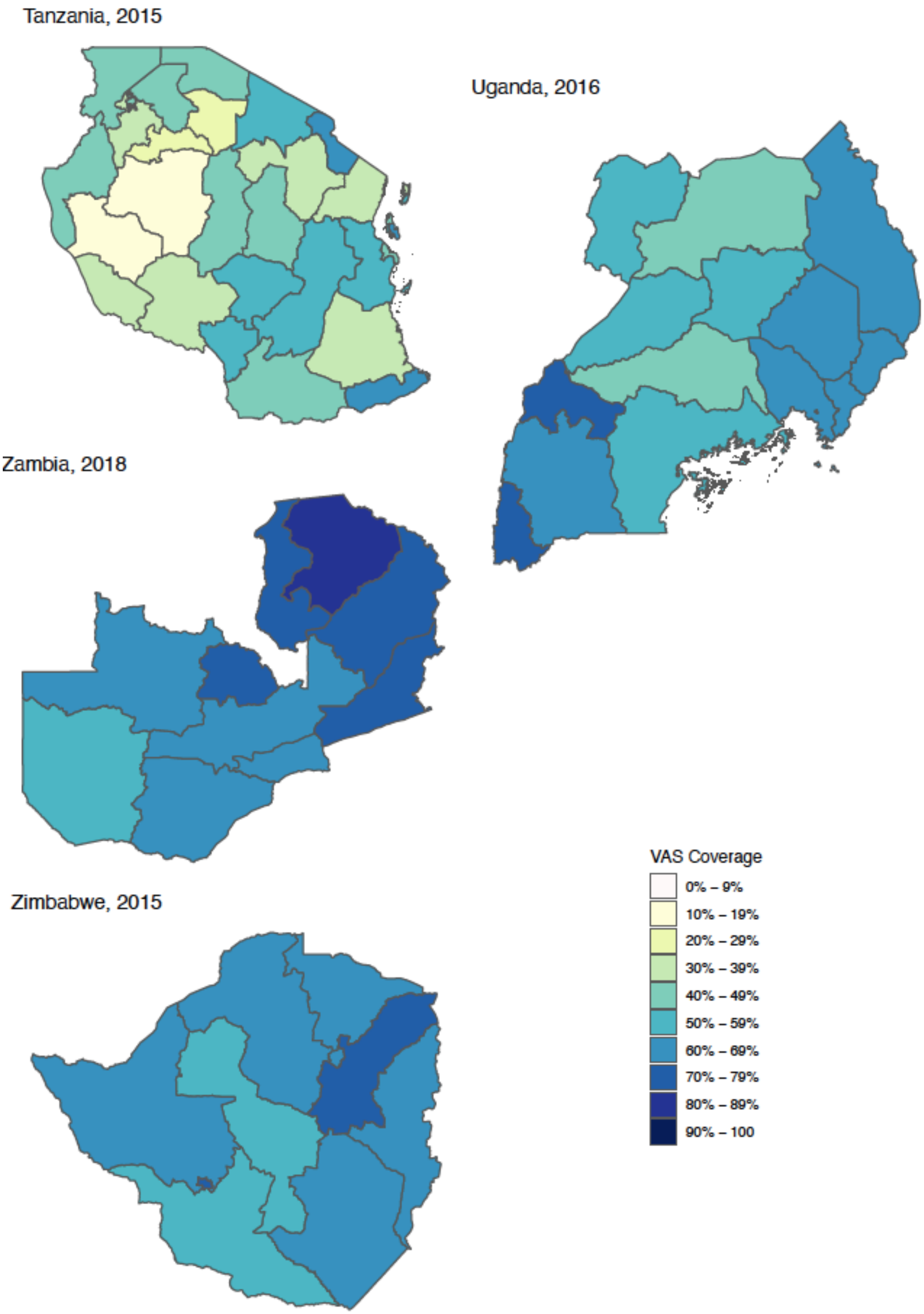
Tanzania, 2015
Uganda, 2016
Zambia, 2018
Zimbabwe, 2015
VAS Coverage
0% – 9%
10% – 19%
20% – 29%
30% – 39%
40% – 49%
50% – 59%
60% – 69%
70% – 79%
80% – 89%
90% – 100

Supplementary Figure 2. Vitamin A supplementation (VAS) coverage between administrative regions for included countries in West and Central Africa.

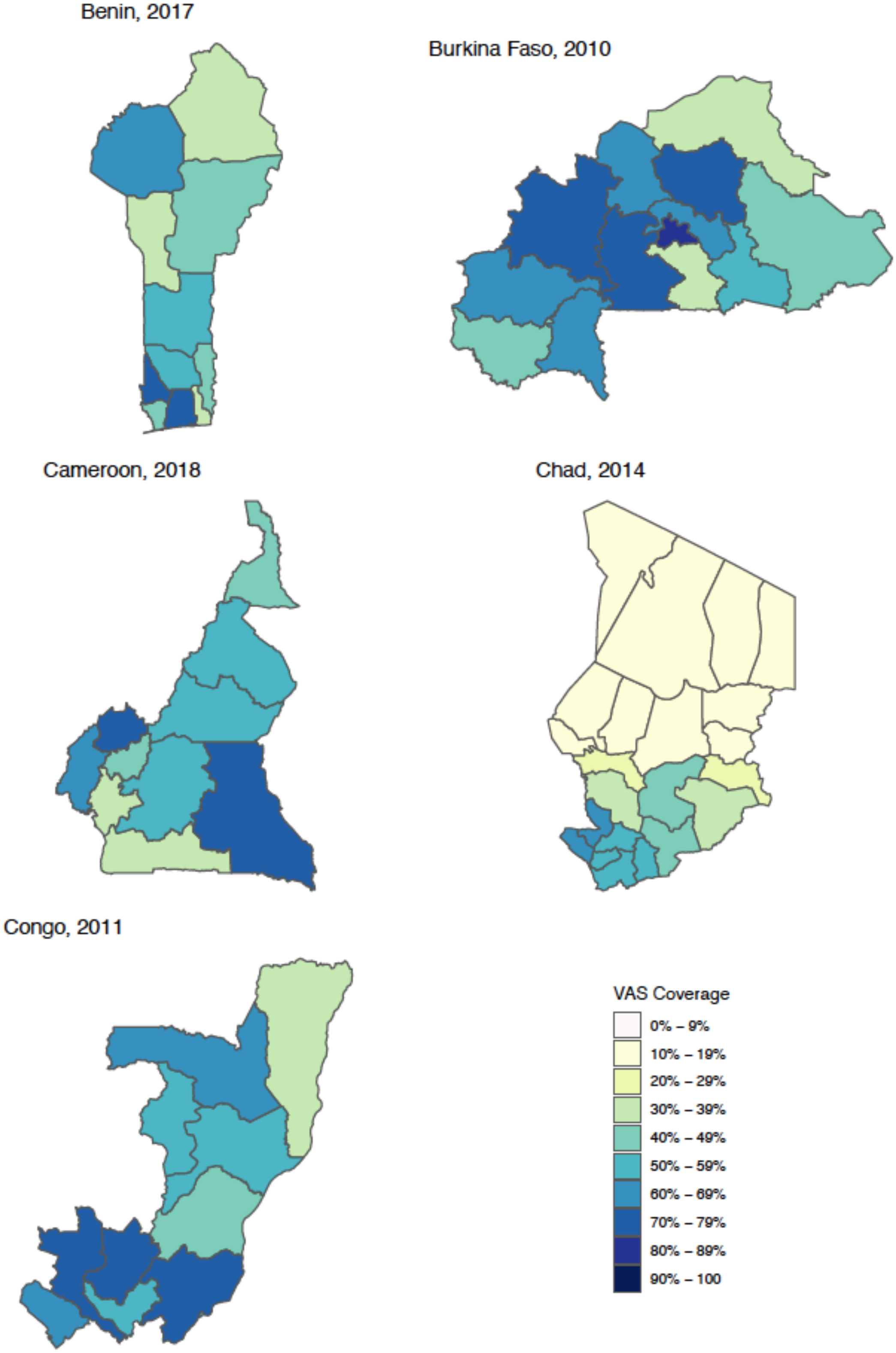

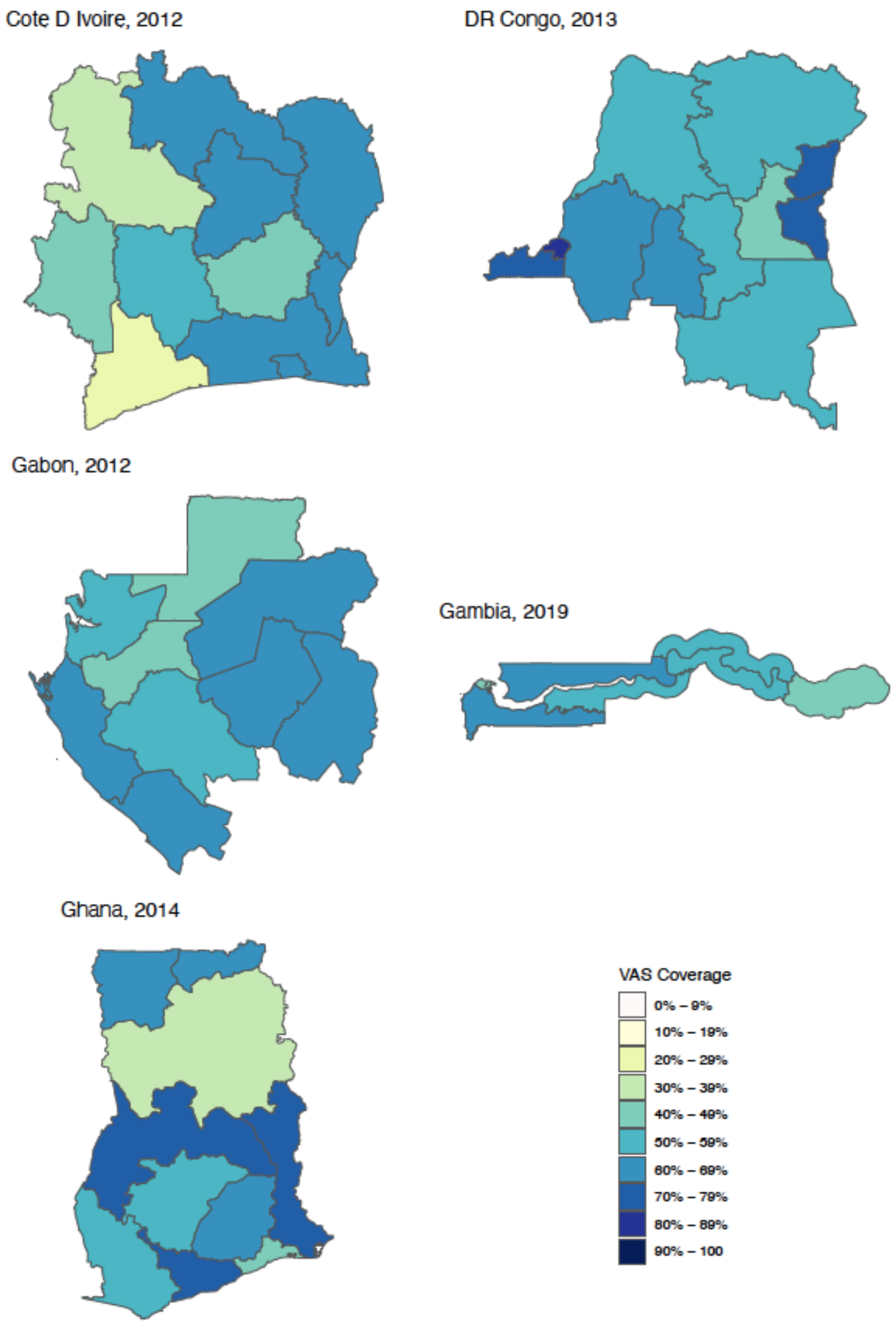
Cote D Ivoire, 2012
DR Congo, 2013
Gabon, 2012
Gambia, 2019
Ghana, 2014
VAS Coverage
0% – 9%
10% – 19%
20% – 29%
30% – 39%
40% – 49%
50% – 59%
60% – 69%
70% – 79%
80% – 89%
90% – 100

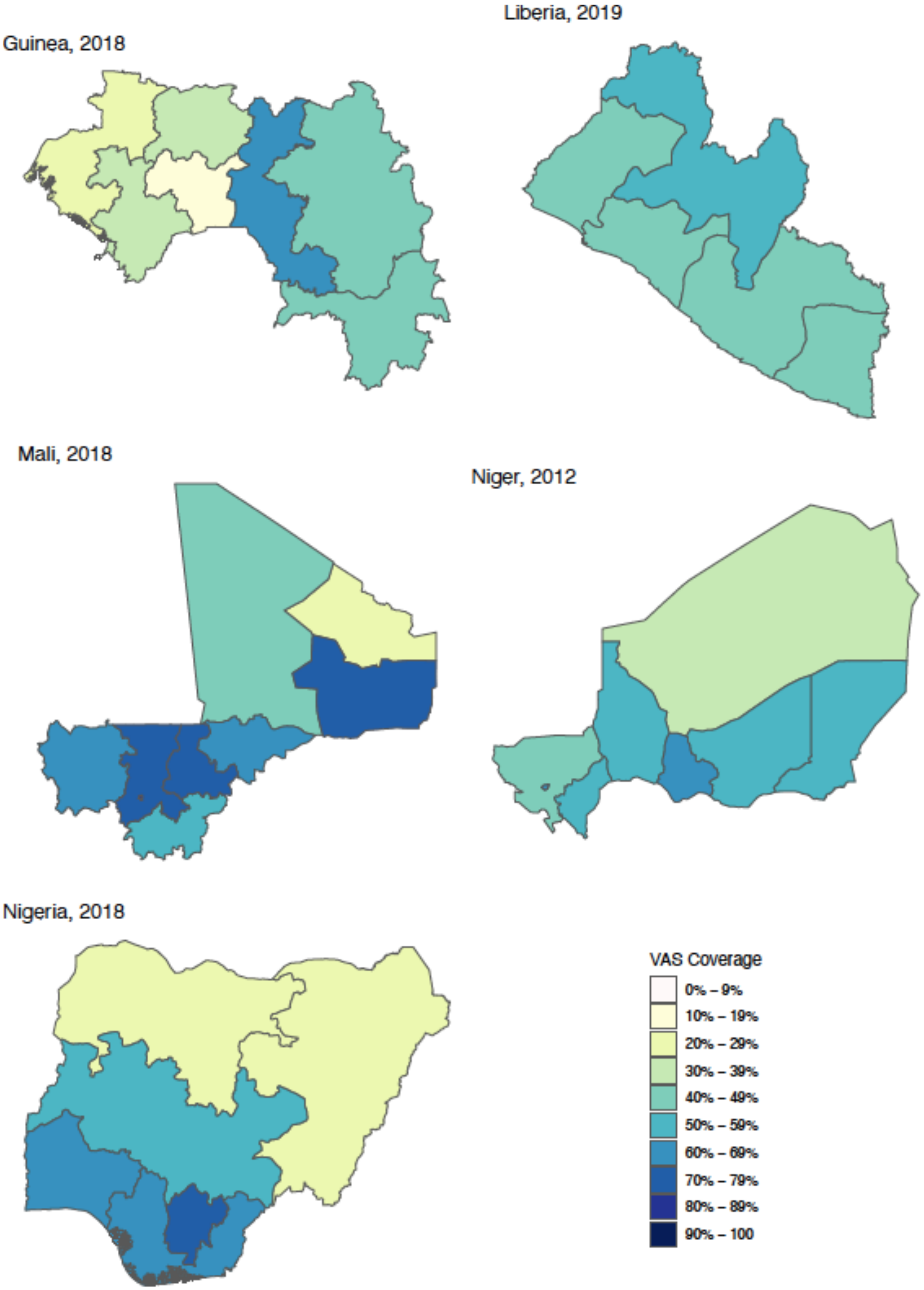
Guinea, 2018
Liberia, 2019
Mali, 2018
Niger, 2012
Nigeria, 2018
VAS Coverage
0% – 9%
10% – 19%
20% – 29%
30% – 39%
40% – 49%
50% – 59%
60% – 69%
70% – 79%
80% – 89%
90% – 100

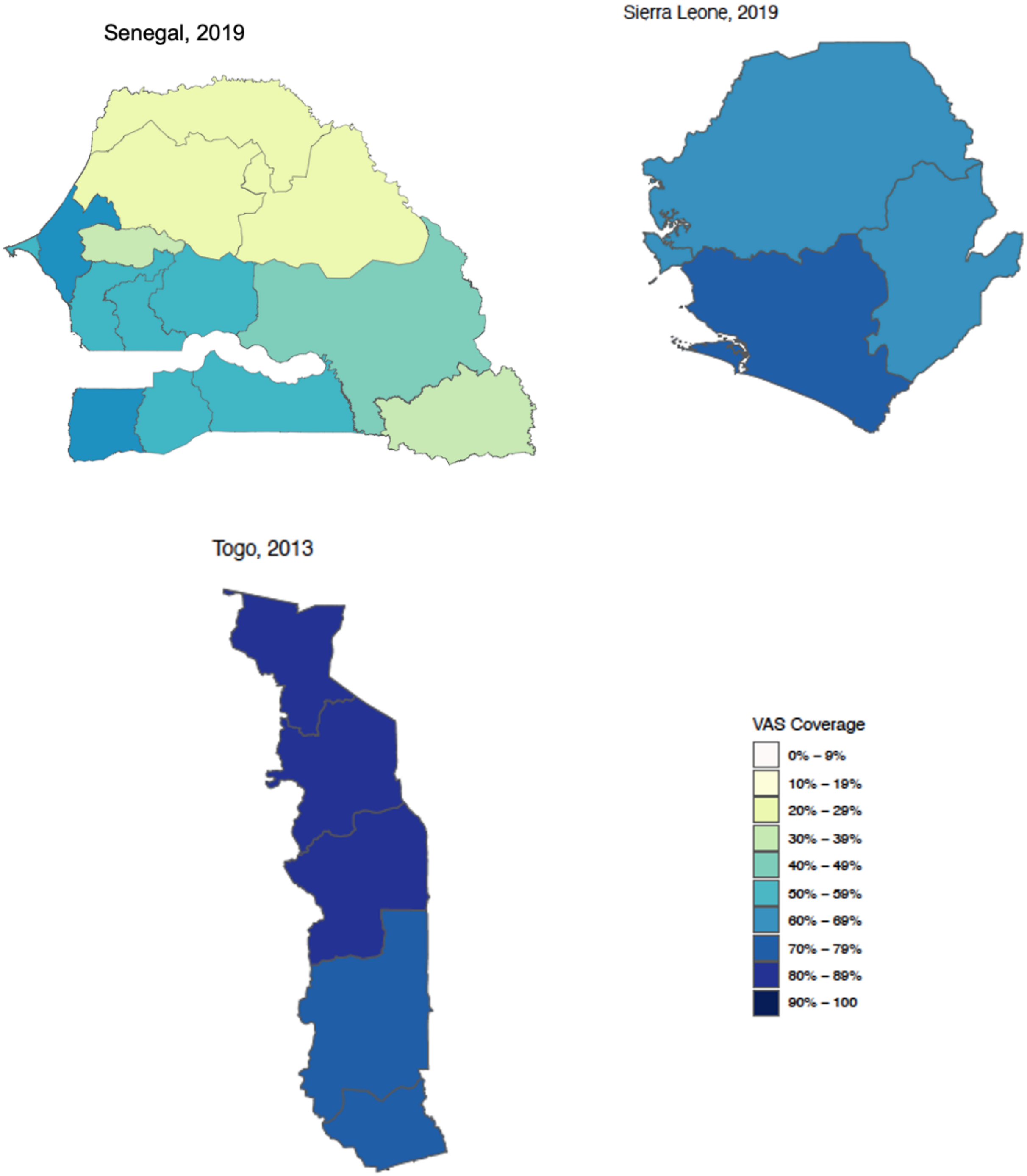
Senegal, 2019
Sierra Leone, 2019
Togo, 2013
VAS Coverage
0% – 9%
10% – 19%
20% – 29%
30% – 39%
40% – 49%
50% – 59%
60% – 69%
70% – 79%
80% – 89%
90% – 100

Supplementary Figure 3. Vitamin A supplementation (VAS) coverage between administrative regions for included countries in South Asia, East Asia, and the Western Pacific.

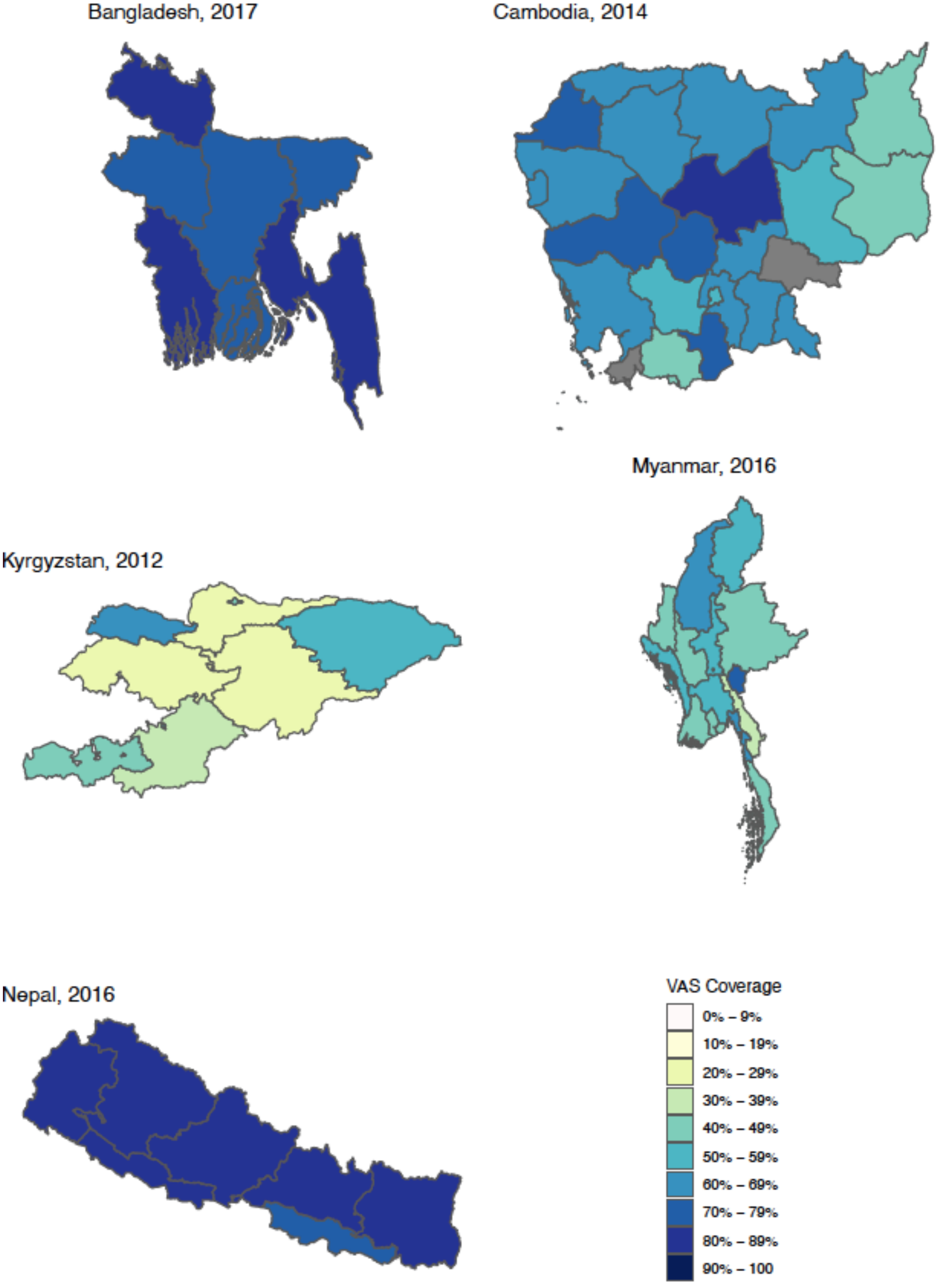

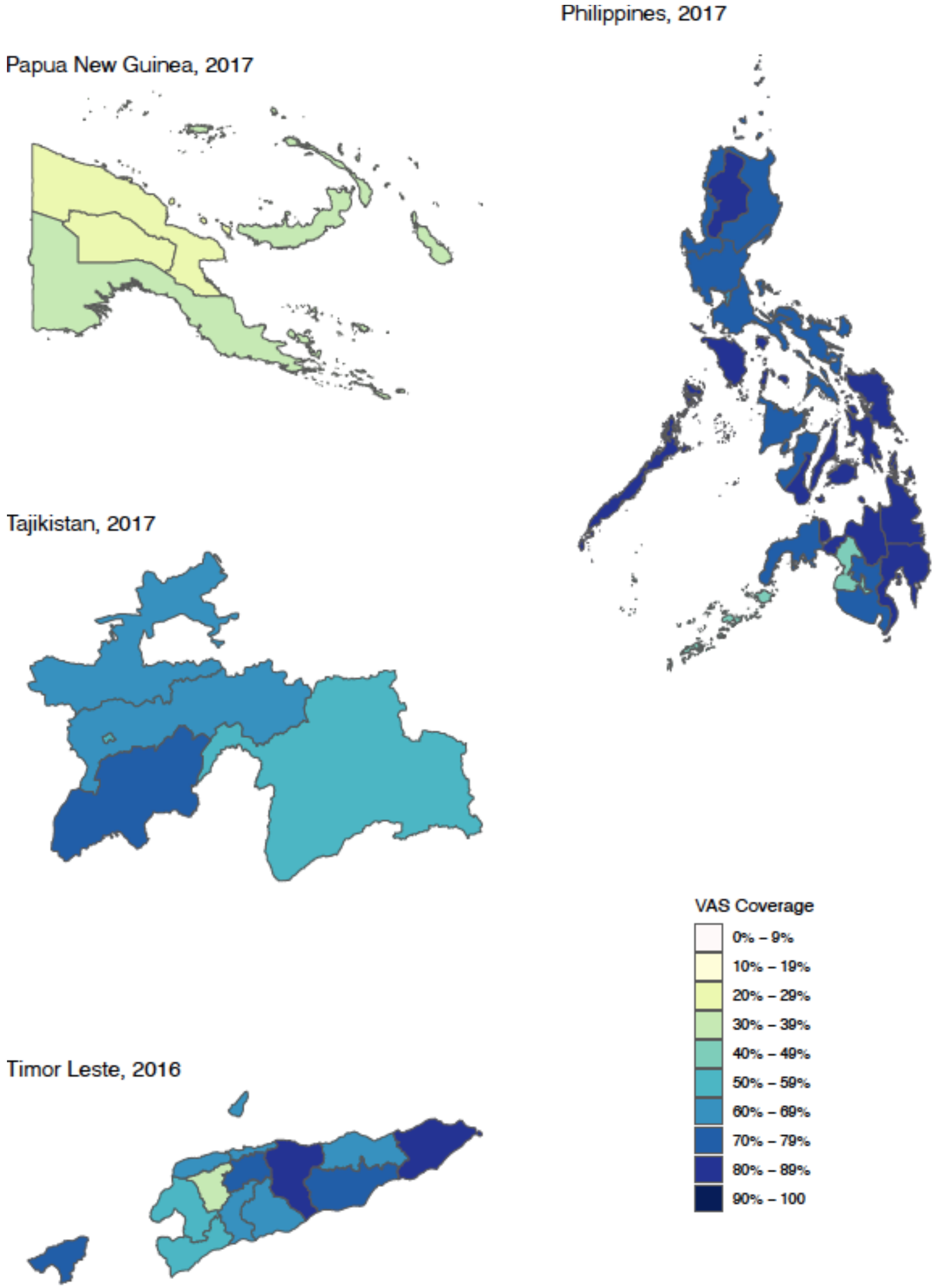
Philippines, 2017
Papua New Guinea, 2017
Tajikistan, 2017
Timor Leste, 2016
VAS Coverage
0% – 9%
10% – 19%
20% – 29%
30% – 39%
40% – 49%
50% – 59%
60% – 69%
70% – 79%
80% – 89%
90% – 100

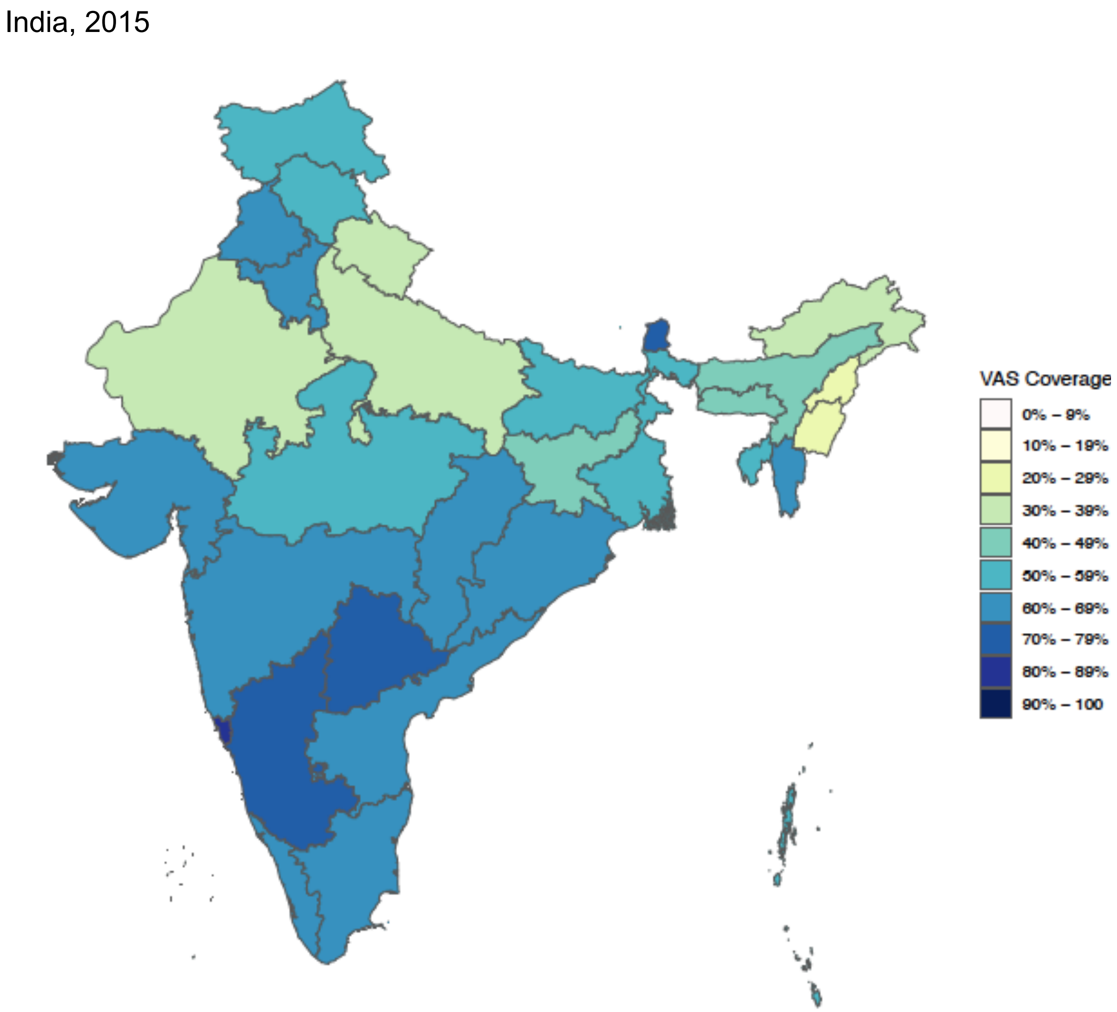

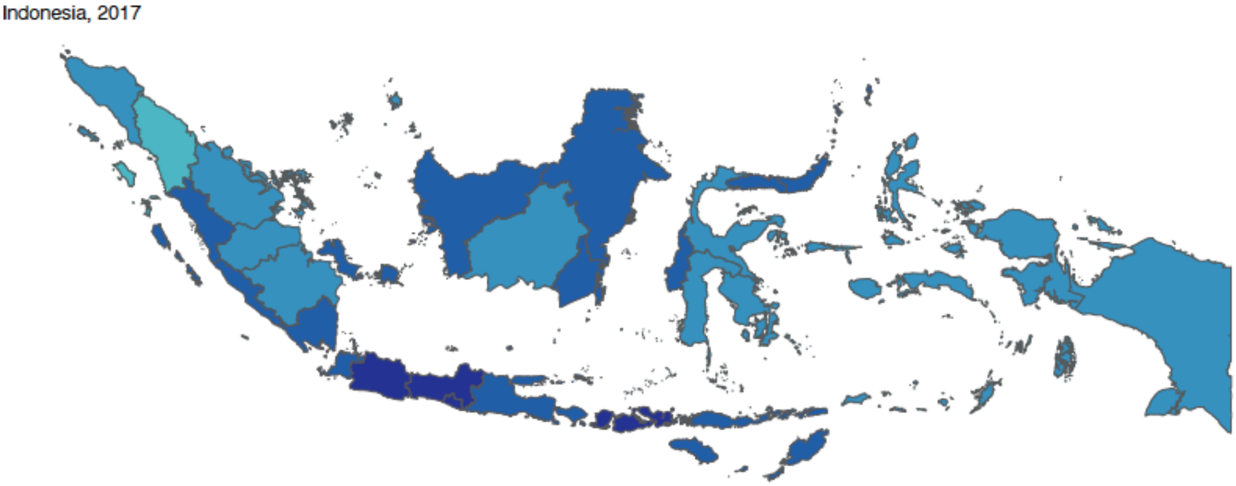

For peer review only - http://bmjopen.bmj.com/site/about/guidelines.xhtml

Supplementary Figure 4. Vitamin A supplementation (VAS) coverage between administrative regions for included countries in the Eastern Mediterranean Region.

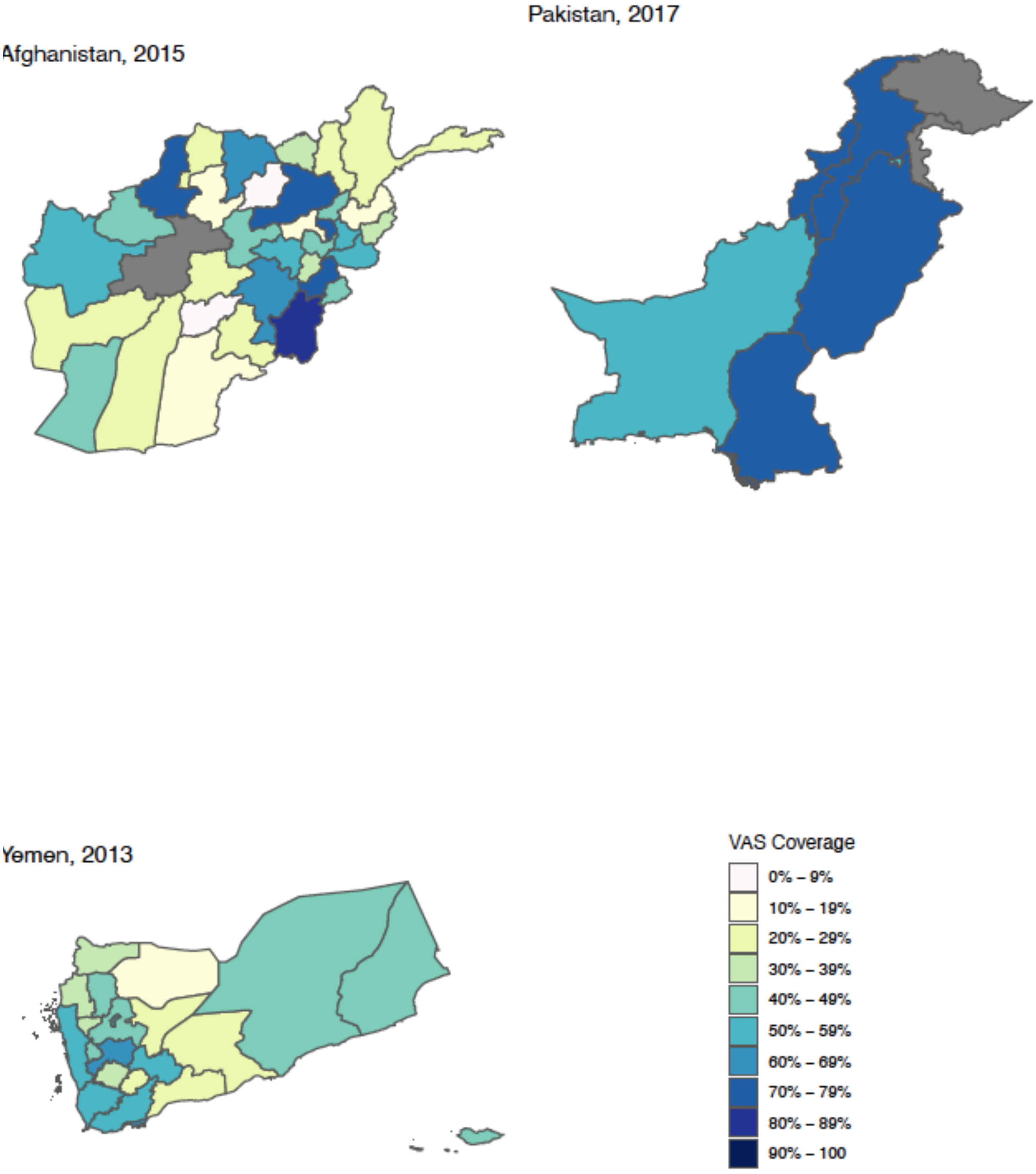

Supplementary Figure 5. Vitamin A supplementation (VAS) coverage between administrative regions for included countries in the Americas.

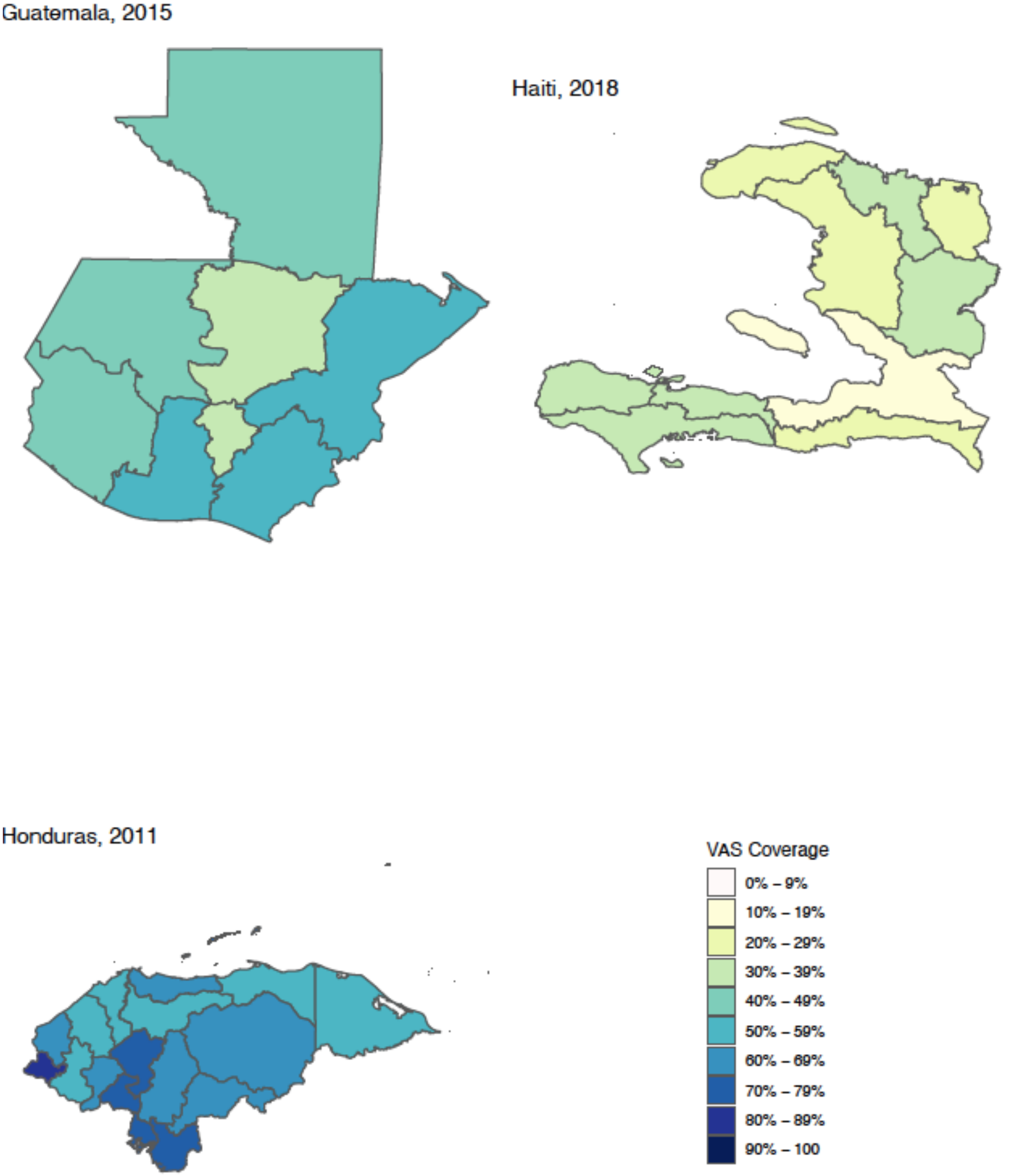

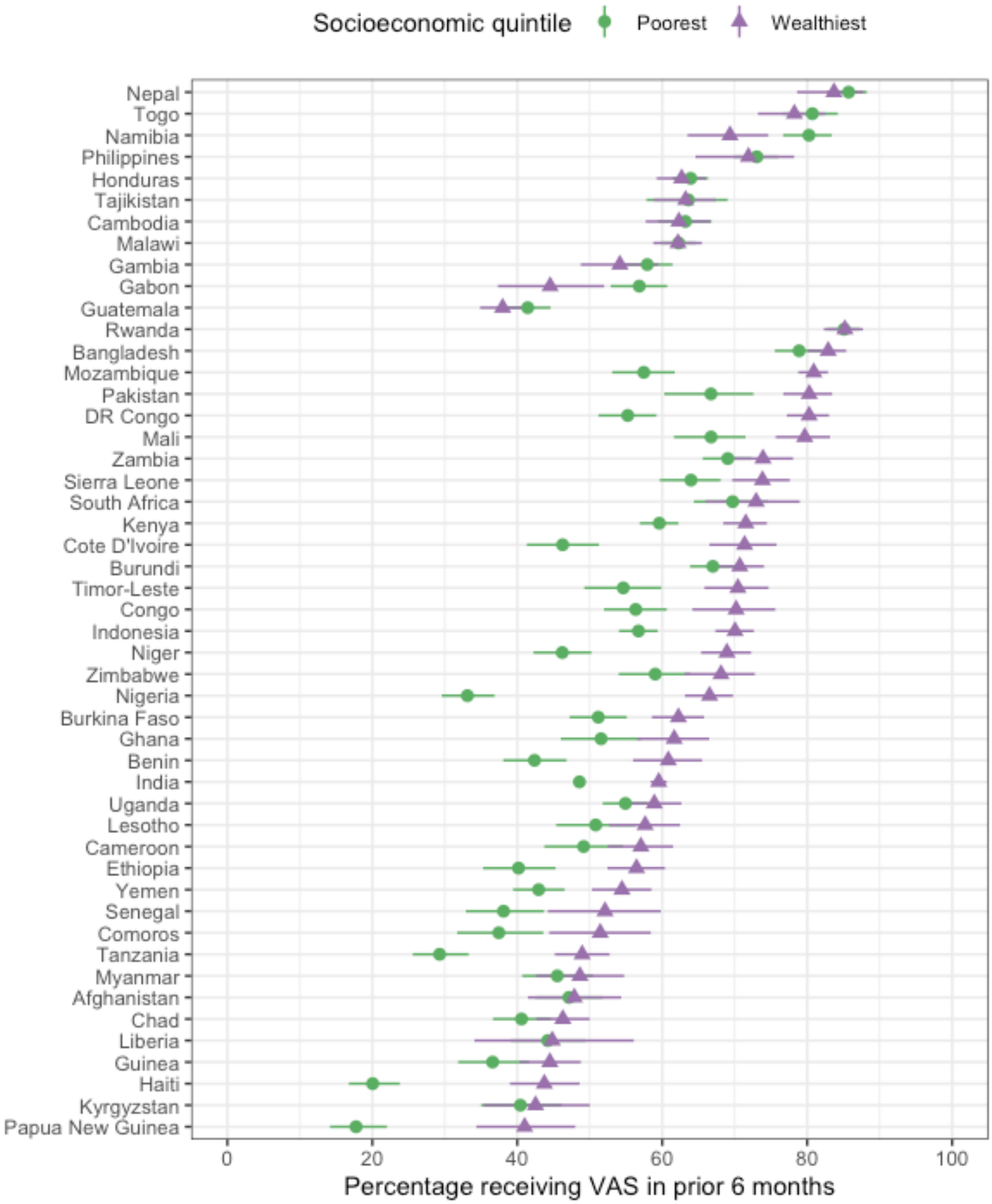

Supplementary Figure 6. Vitamin A supplementation (VAS) coverage among children from households of the poorest versus the richest socioeconomic quintiles.

For peer review only - http://bmjopen.bmj.com/site/about/guidelines.xhtml

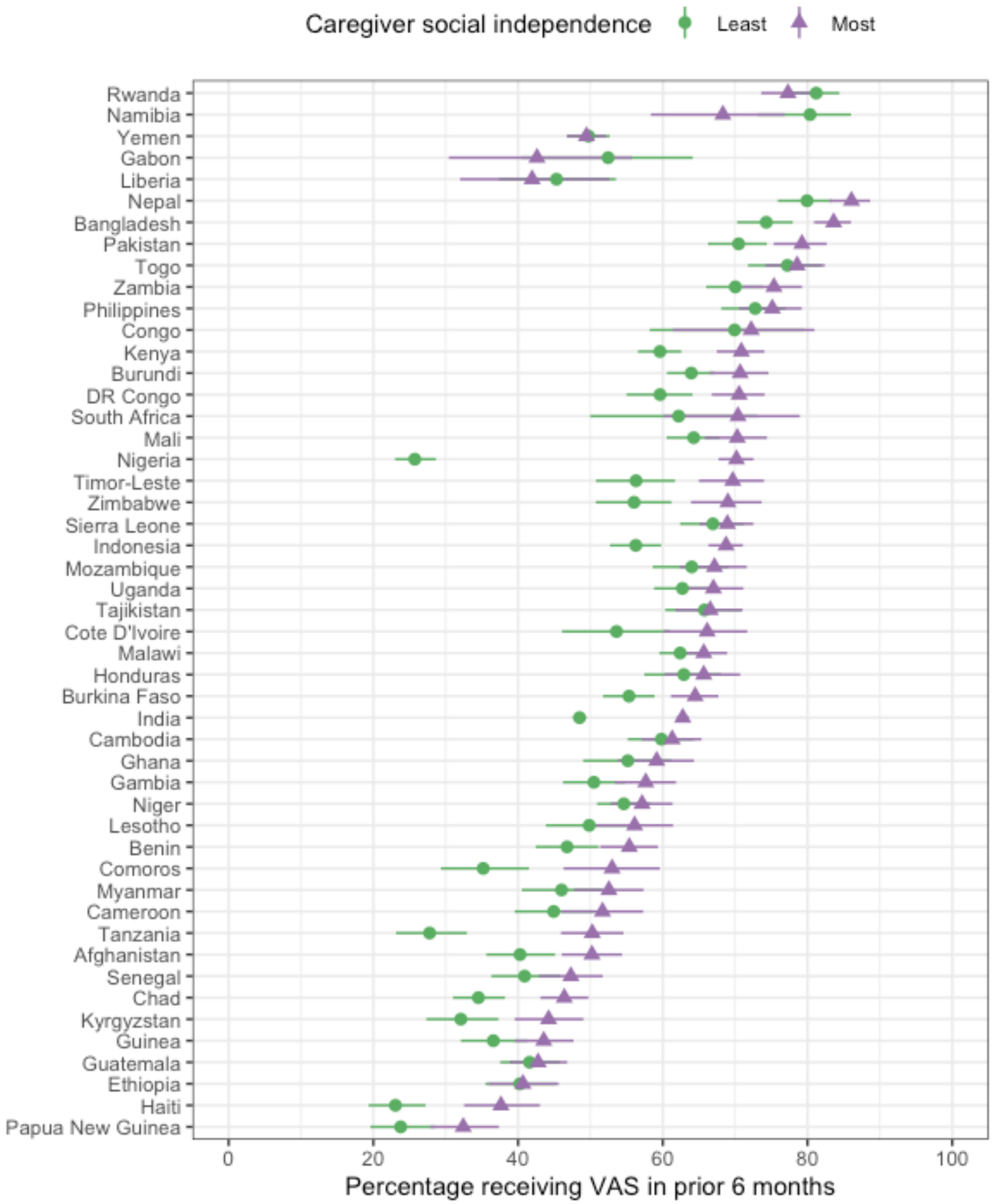

Supplementary Figure 7. Differences in vitamin A supplementation (VAS) coverage between children whose caregivers are the least versus the most socially independent in their country.

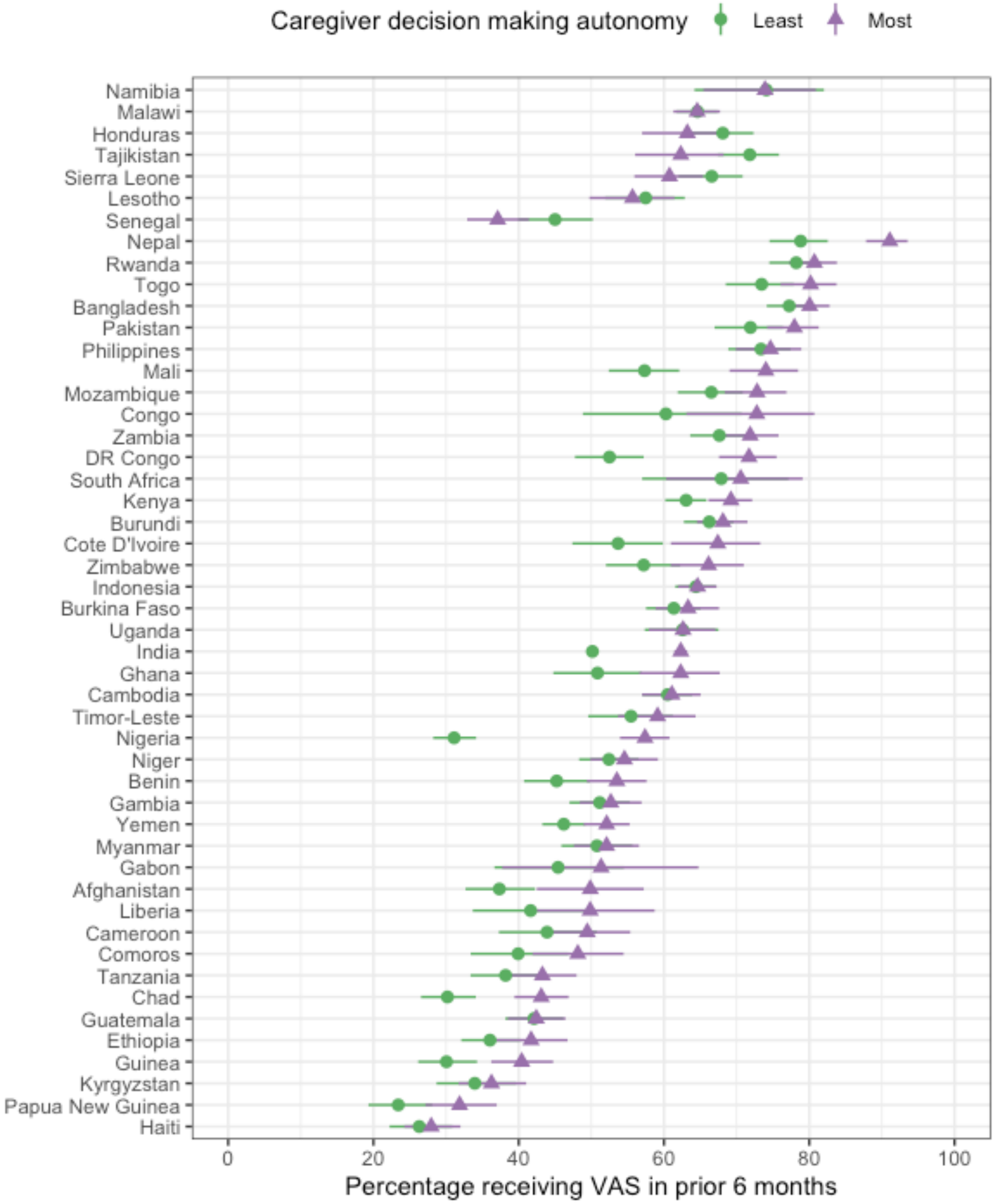

Supplementary Figure 8. Differences in vitamin A supplementation (VAS) coverage between children whose caregivers have the least versus the most decision-making autonomy in their country.

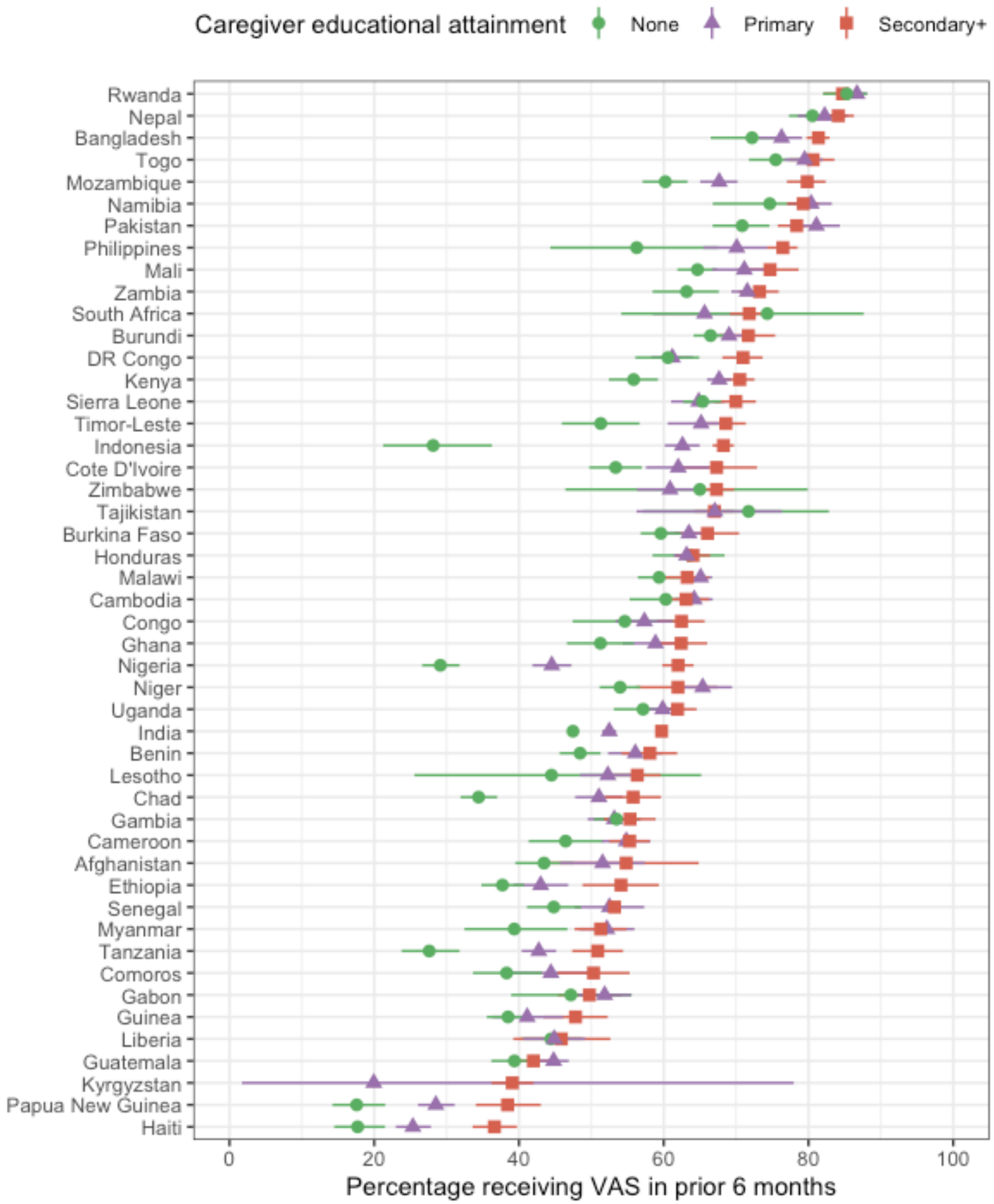

Supplementary Figure 9. Differences in vitamin A supplementation (VAS) coverage between children based on the educational attainment of the child's caregiver.

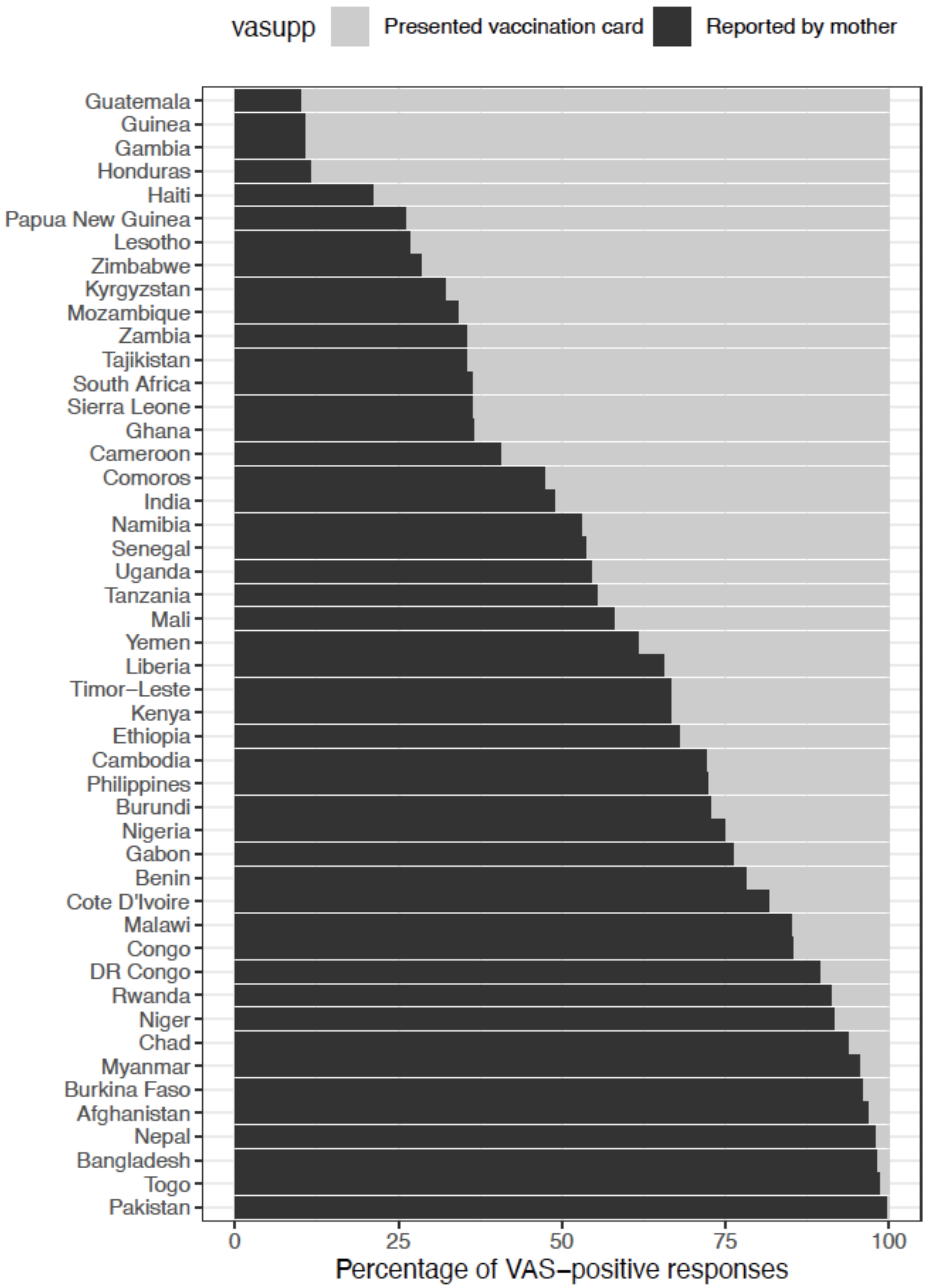

Supplementary Figure 10. Proportion of children who had received vitamin A supplementation (VAS) whose caregiver confirmed VAS reception with a vaccination card.

STROBE Statement—Checklist of items that should be included in reports of ***cross-sectional studies***

	Item No	Recommendation
**Title and abstract**	1	(*a*) Indicate the study's design with a commonly used term in the title or the abstract (p1-2)
		(*b*) Provide in the abstract an informative and balanced summary of what was done and what was found (p2-3)
**Introduction**		
Background/rationale	2	Explain the scientific background and rationale for the investigation being reported (p5-8)
Objectives	3	State specific objectives, including any prespecified hypotheses (p8)
**Methods**		
Study design	4	Present key elements of study design early in the paper (p10-11)
Setting	5	Describe the setting, locations, and relevant dates, including periods of recruitment, exposure, follow-up, and data collection (p9)
Participants	6	(*a*) Give the eligibility criteria, and the sources and methods of selection of participants (p9)
Variables	7	Clearly define all outcomes, exposures, predictors, potential confounders, and effect modifiers. Give diagnostic criteria, if applicable (p10-12)
Data sources/ measurement	8*	For each variable of interest, give sources of data and details of methods of assessment (measurement). Describe comparability of assessment methods if there is more than one group
Bias	9	Describe any efforts to address potential sources of bias
Study size	10	Explain how the study size was arrived at (p9)
Quantitative variables	11	Explain how quantitative variables were handled in the analyses. If applicable, describe which groupings were chosen and why (pg 10-12)
Statistical methods	12	(*a*) Describe all statistical methods, including those used to control for confounding (pg13)
		(*b*) Describe any methods used to examine subgroups and interactions (pg 13)
		(*c*) Explain how missing data were addressed (n/a)
		(*d*) If applicable, describe analytical methods taking account of sampling strategy (pg 13)
		(*e*) Describe any sensitivity analyses (n/a)
**Results**		
Participants	13*	(a) Report numbers of individuals at each stage of study—eg numbers potentially eligible, examined for eligibility, confirmed eligible, included in the study, completing follow-up, and analysed (p14, SuppTable 1)
		(b) Give reasons for non-participation at each stage (n/a secondary data)
		(c) Consider use of a flow diagram (n/a)
Descriptive data	14*	(a) Give characteristics of study participants (eg demographic, clinical, social) and information on exposures and potential confounders (p14, SuppTable 2)
		(b) Indicate number of participants with missing data for each variable of interest (p14)
Outcome data	15*	Report numbers of outcome events or summary measures (p14-15, SuppTable 2)
Main results	16	(*a*) Give unadjusted estimates and, if applicable, confounder-adjusted estimates and their precision (eg, 95% confidence interval). Make clear which confounders were adjusted for and why they were included (p15-16, Figures 1-4)

For peer review only

		(*b*) Report category boundaries when continuous variables were categorized (n/a)
		(*c*) If relevant, consider translating estimates of relative risk into absolute risk for a meaningful time period (n/a)
Other analyses	17	Report other analyses done—eg analyses of subgroups and interactions, and sensitivity analyses (the primary aim of this work was subgroup analysis p14-16)
**Discussion**		
Key results	18	Summarise key results with reference to study objectives (p17)
Limitations	19	Discuss limitations of the study, taking into account sources of potential bias or imprecision. Discuss both direction and magnitude of any potential bias (p20-21)
Interpretation	20	Give a cautious overall interpretation of results considering objectives, limitations, multiplicity of analyses, results from similar studies, and other relevant evidence (p17-18)
Generalisability	21	Discuss the generalisability (external validity) of the study results (p19-20)
**Other information**		
Funding	22	Give the source of funding and the role of the funders for the present study and, if applicable, for the original study on which the present article is based (p23)

*Give information separately for exposed and unexposed groups.

Note: An Explanation and Elaboration article discusses each checklist item and gives methodological background and published examples of transparent reporting. The STROBE checklist is best used in conjunction with this article (freely available on the Web sites of PLoS Medicine at http://www.plosmedicine.org/, Annals of Internal Medicine at http://www.annals.org/, and Epidemiology at http://www.epidem.com/). Information on the STROBE Initiative is available at www.strobe-statement.org.

For peer review only

For peer review only - http://bmjopen.bmj.com/site/about/guidelines.xhtml
